# Supplementary material for: The role of education in the association between self-rated health and levels of C-reactive protein: a cross-sectional study in rural areas of China
Source: BMJ Open. 2019 Nov 19;9(11):e027659. doi: 10.1136/bmjopen-2018-027659 (PMC6887060; doi:10.1136/bmjopen-2018-027659)
Supplement: Supplementary data [file bmjopen-2018-027659supp001.pdf]

SUPPLEMENTARY FILE

Table S1 Characteristics of study sample in NP without and with missing values in CRP

	Non-missing[a] (n=646)	Missing[a] (n=2)	P
Age			0.093
<60	267(41.3)	2(100)	
≥60	379(58.7)	0(0)	
Sex			0.245
Men	261(40.4)	0(0)	
Women	385(59.6)	2(100)	
Education			0.239
Illiterate	265(41.0)	0(0)	
Literate	381(59.0)	2(100)	
Marital status			0.491
Married	522(80.8)	2(100)	
Non-married	124(19.2)	0(0)	
Smoking			0.582
Current smokers	85(13.2)	0(0)	
Non-current smokers	561(86.8)	2(100)	
Drinking			0.489
Regular drinkers	125(19.4)	0(0)	
Non-regular drinkers	521(80.7)	2(100)	
BMI			0.810
Underweight (<18.5)	30(4.60)	0(0)	
Normal weight (18.5-25)	436(67.5)	2(100)	
Overweight (25-30)	158(24.5)	0(0)	
Obese (≥30)	22(3.4)	0(0)	
Self-rated health			0.184
Good	188(29.1)	2(100)	
Average	270(41.8)	0(0)	
Poor	165(25.5)	0(0)	
Very poor	23(3.6)	0(0)	
Health status[b]			0.018
Healthy	127(19.7)	2(100)	
Unhealthy	518(80.2)	0(0)	
Missing	1(0.2)	0(0)	

[a] Data are presented as n (%).
[b] Healthy status:
Unhealthy: Self-reported moderate to severe symptoms in the last month or used antihypertensive or antidiabetic medications (NP).
Healthy: No such report.

Table S2 Characteristics of study sample in CHARLS without and with missing values in CRP

	**Non-missing[a] (n=8555)**	**Missing[a] (n=3810)**	**P**
Age			0.002
<60	4703(55.0)	2226(58.4)	
≥60	3850(45.0)	1583(41.6)	
Missing	2(0)	1(0)	
Sex			<0.001
Men	4042(47.3)	2014(52.9)	
Women	4506(52.7)	1794(47.1)	
Missing	7(0.1)	2(0.1)	
Education			0.001
Illiterate	2835(33.1)	1160(30.5)	
Literate	5716(66.8)	2643(69.4)	
Missing	4(0.1)	7(0.2)	
Marital status			0.001
Married	7517(87.9)	3263(85.6)	
Non-married	1038(12.1)	547(14.4)	
Smoking			0.113
Current smokers	2561(29.9)	1086(28.5)	
Non-current smokers	5993(70.1)	2722(71.4)	
Missing	1(0)	2(0.1)	
Drinking			0.024
Regular drinkers	998(11.7)	399(10.5)	
Non-regular drinkers	7554(88.3)	3406(89.4)	
Missing	3(0)	5(0.1)	
BMI			<0.001
Underweight (<18.5)	535(6.3)	206(5.4)	
Normal weight (18.5-25)	4719(55.2)	1790(47.0)	
Overweight (25-30)	1819(21.3)	592(15.5)	
Obese (≥30)	291(3.4)	101(2.7)	
Missing	1191(13.9)	1121(29.4)	
Self-rated health			0.002
Good	1794(21.0)	910(23.9)	
Average	4157(48.6)	1798(47.2)	
Poor	2157(25.2)	894(23.5)	
Very poor	447(5.2)	208(5.5)	
Health status[b]			<0.001
Healthy	2089(24.4)	1160(30.5)	
Unhealthy	6401(74.8)	2607(68.4)	
Missing	65(0.8)	43(1.1)	

[a] Data are presented as n (%)

[b] Healthy status:

Unhealthy: Had been diagnosed by a doctor with any disease or often suffered from any pain currently (CHARLS).

Healthy: No such report.

Table S3 Association between self-rated health and C-reactive protein among illiterate and literate people: stratified by age and sex

	N	Age<60	N	Age≥60	N	Men	N	Women
**Illiterate**								
**NP**								
Good	47	Ref.	127	Ref.	56	Ref.	118	Ref.
Poor	16	0.35 (-0.27 to 0.97)	75	-0.01 (-0.32 to 0.29)	21	0.03(-0.53 to 0.59)	70	0.04 (-0.26 to 0.34)
**CHARLS**								
Good	777	Ref.	989	Ref.	406	Ref.	1359	Ref.
Poor	405	-0.07 (-0.19 to 0.05)	663	0.05 (-0.05 to 0.15)	232	-0.06(-0.24 to 0.11)	835	0.02 (-0.07 to 0.1)
**NP+CHARLS**								
Good	824	Ref.	1116	Ref.	462	Ref.	1477	Ref.
Poor	421	-0.05 (-0.17 to 0.07)	738	0.05 (-0.05 to 0.14)	253	-0.04(-0.2 to 0.13)	905	0.02 (-0.06 to 0.1)
**Literate**								
**NP**								
Good	161	Ref.	123	Ref.	136	Ref.	148	Ref.
Poor	43	0.47 (0.14 to 0.8)**	54	0.08 (-0.3 to 0.45)	48	0.4 (0.03 to 0.77)*	49	0.17 (-0.16 to 0.5)
**CHARLS**								
Good	2690	Ref.	1490	Ref.	2581	Ref.	1597	Ref.
Poor	829	0.11 (0.03 to 0.19)**	706	0.11 (0.02 to 0.19)*	821	0.16 (0.08 to 0.24)**	713	0.03 (-0.05 to 0.12)
**NP+CHARLS**								
Good	2851	Ref.	1613	Ref.	2717	Ref.	1745	Ref.
Poor	872	0.13 (0.06 to 0.21)**	760	0.11 (0.02 to 0.19)*	869	0.17 (0.1 to 0.25)**	762	0.04 (-0.04 to 0.13)

CHARLS: 1) Iliterate:1 missing in age, 3 missing in sex; 2) Literate: 1 missing in age, 4 missing in sex.

*P<0.05

**P<0.01
