# Supplementary material for: The role of education in the association between self-rated health and levels of C-reactive protein: a cross-sectional study in rural areas of China
Source: BMJ Open. 2019 Nov 19;9(11):e027659. doi: 10.1136/bmjopen-2018-027659 (PMC6887060; doi:10.1136/bmjopen-2018-027659)
Supplement: Author's manuscript [file bmjopen-2018-027659.draft_revisions.pdf]

BMJ Open is committed to open peer review. As part of this commitment we make the peer review history of every article we publish publicly available.

When an article is published we post the peer reviewers' comments and the authors' responses online. We also post the versions of the paper that were used during peer review. These are the versions that the peer review comments apply to.

The versions of the paper that follow are the versions that were submitted during the peer review process. They are not the versions of record or the final published versions. They should not be cited or distributed as the published version of this manuscript.

BMJ Open is an open access journal and the full, final, typeset and author-corrected version of record of the manuscript is available on our site with no access controls, subscription charges or pay-per-view fees (http://bmjopen.bmj.com).

If you have any questions on BMJ Open's open peer review process please email info.bmjopen@bmj.com

Self-rated health and levels of C-reactive protein in rural areas of China: the role of education

Journal:	*BMJ Open*
Manuscript ID	bmjopen-2018-027659
Article Type:	Research
Date Submitted by the Author:	01-Nov-2018

Complete List of Authors:	Tu, Raoping; Nagasaki University Institute of Tropical Medicine, Department of International Health<br>Pan, Kuan-Yu; Karolinska Institutet Department of Neurobiology Care Sciences and Society, Aging Research Center<br>Cai, Guoxi; Nagasaki Prefectural Institute of Environment and Public Health<br>Yamamoto, Taro; Nagasaki University Institute of Tropical Medicine, Department of International Health<br>Wang, Hui-Xin; Stressforskningsinstitutet; Karolinska Institutet Department of Neurobiology Care Sciences and Society, Aging Research Center

Keywords:	EPIDEMIOLOGY, PUBLIC HEALTH, SOCIAL MEDICINE

SCHOLARONE™
Manuscripts

Title page

Self-rated health and levels of C-reactive protein in rural areas of China: the role of education

Author's names and affiliation:

Raoping Tu [1,2], Kuan-Yu Pan [3], Guoxi Cai [1,4], Taro Yamamoto [1,*], Hui-Xin Wang [3,5,*]

[1] Department of International Health, Institute of Tropical Medicine (NEKKEN), Nagasaki University, Nagasaki, Japan.

[2] Leading Program, Graduate School of Biomedical Sciences, Nagasaki University, Nagasaki, Japan.

[3] Aging Research Center, Department of Neurobiology, Care Sciences and Society (NVS), Karolinska Institutet, Stockholm University, Stockholm, Sweden.

[4] Nagasaki Prefectural Institute of Environment and Public Health, Nagasaki, Japan.

[5] Stress Research Institute, Stockholm University, Stockholm, Sweden.

*Correspondence to:

1. Department of International Health, Institute of Tropical Medicine (NEKKEN), Nagasaki University, 1-12-4 Sakamoto, Nagasaki 852-8523, Japan

Tel: +81-095-819-7853, Fax: +81-095-819-7869

Taro Yamamoto

E-mail: y-taro@nagasaki-u.ac.jp

For peer review only

2. Stress Research Institute, Stockholm University, SE-106 91 Stockholm, Sweden

Tel: +46-08-5537-8941

Hui-Xin Wang

E-mail: huixin.wang@su.se

Word count: 2713

For peer review only

For peer review only - http://bmjopen.bmj.com/site/about/guidelines.xhtml

ABSTRACT

Objectives This study aims to examine the association between self-rated health (SRH) and levels of C-reactive protein (CRP) among adults aged 45 to 101 years old in rural areas of China, and to explore the role of education in the association.

Design Cross-sectional study

Setting The study population was derived from two databases in China: Nanping (Nanping project, NP) and the China Health and Retirement Longitudinal Study (CHARLS).

Participants 646 participants from a rural area of NP, and 8555 rural participants from CHARLS.

Methods CRP was measured using a high sensitivity sandwich enzyme immunoassay in NP and immunoturbidimetric assay in CHARLS. SRH was assessed by self-rated health questionnaires and categorized into good and poor. Education was measured by maximum years of schooling and dichotomized into illiterate and literate. Multivariate linear regression models were used to study the associations.

Results Compared to people with good SRH, those with poor SRH had higher levels of CRP in NP (β=0.16, 95%CI -0.02 to 0.34) and in CHARLS (β=0.07, 95%CI 0.02 to 0.11). This was especially in men and literate people after adjusting for potential confounders. Similar findings were observed in the pooled population (β=0.08, 95%CI 0.03 to 0.12), in men (β=0.13, 95%CI 0.06 to 0.20) and in literate people (β=0.12, 95%CI 0.06 to 0.18).

Conclusion Poor SRH may be a predicator of elevated levels of CRP among middle-

For peer review only - http://bmjopen.bmj.com/site/about/guidelines.xhtml

aged and older people in rural areas, especially in men and literate people.

Keywords Self-rated health; C-reactive protein; Education level

Strengths and limitations of this study

- Our study population comes from two databases, including one national representative sample derived from CHARLS, making our results highly generalizable to the national rural population of China.
- CRP is an objective measure performed by health professionals using validated methods, making it more reliable than subjective measures.
- The application of both linear and logistic regressions ensured our confidence in the findings and facilitated the interpretation of the results.
- It is a cross-sectional study design, thus it is difficult to demonstrate the temporality.
- Convenient sampling in the NP study may bias the results towards over estimation, because the small sample size may limit the study power and increase the risk of false positive findings. Thus, the results from the NP study should be referred to with caution. However, similar results were observed using a national representative sample from CHARLS.

For peer review only - http://bmjopen.bmj.com/site/about/guidelines.xhtml

Text

INTRODUCTION

C-reactive protein (CRP), a marker of systemic inflammation, has been shown to be involved in crucial pathogenesis in a variety of negative health outcomes, including cardiovascular diseases,[1, 2] diabetes,[3] cancer,[4] and cognitive decline.[5] Since the value of CRP in the prediction of prognoses in health outcomes has been recognized, it is important, from a public health perspective, to identify people at risk of elevated CRP in an efficient and simple way.

It is well known that self-rated health (SRH) can be simply measured through an individual's subjective perception of his own health, thus many health authorities have introduced SRH for surveillance.[6] SRH has been featured as a strong predictor for functional ability,[7] onset of chronic diseases,[8] and mortality.[9, 10] The association between SRH and CRP has been tested in previous works, but the results have been inconsistent.[11-14] These discrepancies may be due to differences in characteristics of the study populations. For example, a Japanese study demonstrated an association between poor SRH and elevated CRP value in women, but not in men (age range 40-69).[14] Among hospital-based studies, poor SRH was associated with elevated CRP in female patients (mean age 63.3±8.7/62.5±8.9 in control/intervention group) with coronary heart disease,[12] but not in some patients with breast cancer (mean age 55.2±8.4).[15]

It is noteworthy that studies concerning the association between SRH and CRP were mostly conducted in developed countries where the study populations were relatively well educated.[11-14] It has been shown that people with different education levels have different perceptions of health.[16] This suggests that the association between SRH and CRP may also be different

For peer review only - http://bmjopen.bmj.com/site/about/guidelines.xhtml

among people with different educational levels.[17] However, to our knowledge, no study has focused on the difference between illiterate and literate people. In China, despite the decrease in illiteracy from 1990 to 2010, there continues to be large differences between urban and rural areas: the rate of illiteracy in rural areas is more than two times that of urban areas.[18] Considering the lack of resources in rural areas, identifying people at risk of negative health outcomes using a simple measure such as SRH is warranted.

In the current study, we use two databases from China: to examine the association between SRH and CRP among middle-aged and older people in rural areas, and to explore whether the SRH-CRP association varies across age, sex and education levels.

METHODS

Study population

Nanping project (NP)

NP is a 2015, voluntary participation, cross-sectional study consisting of residents aged 18 years or older from one county of Nanping City in Fujian Province, China. Seven villages were selected based on recommendations from local health workers, since the residents in these areas are known to be highly cooperative.

As showed in Figure 1, a total of 797 people were enrolled. To match with the age range of study population from the CHARLS, we excluded 98 participants under 45 years old. Those with CRP concentrations higher than 6.25 mg/L in dried blood spots (DBS), which is comparable to 10 mg/L at serum level[19] (n=25), were excluded due to potential acute inflammatory conditions. After further excluding people with missing information on CRP values (n=3) and SRH (n=25), 646 people remained in our current study.

For peer review only - http://bmjopen.bmj.com/site/about/guidelines.xhtml

China Health and Retirement Longitudinal Study (CHARLS)

The CHARLS is a nationally representative longitudinal study. Eligible people were selected through a multistage probability sampling, and detailed descriptions of sampling method are provided in the users' guide.[20] In this current study, we used data from the baseline survey in 2011 because the CRP data was only available in that year. This is a secondary analysis of the CHARLS public database.

Overall, 17430 people were examined at baseline (Figure 1). People who lived in communities, or in both villages and communities (n=4562), and had CRP>10mg/L (n=429) were excluded. We further excluded people with missing data on CRP (n=3810) and SRH (n=74). Finally, 8555 (69%) people were included in our analysis.

Self-rated health (SRH)

SRH was assessed by one question: 'In general how would you rate your health?' Response options were 'good', 'average', 'poor', and 'very poor'.

C-reactive protein (CRP)

NP

Finger prick blood samples were collected by health workers using a filter paper, known as DBS. We kept the DBS at room temperature for a few days after being desiccated during the investigation period, then stored them in the Fujian Medical University at –20°. We used high sensitivity sandwich enzyme immunoassay method to measure CRP concentrations by applying monoclonal antibodies.[19] Further details of the protocols have been presented elsewhere.[21]

For peer review only - http://bmjopen.bmj.com/site/about/guidelines.xhtml

CHARLS

The venous blood samples were collected by trained staff from local Chinese Center for Disease Control and Prevention (China CDC). Plasma samples were collected and preserved in 0.5 mL cryovial at -20°C, delivered to Beijing CDC within 2 weeks. Plasma CRP was determined by the immunoturbidimetric assay method at Capital Medical University.[22]

Covariates

In both cohorts, all participants were interviewed face-to-face by trained interviewers using a questionnaire that covers information on age, sex, education, marital status, smoking, alcohol consumption, and health status. Height and weight were measured by interviewers using standard anthropometers.

Education level was determined by maximum years of schooling: 0 year (illiterate), 1-6 years (elementary school), 7-9 years (junior high school), 10-12 years (senior high school), >12 years (college or above). Due to the fact that more than 30% of both the NP and CHARLS samples were illiterate, we dichotomized education into 0 year (illiterate) and >0 year (literate). Age was dichotomized as 45-60 years versus ≥60 years old, and marital status as married versus non-married. BMI was calculated by dividing weight (kg) by height squared (m^2) and categorized as underweight (<18.5), normal weight (18.5-24.99), overweight (25-29.99), and obese (≥30). Smoking was dichotomized into current smokers and non-current smokers (including former smokers). Alcohol consumption was categorized as regular drinkers (more than 3 times per week) and non-regular drinkers.

Health status was measured by asking the participants whether they had any moderate/severe

For peer review only - http://bmjopen.bmj.com/site/about/guidelines.xhtml

disease symptoms (e.g., fever) in the last month, or used antihypertensive or antidiabetic medications in the NP, and whether they had ever been diagnosed by a doctor with any diseases (e.g., hypertension), or often suffered from any pain currently in CHARLS. People answering positively were categorized as unhealthy, otherwise healthy.

Statistical analysis

First, data from the NP and CHARLS were analyzed separately. We applied one-way ANOVA to examine the differences of CRP in characteristics in each dataset by using F-distribution. The CRP variable was log-transformed because it was not normally distributed. The association between SRH and CRP was estimated by β-coefficient and a 95% confidence interval (CI) using linear regression in two datasets. The first estimate was respective; in the second, datasets were pooled. Fixed-effect meta-analysis was used to examine the heterogeneity. Then we re-ran the linear regression using the pooled dataset.

Next, to facilitate the interpretation of the association between SRH and CRP, multivariate logistic analysis was performed to estimate odds ratios (ORs) and 95% CIs in the two datasets separately. We categorized CRP into two levels: low (<3mg/L) and high (≥3mg/L).[23] Using fixed-effect meta-analysis to examine the heterogeneity of two datasets again. Later, logistic regression was conducted in the combined population.

Age, sex and education were introduced into the basic-adjusted model. Further, we additionally adjusted for marital status, smoking, alcohol consumption, BMI, and health status.[24, 25] All analyses were repeated in the stratified analyses by age, sex and education.

In addition, we conducted multiple imputation for missing data. For further sensitivity analyses,

For peer review only - http://bmjopen.bmj.com/site/about/guidelines.xhtml

we performed additional analyses: 1) We adjusted for psychological distress in the NP and depression in CHARLS; 2) We used sampling weights to derive national estimates in CHARLS;[20] 3) We re-ran linear regression after excluding illiterate participants in order to compare with previous studies; 4) Since the social economic status-psychological well-being association was strong in poor areas,[26] we further adjusted for self-rated household income in the NP and self-rated household living standards in CHARLS as their assessment of social economic status were different.

All statistical analyses were performed with Stata 13.0 (Stata Corp, College Station, TX, USA).

RESULTS

Characteristics of the participants

The CRP levels across different characteristics of participants were compared in each dataset separately. Table 1 shows that in both datasets that older age people, higher BMI's, poorer SRH, or an unhealthy status were more likely to have elevated levels of CRP. The findings were inconsistent with sex, education, marital status, smoking and alcohol consumption in the two datasets. People with missing CRP values in NP and CHARLS were better educated and reported better health status compared to those who remained in the analyses (data not shown).

For peer review only - http://bmjopen.bmj.com/site/about/guidelines.xhtml

For peer review only

Table 1 CRP values across characteristics of the study population

	NP (n=646)			CHARLS (n=8555)		
	CRP[a]	F	P	CRP	F	P
Age		(1, 644)=14.04	<0.001		(1, 8551)=89.38	<0.001
45-60	0.6 (0.3 to 1.2)			0.9 (0.5 to 1.7)		
≥60	0.8 (0.4 to 1.8)			1.1 (0.6 to 2.1)		
Sex		(1, 644)=6.53	0.011		(1, 8546)=8.94	0.003
Men	0.6 (0.3 to 1.3)			1.0 (0.5 to 2.0)		
Women	0.8 (0.4 to 1.7)			0.9 (0.5 to 1.8)		
Education		(1, 644)=8.46	0.004		(1, 8549)=1.01	0.316
Illiterate	0.9 (0.4 to 1.8)			1.0 (0.5 to 2.0)		
Literate	0.6 (0.3 to 1.3)			0.9 (0.5 to 1.9)		
Marital status		(1, 644)=0.47	0.495		(1, 8553)=22.48	<0.001
Married	0.7 (0.3 to 1.5)			0.9 (0.5 to 1.9)		
Non-married	0.7 (0.4 to 1.7)			1.1 (0.6 to 2.4)		
Smoking		(1, 644)=0.53	0.467		(1, 8552)=4.20	0.041
Current smokers	0.6 (0.3 to 1.4)			1.0 (0.5 to 2.0)		
Non-current smokers	0.7 (0.4 to 1.6)			0.9 (0.5 to 1.9)		
Alcohol consumption		(1, 644)=10.94	0.001		(1, 8550)=0.23	0.635
Regular drinkers	0.5 (0.3 to 1.1)			0.9 (0.5 to 1.9)		
Non-regular drinkers	0.8 (0.4 to 1.6)			1.0 (0.5 to 1.9)		
BMI		(3, 642)=17.91	<0.001		(3, 7360)=93.38	<0.001
Underweight (<18.5)	0.5 (0.2 to 1.4)			0.8 (0.5 to 1.9)		
Normal weight (18.5-25)	0.6 (0.3 to 1.1)			0.8 (0.5 to 1.7)		
Overweight (25-30)	1.2 (0.6 to 2.3)			1.2 (0.7 to 2.3)		

For peer review only - http://bmjopen.bmj.com/site/about/guidelines.xhtml

Obese (≥30)	1.6 (1.0 to 4.4)			1.9 (0.9 to 3.3)		
Self-rated health		(3, 642)=2.36	0.071		(3, 8551)=10.63	<0.001
Good	0.6 (0.3 to 1.7)			0.9 (0.5 to 1.8)		
Average	0.7 (0.3 to 1.5)			0.9 (0.5 to 1.8)		
Poor	0.8 (0.4 to 1.5)			1.0 (0.6 to 2.1)		
Very poor	1.0 (0.5 to 2.3)			1.1 (0.6 to 2.3)		
Health status[b]		(1, 643)=9.47	0.002		(1, 8488)=38.03	<0.001
Healthy	0.5 (0.3 to 1.3)			0.8 (0.5 to 1.7)		
Unhealthy	0.8 (0.4 to 1.6)			1.0 (0.5 to 2.0)		

[a] Median (interquartile range); comparison was done with log-transformed values.

[b] Health status:

Unhealthy: Self-reported moderate to severe symptoms in the last month or used antihypertensive or antidiabetic medications (NP); Had been diagnosed by a doctor with any disease or often suffered from any pain currently (CHARLS). Healthy: no such report.

[c] Missing values:
NP: 1 missing in health status.
CHARLS: 2 missing in age, 7 missing in sex, 4 missing in education,1 missing in smoking, 3 missing in alcohol consumption, 1191 missing in BMI, 65 missing in health status.

For peer review only

SRH and CRP

Table 2 presents the association between SRH and CRP in the two individual populations. In the NP, a borderline statistically significant association was observed between very poor SRH and elevated levels of CRP (β=0.39, 95%CI -0.07 to 0.85) in basic-adjusted model, while the association was attenuated after adjusting for confounders (β=0.29, 95%CI -0.15 to 0.73). In CHARLS, poor and very poor SRH were both associated with higher CRP (β=0.06, 95%CI 0 to 0.12; β=0.11, 95%CI 0.01 to 0.22). As there were similar effects on CRP in both two datasets, we combined 'good' and 'average' as good SRH, 'poor' and 'very poor' as poor SRH, and found that poor SRH was statistically significantly associated with higher levels of CRP both in NP (β=0.16, 95%CI -0.02 to 0.34) and CHARLS (β=0.07, 95%CI 0.02 to 0.11) (Table 2).

Table 2 Association between self-rated health and C-reactive protein

		**Model1[a]**		**Model2[b]**	
	**N**	**β (95%CI)**	**P**	**β (95%CI)**	**P**
**NP**					
Good	188	Ref.		Ref.	
Average	270	-0.03 (-0.22 to 0.17)	0.792	-0.05 (-0.24 to 0.14)	0.589
Poor	165	0.12 (-0.10 to 0.34)	0.292	0.10 (-0.11 to 0.32)	0.349
Very Poor	23	0.39 (-0.07 to 0.85)	0.093	0.29 (-0.15 to 0.73)	0.202
Good/Poor	458/188	0.17 (-0.01 to 0.35)	0.067	0.16 (-0.02 to 0.34)	0.077
**CHARLS**					
Good	1794	Ref.		Ref.	
Average	4157	0.01 (-0.04 to 0.06)	0.613	0 (-0.05 to 0.06)	0.911
Poor	2157	0.10 (0.04 to 0.15)	0.001	0.06 (0 to 0.12)	0.055
Very Poor	447	0.16 (0.06 to 0.25)	0.001	0.11 (0.01 to 0.22)	0.036
Good/Poor	5951/2604	0.10 (0.05 to 0.14)	<0.001	0.07 (0.02 to 0.11)	0.004
**NP+CHARLS**					
Good/Poor	6409/2792	0.11 (0.06 to 0.15)	<0.001	0.08 (0.03 to 0.12)	0.001

[a] Adjusted for age, sex, education

[b] Adjusted for age, sex, education, marital status, smoking, alcohol consumption, BMI, health status

As the same direction and a very low level of heterogeneity (I-squared< 0.001%) were observed in the two datasets (data not shown), we pooled the data and re-ran the linear regression

For peer review only - http://bmjopen.bmj.com/site/about/guidelines.xhtml

analyses in the combined populations. The statistically significant SRH-CRP association was observed again in the pooled population (β=0.08, 95%CI 0.03 to 0.12) (Table2).

The roles of age, sex, and education in the association between SRH and CRP

The association between SRH and CRP stratified by age, sex, education is showed in Figure 2. In middle-aged people, worse SRH was associated with higher CRP both in NP (β=0.42, 95%CI 0.14 to 0.71) and CHARLS (β=0.06, 95%CI -0.01 to 0.12). Among older people, a similar trend was observed in CHARLS (β=0.08, 95%CI 0.02 to 0.15), but not in the NP. When stratified by sex, we found a statistically significant SRH-CRP association among men both in NP (β=0.27, 95%CI -0.03 to 0.57) and CHARLS (β=0.12, 95%CI 0.05 to 0.19), but not in women. In a stratified analysis by education, the association between SRH and CRP was seen in literate people both in NP (β=0.26, 95%CI 0.02 to 0.51) and CHARLS (β=0.11, 95%CI 0.05 to 0.16), but not in illiterate people.

In the pooled population, the SRH-CRP association was repeated in the middle-aged (β=0.08, 95%CI 0.02 to 0.14), older people (β=0.08, 95%CI 0.02 to 0.15), men (β=0.13, 95%CI 0.06 to 0.20), and literate people (β=0.12, 95%CI 0.06 to 0.18) (Figure 2).

Furthermore, we applied a logistic regression based on the pooled data. The odds ratio (OR) for having elevated levels of CRP in those with poor SRH was 1.18 in the total population (95%CI 1.03 to 1.37), 1.26 in men (95%CI 1.02 to 1.56), and 1.23 in the literate people (95%CI 1.03 to 1.48). Similar ORs were observed in the middle-aged and older people (Supplementary File: Table S1).

Additional analysis

For peer review only - http://bmjopen.bmj.com/site/about/guidelines.xhtml

Similar results were observed using data from multiple imputations for missing data and from sensitivity analyses further adjusting for psychological distress in the NP and depression in CHARLS. Identical trends of age and sex differences as main results were observed in literate people in both datasets, but not in the illiterate. Additional adjustment for socio-economic status did not result in any further changes.

Patient and public involvement

There were no participants involved in the development of this study.

DISCUSSION

In this study, based on 9201 rural area residents from two databases, we found statistically significant associations between poor SRH and elevated levels of CRP in middle-aged and older people, especially in men and the literate.

Our finding that poorer SRH is associated with elevated levels of CRP is in line with previous studies that included participants with similar age ranges as our study participants.[11, 14] In addition, we found that poor SRH was associated with elevated CRP level in literate participants, but not in illiterate ones, which was consistent with one previous study.[17] Indeed, similar results were also shown in studies on SRH and mortality. [27, 28] The likely explanation may be that illiterate people tend to have poorer health-related knowledge and access to health care,[16] and thus may misinterpret the feeling that they have in health. [29] It has been shown that poor SRH in the less educated people mainly represents less serious diseases.[30] In fact, we found that illiterate people were more likely to rate their health as poor and to report illness or pain both in NP and CHARLS (Supplementary File: Table S2). In addition, illiterate people may have to withstand more pressure as they have less social and

For peer review only - http://bmjopen.bmj.com/site/about/guidelines.xhtml

financial resources, thus, other factors rather than actual health condition may contribute to the reported poor SRH.

The association between poor SRH and elevated levels of CRP among older people (aged≥60 years) was observed in CHARLS, but not in NP. And in both populations, poor SRH was only associated with elevated levels of CRP in men, not in women. These findings may also be explained by education levels in each subgroup. That is, the proportion of illiterate people was relatively higher in older adults in NP (76.2%) than in CHARLS (58.3%) as shown in Table S2 (Supplementary File), and there was a higher proportion of illiterate women in both populations. Furthermore, after excluding the illiterate people, we observed similar age and sex differences in the associations between SRH and CRP among the literate people, which was the same as in the main results. This suggests that education might play a role in the SRH-CRP association. In addition, consistent findings were also observed in urban areas of CHARLS (data not shown), furthermore, adjusting for social economic status did not change SRH-CRP association (data not shown), suggesting social economic status may not be a major contributor to the SRH-CRP association.

We found that SRH-CRP associations were only observed in men, and not in women. Possibly this sex-differential finding was bound to the differences in reporting SRH by sex. Previous studies have shown that the poor SRH in women can reflect both serious and non-serious diseases, whereas in men it tends to reflect serious diseases.[31] Broad dimensions of health perceptions may lead to lesser accuracy of SRH in women. Second, educational difference between sexes can well explain the different findings between our study and the Iwate-KENCO study in Japan.[14] Our study population consisted of rural people in China with features of low literacy, especially in women, whereas in the Iwate-KENCO study, almost half of the

For peer review only - http://bmjopen.bmj.com/site/about/guidelines.xhtml

participants had more than 9 years of schooling.

SRH is an inclusive and dynamic evaluation of physical and psychological health, and social status. It has been shown that SRH may reflect an individual's resources (e.g., education level),[32] influence stress levels and health behaviors (e.g., physical activity), and affect immune function.[33] Poor SRH may also reflect a poor current physical (e.g., inaccessibility to health service) and social environment (e.g., limited social network), these negative circumstances can limit ones coping ability and produce psychological stress. It is known that stress can activate the sympathetic nervous system and the hypothalamic-pituitary-adrenal axis, and contribute to the production of stress hormones, leading to the secretion of CRP.[34, 35] In addition, people with poor SRH were more likely to be physically inactive,[36] and having an inactive lifestyle has been suggested to potentially weaken the immune system and facilitate the inflammation process through the release of pro-inflammatory adipokines.[37] Furthermore, poor SRH may also reflects poor medication adherence,[38] such as low aspirin adherence, which has been associated with elevated levels of CRP in the first 3 months after acute coronary syndrome.[39]

CONCLUSION

This study provides evidence that SRH, a simple measure, may be used as an indicator of ill-physical health among middle-aged and older literate people, but not among the illiterate people, in rural area. Future studies are needed to confirm our results and extend these findings to larger and more diverse populations, or with other health outcomes. Identification of simple health indictors for illiterate people are warranted.

What is already known on this subject?

For peer review only - http://bmjopen.bmj.com/site/about/guidelines.xhtml

- Inconsistent findings of the association between self-rated health and C-reactive protein in developed countries.
- Currently, no study concerning the difference between literate and illiterate people on the self-rated health and C-reactive protein association.

What this study adds?

- Self-rated health may serve as a relevant health predictor for people living in rural areas of developing countries.
- Poor self-rated health is associated with elevated levels of C-reactive protein in literate people, but not in the illiterate people. This suggests that education can improve the implementation and accuracy of SRH measurement by facilitating the understanding of correct health concepts.

Acknowledgments We would like to express our sincere gratitude to the participants and local staff in NP project. We are grateful for those supporters: Fukui University (Aki Yazawa); National Center for Global Health and Medicine, Japan (Yosuke Inoue); Nagasaki Prefectural Institute of Environment and Public Health (Guoxi Cai); Fujian Medical University (Fei He, Jie Chen); Fujian Provincial Center for Disease Control and Prevention (Meng Huang) during the data collection in NP Project. Data from China Health and Retirement Longitudinal Study (CHARLS) were collected by the National School of Development at Peking University, China. We appreciated to University of Copenhagen (Tianwei Xu); Fujian Provincial Center for Disease Control and Prevention (Xiuquan Lin); Nagasaki University (Sabin Nundu) for providing valuable comments in analysis and interpretation of data.

Contributors HW, KP and RT conceptualized the study. RT analyzed the data and drafted the manuscript. HW, KP, GC, TY contributed to critical revisions of the manuscript. RT and HW

For peer review only - http://bmjopen.bmj.com/site/about/guidelines.xhtml

For peer review only

are responsible for ensuring the integrity and accuracy of the study. All authors have read and approved the final manuscript.

Funding This study is financed by the Program for Nurturing Global Leaders in Tropical and Emerging Communicable Diseases, Graduate School of Biomedical Sciences, Nagasaki University, Japan (Raoping Tu); the Stockholm University (Hui-Xin Wang); the Ministry of Education of Taiwan, the Swedish National Graduate School on Ageing and Health (SWEA), and Gamla Tjänarinnor Foundation (Kuan-Yu Pan). NP Project was financially supported by the JSPS KAKENHI from the Japan Society for the Promotion of Science (13J06172).

Competing Interests None declared.

Patient consent Not required.

Ethics approval The Ethics Committee for Medical Research at the University of Tokyo (No. 10515-(1)) and the Ethics Committee of the Institute of Tropical Medicine at Nagasaki University (No. 120910100-5) approved the study protocol of NP. The Medical Ethics Committee of Peking University approved the research protocol of CHARLS.

Provenance and peer review Not commissioned; externally peer reviewed

Data sharing statement All of the CHARLS data will be accessible to researchers around the world at at the CHARLS project website (http://charls.pku.edu.cn/en). No additional data available.

For peer review only - http://bmjopen.bmj.com/site/about/guidelines.xhtml

For peer review only

Figure legends

Figure 1 Flowchart of the study populations in NP and CHARLS

Figure 2 Linear association between poor self-rated health and elevated levels of CRP in NP, CHARLS, and combined populations of NP and CHARLS: stratified by age, sex and education. SRH is dichotomized into two groups (poor to very poor versus good to average). Models are simultaneously adjusted for age, sex, education, marital status, smoking, alcohol consumption, BMI, health status.

SUPPLEMENTARY FILE

Table S1 Association between self-rated health and levels of C-reactive protein: stratified by age, sex and education (combined population, logistic)

Table S2 Characteristics of the study sample: stratified by age, sex, education

For peer review only

References

1 Black PH, Garbutt LD. Stress, inflammation and cardiovascular disease. *Journal of psychosomatic research* 2002;52:1-23.

2 Ridker PM. From C-Reactive Protein to Interleukin-6 to Interleukin-1. *Circulation Research* 2016;118:145-56.

3 Barzilaym JI, Freedland ES. Inflammation and its relationship to insulin resistance, type 2 diabetes mellitus, and endothelial dysfunction. *Metabolic syndrome and related disorders* 2003;1:55-67.

4 Wang J, Lee IM, Tworoger SS*, et al.* Plasma C-reactive protein and risk of breast cancer in two prospective studies and a meta-analysis. *Cancer Epidemiol Biomarkers Prev* 2015;24:1199-206.

5 Panza F, Solfrizzi V, Logroscino G*, et al.* Current epidemiological approaches to the metabolic-cognitive syndrome. *J Alzheimers Dis* 2012;30 Suppl 2:S31-75.

6 Salomon JA, Nordhagen S, Oza S*, et al.* Are Americans feeling less healthy? The puzzle of trends in self-rated health. *Am J Epidemiol* 2009;170:343-51.

7 Idler EL, Kasl SV. Self-ratings of health: do they also predict change in functional ability? *J Gerontol B Psychol Sci Soc Sci* 1995;50:S344-53.

8 Latham K, Peek CW. Self-Rated Health and Morbidity Onset Among Late Midlife U.S. Adults. *The Journals of Gerontology Series B: Psychological Sciences and Social Sciences* 2012;68:107-16.

9 DeSalvo KB, Bloser N, Reynolds K*, et al.* Mortality prediction with a single general self-rated health question. A meta-analysis. *J Gen Intern Med* 2006;21:267-75.

10 Singh-Manoux A, Dugravot A, Shipley MJ*, et al.* The association between self-rated health and mortality in different socioeconomic groups in the GAZEL cohort study. *Int J Epidemiol*

For peer review only

For peer review only - http://bmjopen.bmj.com/site/about/guidelines.xhtml

2007;36:1222-8.

11 Christian LM, Glaser R, Porter K, *et al.* Poorer self-rated health is associated with elevated inflammatory markers among older adults. *Psychoneuroendocrinology* 2011;36:1495-504.

12 Janszky I, Lekander M, Blom M, *et al.* Self-rated health and vital exhaustion, but not depression, is related to inflammation in women with coronary heart disease. *Brain Behav Immun* 2005;19:555-63.

13 Shanahan L, Bauldry S, Freeman J, *et al.* Self-rated health and C-reactive protein in young adults. *Brain, Behavior, and Immunity* 2014;36:139-46.

14 Tanno K, Ohsawa M, Onoda T, *et al.* Poor self-rated health is significantly associated with elevated C-reactive protein levels in women, but not in men, in the Japanese general population. *Journal of Psychosomatic Research* 2012;73:225-31.

15 Orre IJ, Reinertsen KV, Aukrust P, *et al.* Higher levels of fatigue are associated with higher CRP levels in disease-free breast cancer survivors. *J Psychosom Res* 2011;71:136-41.

16 Spuling SM, Huxhold O, Wurm S. Predictors of Self-Rated Health: Does Education Play a Role Above and Beyond Age? *J Gerontol B Psychol Sci Soc Sci* 2017;72:415-24.

17 Dowd JB, Zajacova A. Does self-rated health mean the same thing across socioeconomic groups? Evidence from biomarker data. *Ann Epidemiol* 2010;20:743-9.

18 Guo S, Guo Y. *Spotlight on China: Changes in education under China's market economy*: Springer 2016.

19 Brindle E, Fujita M, Shofer J, *et al.* Serum, plasma, and dried blood spot high-sensitivity C-reactive protein enzyme immunoassay for population research. *J Immunol Methods* 2010;362:112-20.

20 Zhao Y, Strauss J, Yang G, *et al. China health and retirement longitudinal study–2011–2012 national baseline users' guide*. Beijing: National School of Development, Peking University 2013.

For peer review only - http://bmjopen.bmj.com/site/about/guidelines.xhtml

21 Yazawa A, Inoue Y, Cai G, *et al.* Epstein-Barr virus antibody titer as a stress biomarker and its association with social capital in rural Fujian communities, China. *Am J Hum Biol* 2018:e23135.

22 Zhao Y, Crimmins E, Hu P, *et al.* CHARLS Blood Sample Users' Guide. National School of Development, Peking University 2014.

23 Pearson TA. Markers of Inflammation and Cardiovascular Disease: Application to Clinical and Public Health Practice: A Statement for Healthcare Professionals From the Centers for Disease Control and Prevention and the American Heart Association. *Circulation* 2003;107:499-511.

24 Kiecolt-Glaser JK, Gouin JP, Hantsoo L. Close relationships, inflammation, and health. *Neurosci Biobehav Rev* 2010;35:33-8.

25 Zheng H, Thomas PA. Marital status, self-rated health, and mortality: overestimation of health or diminishing protection of marriage? *Journal of health and social behavior* 2013;54:128-43.

26 Howell RT, Howell CJ. The relation of economic status to subjective well-being in developing countries: a meta-analysis. *Psychol Bull* 2008;134:536-60.

27 Dowd JB, Zajacova A. Does the predictive power of self-rated health for subsequent mortality risk vary by socioeconomic status in the US? *Int J Epidemiol* 2007;36:1214-21.

28 Huisman M, van Lenthe F, Mackenbach J. The predictive ability of self-assessed health for mortality in different educational groups. *Int J Epidemiol* 2007;36:1207-13.

29 Franks P, Gold MR, Fiscella K. Sociodemographics, self-rated health, and mortality in the US. *Social Science & Medicine* 2003;56:2505-14.

30 Regidor E, Guallar-Castillon P, Gutierrez-Fisac JL, *et al.* Socioeconomic variation in the magnitude of the association between self-rated health and mortality. *Ann Epidemiol* 2010;20:395-400.

For peer review only - http://bmjopen.bmj.com/site/about/guidelines.xhtml

31 Benyamini Y, Leventhal EA, Leventhal H. Gender differences in processing information for making self-assessments of health. *Psychosomatic Medicine* 2000;62:354-64.

32 Benyamini Y. Why does self-rated health predict mortality? An update on current knowledge and a research agenda for psychologists. *Psychol Health* 2011;26:1407-13.

33 Lekander M, Elofsson S, Neve IM*, et al.* Self-rated health is related to levels of circulating cytokines. *Psychosom Med* 2004;66:559-63.

34 Coussons-Read ME, Okun ML, Nettles CD. Psychosocial stress increases inflammatory markers and alters cytokine production across pregnancy. *Brain Behav Immun* 2007;21:343-50.

35 Shivpuri S, Gallo LC, Crouse JR*, et al.* The association between chronic stress type and C-reactive protein in the multi-ethnic study of atherosclerosis: does gender make a difference? *J Behav Med* 2012;35:74-85.

36 Molarius A, Berglund K, Eriksson C*, et al.* Socioeconomic conditions, lifestyle factors, and self-rated health among men and women in Sweden. *European journal of public health* 2007;17:125-33.

37 Gleeson M, Bishop NC, Stensel DJ*, et al.* The anti-inflammatory effects of exercise: mechanisms and implications for the prevention and treatment of disease. *Nat Rev Immunol* 2011;11:607-15.

38 DiMatteo MR, Haskard KB, Williams SL. Health beliefs, disease severity, and patient adherence: a meta-analysis. *Medical care* 2007:521-8.

39 Kronish IM, Rieckmann N, Shimbo D*, et al.* Aspirin adherence, aspirin dosage, and C-reactive protein in the first 3 months after acute coronary syndrome. *Am J Cardiol* 2010;106:1090-4.

For peer review only - http://bmjopen.bmj.com/site/about/guidelines.xhtml

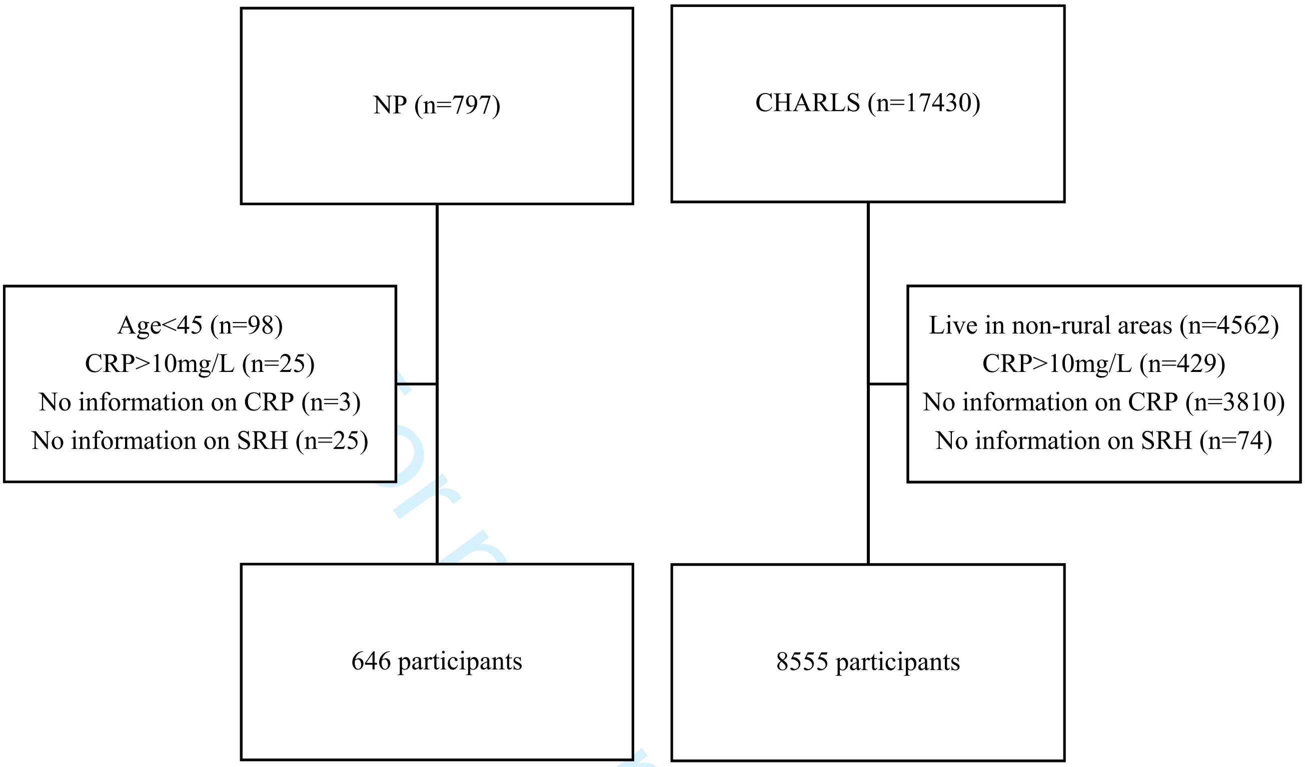

Figure 1 Flowchart of the study populations in NP and CHARLS

For peer review only - http://bmjopen.bmj.com/site/about/guidelines.xhtml

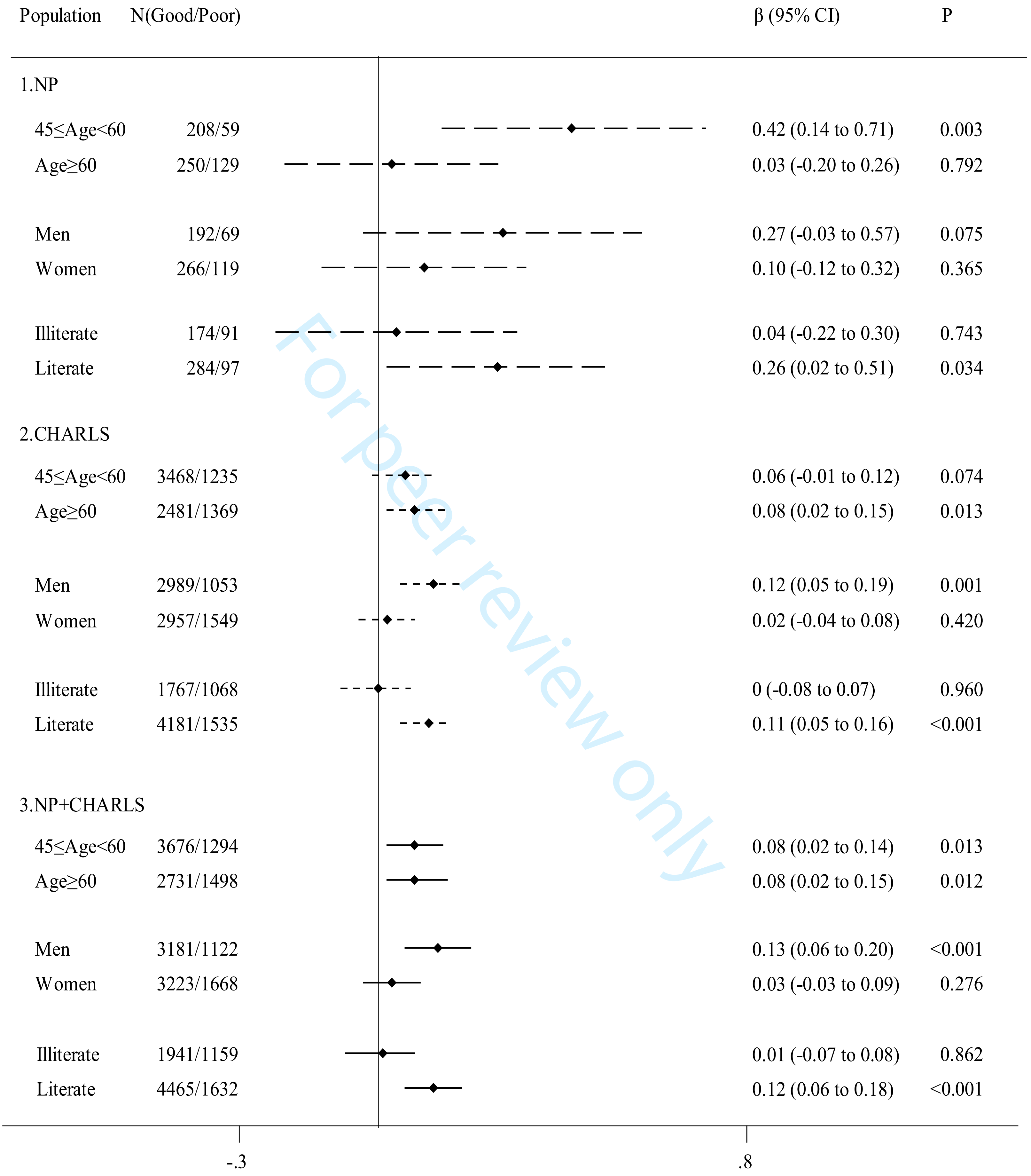

Figure 2 Linear association between poor self-rated health and elevated levels of CRP in NP, CHARLS, and combined populations of NP and CHARLS: stratified by age, sex and education. SRH is dichotomized into two groups (poor to very poor versus good to average). Models are simultaneously adjusted for age, sex, education, marital status, smoking, alcohol consumption, BMI, health status.

For peer review only

SUPPLEMENTARY FILE

Table S1 Association between self-rated health and levels of C-reactive protein: stratified by age, sex and education (combined population, logistic)

	N (Good/Poor)	Self-rated health (Good/Poor)	P
**Total**	6409/2792	1.18 (1.03 to 1.37)	0.020
Age			
45-60	3676/1294	1.22 (0.98 to 1.52)	0.076
≥60	2731/1498	1.17 (0.97 to 1.41)	0.098
Sex			
Men	3181/1122	1.26 (1.02 to 1.56)	0.031
Women	3223/1668	1.12 (0.92 to 1.35)	0.270
Education			
Illiterate	1941/1159	1.12 (0.89 to 1.41)	0.339
Literate	4465/1632	1.23 (1.03 to 1.48)	0.025

Adjusted for age, sex, education, marital status, smoking, alcohol consumption, BMI, health status.

Table S2 Characteristics of the study sample: stratified by age, sex, education

	NP			CHARLS		
	**Illiterate**	**literate**	**P**	**Illiterate**	**literate**	**P**
Age			<0.001			<0.001
45-60	63 (23.8)	204 (53.5)		1182 (41.7)	3519 (61.6)	
≥60	202 (76.2)	177 (46.5)		1652 (58.3)	2196 (38.4)	
Sex			<0.001			<0.001
Men	77 (29.1)	184 (48.3)		638 (22.5)	3402 (59.6)	
Women	188 (70.9)	197 (51.7)		2194 (77.5)	2310 (40.4)	
Self-rated health			0.067			<0.001
Good	74 (27.9)	114 (29.9)		517 (18.2)	1277 (22.3)	
Average	100 (37.7)	170 (44.6)		1250 (44.1)	2904 (50.8)	
Poor	82 (30.9)	83 (21.8)		878 (31)	1278 (22.4)	
Very poor	9 (3.4)	14 (3.7)		190 (6.7)	257 (4.5)	
Health status			0.002			<0.001
Healthy	37 (14)	90 (23.7)		605 (21.5)	1483 (26.2)	
Unhealthy	228 (86)	290 (76.3)		2213 (78.5)	4186 (73.8)	

For peer review only

BMJ Open

The role of education in the association between self-rated health and levels of C-reactive protein: a cross-sectional study in rural areas of China

Journal:	*BMJ Open*
Manuscript ID	bmjopen-2018-027659.R1
Article Type:	Original research
Date Submitted by the Author:	23-Feb-2019
Complete List of Authors:	Tu, Raoping; Nagasaki University Institute of Tropical Medicine, Department of International Health; Nagasaki University Graduate School of Biomedical Sciences, Leading Program<br>Pan, Kuan-Yu; Karolinska Institutet, Stockholm University, Department of Neurobiology, Care Sciences and Society (NVS), Aging Research Center<br>Cai, Guoxi; Nagasaki Prefectural Institute of Environment and Public Health; Nagasaki University Institute of Tropical Medicine, Department of International Health<br>Yamamoto, Taro; Nagasaki University Institute of Tropical Medicine, Department of International Health<br>Wang, Hui-Xin; Stockholm University, Stress Research Institute; Karolinska Institutet, Department of Neurobiology, Care Sciences and Society (NVS), Aging Research Center
**Primary Subject Heading**:	Public health
Secondary Subject Heading:	Epidemiology
Keywords:	EPIDEMIOLOGY, PUBLIC HEALTH, SOCIAL MEDICINE

SCHOLARONE™ Manuscripts

Title page

The role of education in the association between self-rated health and levels of C-reactive protein: a cross-sectional study in rural areas of China

Author's names and affiliation:

Raoping Tu [1,2], Kuan-Yu Pan [3], Guoxi Cai [1,4], Taro Yamamoto [1,*], Hui-Xin Wang [3,5,*]

[1] Department of International Health, Institute of Tropical Medicine (NEKKEN), Nagasaki University, Nagasaki, Japan.

[2] Leading Program, Graduate School of Biomedical Sciences, Nagasaki University, Nagasaki, Japan.

[3] Aging Research Center, Department of Neurobiology, Care Sciences and Society (NVS), Karolinska Institutet, Stockholm University, Stockholm, Sweden.

[4] Nagasaki Prefectural Institute of Environment and Public Health, Nagasaki, Japan.

[5] Stress Research Institute, Stockholm University, Stockholm, Sweden.

*Correspondence to:

1. Department of International Health, Institute of Tropical Medicine (NEKKEN), Nagasaki University, 1-12-4 Sakamoto, Nagasaki 852-8523, Japan

Tel: +81-095-819-7853, Fax: +81-095-819-7869

Taro Yamamoto

E-mail: y-taro@nagasaki-u.ac.jp

For peer review only - http://bmjopen.bmj.com/site/about/guidelines.xhtml

For peer review only

2. Stress Research Institute, Stockholm University, SE-106 91 Stockholm, Sweden

Tel: +46-08-5537-8941

Hui-Xin Wang

E-mail: huixin.wang@su.se

Word count: 2883

For peer review only - http://bmjopen.bmj.com/site/about/guidelines.xhtml

ABSTRACT

Objectives This study aims to examine the association between self-rated health (SRH) and levels of C-reactive protein (CRP) among adults aged 45 to 101 years old in rural areas of China, and to explore the role of education in the association.

Design Cross-sectional study

Setting The study population was derived from two databases in China: Nanping (Nanping project, NP) and the China Health and Retirement Longitudinal Study (CHARLS).

Participants 646 participants from a rural area of NP, and 8555 rural participants from CHARLS.

Methods CRP was measured using a high sensitivity sandwich enzyme immunoassay in NP and immunoturbidimetric assay in CHARLS. SRH was assessed by self-rated health questionnaires and categorized into good and poor. Education was measured by maximum years of schooling and dichotomized into illiterate and literate. Multivariate linear regression models were used to study the associations.

Results Compared to people with good SRH, those with poor SRH had higher levels of CRP in NP (β=0.16, 95%CI -0.02 to 0.34) and in CHARLS (β=0.07, 95%CI 0.02 to 0.11). This was especially in men and literate people after adjusting for potential confounders. Similar findings were observed in the pooled population (β=0.08, 95%CI 0.03 to 0.12), in men (β=0.13, 95%CI 0.06 to 0.20) and in literate people (β=0.12, 95%CI 0.06 to 0.18).

Conclusion Poor SRH may be a predicator of elevated levels of CRP among middle-

For peer review only - http://bmjopen.bmj.com/site/about/guidelines.xhtml

aged and older people in rural areas, especially in men and literate people.

Keywords Self-rated health; C-reactive protein; Education level

Strengths and limitations of this study

- Our study population comes from two databases, including one national representative sample derived from CHARLS, making our results highly generalizable to the national rural population of China.
- CRP is an objective measure performed by health professionals using validated methods, making it more reliable than subjective measures.
- The application of both linear and logistic regressions ensured our confidence in the findings and facilitated the interpretation of the results.
- Cross-sectional study design prevents us from making causal inferences.
- Convenience sampling in the NP study may bias the results towards over-estimation.

Text

INTRODUCTION

C-reactive protein (CRP), a marker of systemic inflammation, has been shown to be involved in crucial pathogenesis in a variety of negative health outcomes, including cardiovascular diseases,[1 2] diabetes,[3] cancer,[4] and cognitive decline.[5] Since the value of CRP in the prediction of prognoses in health outcomes has been recognized, it is important, from a public health perspective, to identify people at risk of elevated CRP in an efficient and simple way.

For peer review only - http://bmjopen.bmj.com/site/about/guidelines.xhtml

It is well known that self-rated health (SRH) can be simply measured through an individual's subjective perception of his own health, thus many health authorities have introduced SRH for surveillance.[6] SRH has been featured as a strong predictor for functional ability,[7] onset of chronic diseases,[8] and mortality.[9 10] The association between SRH and CRP has been tested in previous works, but the results have been inconsistent.[11-14] These discrepancies may be due to differences in characteristics of the study populations. For example, a Japanese study demonstrated an association between poor SRH and elevated CRP value in women, but not in men (age range 40-69).[14] Among hospital-based studies, poor SRH was associated with elevated CRP in female patients (mean age 63.3±8.7/62.5±8.9 in control/intervention group) with coronary heart disease,[12] but not in some patients with breast cancer (mean age 55.2±8.4).[15]

It is noteworthy that studies concerning the association between SRH and CRP were mostly conducted in developed countries where the study populations were relatively well educated.[11-14] It has been shown that people with different education levels have different perceptions of health.[16] This suggests that the association between SRH and CRP may also be different among people with different educational levels.[17] However, to our knowledge, no study has focused on the difference between illiterate and literate people. In China, despite the decrease in illiteracy from 1990 to 2010, there continues to be large differences between urban and rural areas: the rate of illiteracy in rural areas is more than two times that of urban areas.[18] Considering the lack of resources in rural areas, identifying people at risk of negative health outcomes using a simple measure such as SRH is warranted.

In the current study, we use two databases from China: to examine the association between

For peer review only - http://bmjopen.bmj.com/site/about/guidelines.xhtml

SRH and CRP among middle-aged and older people in rural areas, and to explore whether the SRH-CRP association varies across age, sex and education levels.

METHODS

Study population

Nanping project (NP)

NP is a 2015, voluntary participation, cross-sectional study consisting of residents aged 18 years or older from one county of Nanping City in Fujian Province, China. Seven villages were selected based on recommendations from local health workers, since the residents in these areas are known to be highly cooperative.

As showed in Figure 1, a total of 797 people were enrolled. To match with the age range of study population from the CHARLS, we excluded 98 participants under 45 years old. Those with CRP concentrations higher than 6.25 mg/L in dried blood spots (DBS), which is comparable to 10 mg/L at serum level[19] (n=25), were excluded due to potential acute inflammatory conditions. After further excluding people with missing information on CRP values (n=3) and SRH (n=25), 646 people remained in our current study.

China Health and Retirement Longitudinal Study (CHARLS)

The CHARLS is a nationally representative longitudinal study. Eligible people were selected through a multistage probability sampling, and detailed descriptions of sampling method are provided in the users' guide.[20] In this current study, we used data from the baseline survey in 2011 because the CRP data was only available in that year. This is a secondary analysis of the CHARLS public database.

For peer review only - http://bmjopen.bmj.com/site/about/guidelines.xhtml

Overall, 17430 people were examined at baseline (Figure 1). People who lived in communities, or in both villages and communities (n=4562), and had CRP>10mg/L (n=429) were excluded. We further excluded people with missing data on CRP (n=3810) and SRH (n=74). Finally, 8555 (69%) people were included in our analysis.

Self-rated health (SRH)

SRH was assessed by one question: 'In general how would you rate your health?' Response options were 'good', 'average', 'poor', and 'very poor'.

C-reactive protein (CRP)

NP

Finger prick blood samples were collected by health workers using a filter paper, known as DBS. We kept the DBS at room temperature for a few days after being desiccated during the investigation period, then stored them in the Fujian Medical University at –20°. We used high sensitivity sandwich enzyme immunoassay method to measure CRP concentrations by applying monoclonal antibodies.[19] Further details of the protocols have been presented elsewhere.[21]

CHARLS

The venous blood samples were collected by trained staff from local Chinese Center for Disease Control and Prevention (China CDC). Plasma samples were collected and preserved in 0.5 mL cryovial at -20°C, delivered to Beijing CDC within 2 weeks. Plasma CRP was determined by the immunoturbidimetric assay method at Capital Medical University.[22]

Covariates

For peer review only - http://bmjopen.bmj.com/site/about/guidelines.xhtml

In both cohorts, all participants were interviewed face-to-face by trained interviewers using a questionnaire that covers information on age, sex, education, marital status, smoking, alcohol consumption, and health status. Height and weight were measured by interviewers using standard anthropometers.

Education level was determined by maximum years of schooling: 0 year (illiterate), 1-6 years (elementary school), 7-9 years (junior high school), 10-12 years (senior high school), >12 years (college or above). Due to the fact that more than 30% of both the NP and CHARLS samples were illiterate, we dichotomized education into 0 year (illiterate) and >0 year (literate). Age was dichotomized as 45-60 years versus ≥60 years old, and marital status as married versus non-married. BMI was calculated by dividing weight (kg) by height squared (m^2) and categorized as underweight (<18.5), normal weight (18.5-24.99), overweight (25-29.99), and obese (≥30). Smoking was dichotomized into current smokers and non-current smokers (including former smokers). Alcohol consumption was categorized as regular drinkers (more than 3 times per week) and non-regular drinkers.

Health status was measured by asking the participants whether they had any moderate/severe disease symptoms (e.g., fever) in the last month, or used antihypertensive or antidiabetic medications in the NP, and whether they had ever been diagnosed by a doctor with any diseases (e.g., hypertension), or often suffered from any pain currently in CHARLS. People answering positively were categorized as unhealthy, otherwise healthy.

Statistical analysis

First, data from the NP and CHARLS were analyzed separately. We applied one-way ANOVA to examine the differences of CRP in characteristics in each dataset by using F-distribution.

For peer review only - http://bmjopen.bmj.com/site/about/guidelines.xhtml

The CRP variable was log-transformed because it was not normally distributed. The association between SRH and CRP was estimated by β-coefficient and a 95% confidence interval (CI) using linear regression in two datasets. The first estimate was respective; in the second, datasets were pooled. Fixed-effect meta-analysis was used to examine the heterogeneity. Then we re-ran the linear regression using the pooled dataset.

Next, to facilitate the interpretation of the association between SRH and CRP, multivariate logistic analysis was performed to estimate odds ratios (ORs) and 95% CIs in the two datasets separately. We categorized CRP into two levels: low (<3mg/L) and high (≥3mg/L).[23] Using fixed-effect meta-analysis to examine the heterogeneity of two datasets again. Later, logistic regression was conducted in the combined population.

Age, sex and education were introduced into the basic-adjusted model. Further, we additionally adjusted for marital status, smoking, alcohol consumption, BMI, and health status.[24 25] All analyses were repeated in the stratified analyses by age, sex and education.

In addition, we conducted multiple imputation for missing data. For further sensitivity analyses, we performed additional analyses: 1) We adjusted for psychological distress in the NP and depression in CHARLS; 2) We used sampling weights to derive national estimates in CHARLS;[20] 3) We re-ran linear regression after excluding illiterate participants in order to compare with previous studies; 4) Since the social economic status-psychological well-being association was strong in poor areas,[26] we further adjusted for self-rated household income in the NP and self-rated household living standards in CHARLS as their assessment of social economic status were different.

For peer review only - http://bmjopen.bmj.com/site/about/guidelines.xhtml

All statistical analyses were performed with Stata 13.0 (Stata Corp, College Station, TX, USA).

Patient and public involvement

There were no participants involved in the development of this study.

RESULTS

Characteristics of the participants

The CRP levels across different characteristics of participants were compared in each dataset separately. Table 1 shows that in both datasets that older age people, higher BMI's, poorer SRH, or an unhealthy status were more likely to have elevated levels of CRP. The findings were inconsistent with sex, education, marital status, smoking and alcohol consumption in the two datasets. People with missing CRP values in NP and CHARLS were better educated and reported better health status compared to those who remained in the analyses (Supplementary File: Table S1 and Table S2).

For peer review only

Table 1 CRP values across characteristics of the study population

	NP (n=646)		CHARLS (n=8555)	
	**CRP[a]**	**P**	**CRP**	**P**
Age		<0.001		<0.001
45-60	0.6 (0.3 to 1.2)		0.9 (0.5 to 1.7)	
≥60	0.8 (0.4 to 1.8)		1.1 (0.6 to 2.1)	
Sex		0.011		0.003
Men	0.6 (0.3 to 1.3)		1.0 (0.5 to 2.0)	
Women	0.8 (0.4 to 1.7)		0.9 (0.5 to 1.8)	
Education		0.004		0.316
Illiterate	0.9 (0.4 to 1.8)		1.0 (0.5 to 2.0)	
Literate	0.6 (0.3 to 1.3)		0.9 (0.5 to 1.9)	
Marital status		0.495		<0.001
Married	0.7 (0.3 to 1.5)		0.9 (0.5 to 1.9)	
Non-married	0.7 (0.4 to 1.7)		1.1 (0.6 to 2.4)	
Smoking		0.467		0.041
Current smokers	0.6 (0.3 to 1.4)		1.0 (0.5 to 2.0)	
Non-current smokers	0.7 (0.4 to 1.6)		0.9 (0.5 to 1.9)	
Alcohol consumption		0.001		0.635
Regular drinkers	0.5 (0.3 to 1.1)		0.9 (0.5 to 1.9)	
Non-regular drinkers	0.8 (0.4 to 1.6)		1.0 (0.5 to 1.9)	
BMI		<0.001		<0.001
Underweight (<18.5)	0.5 (0.2 to 1.4)		0.8 (0.5 to 1.9)	
Normal weight (18.5-25)	0.6 (0.3 to 1.1)		0.8 (0.5 to 1.7)	
Overweight (25-30)	1.2 (0.6 to 2.3)		1.2 (0.7 to 2.3)	

For peer review only

For peer review only - http://bmjopen.bmj.com/site/about/guidelines.xhtml

Obese (≥30)	1.6 (1.0 to 4.4)		1.9 (0.9 to 3.3)	
Self-rated health		0.071		<0.001
Good	0.6 (0.3 to 1.7)		0.9 (0.5 to 1.8)	
Average	0.7 (0.3 to 1.5)		0.9 (0.5 to 1.8)	
Poor	0.8 (0.4 to 1.5)		1.0 (0.6 to 2.1)	
Very poor	1.0 (0.5 to 2.3)		1.1 (0.6 to 2.3)	
Health status[b]		0.002		<0.001
Healthy	0.5 (0.3 to 1.3)		0.8 (0.5 to 1.7)	
Unhealthy	0.8 (0.4 to 1.6)		1.0 (0.5 to 2.0)	

[a] Median (interquartile range); comparison was done with log-transformed values.

[b] Health status:

Unhealthy: Self-reported moderate to severe symptoms in the last month or used antihypertensive or antidiabetic medications (NP); Had been diagnosed by a doctor with any disease or often suffered from any pain currently (CHARLS). Healthy: no such report.

[c] Missing values:
NP: 1 missing in health status.
CHARLS: 2 missing in age, 7 missing in sex, 4 missing in education,1 missing in smoking, 3 missing in alcohol consumption, 1191 missing in BMI, 65 missing in health status.

For peer review only

SRH and CRP

Table 2 presents the association between SRH and CRP in the two individual populations. In the NP, a borderline statistically significant association was observed between very poor SRH and elevated levels of CRP (β=0.39, 95%CI -0.07 to 0.85) in basic-adjusted model, while the association was attenuated after adjusting for confounders (β=0.29, 95%CI -0.15 to 0.73). Despite insignificance, the estimated effect of SRH started to change direction from average SRH (β = -0.05) to poor SRH (β = 0.10). In CHARLS, poor and very poor SRH were both associated with higher CRP (β=0.06, 95%CI 0 to 0.12; β=0.11, 95%CI 0.01 to 0.22). Considering the same pattern in both two datasets that poor and very poor SRH have similar effect on CRP and so as good and average SRH, and that there are limited number of participants with very poor SRH in NP, we combined 'good' and 'average' as good SRH, 'poor' and 'very poor' as poor SRH. Further, we found that poor SRH was associated with higher levels of CRP both in NP (β=0.16, 95%CI -0.02 to 0.34) and CHARLS (β=0.07, 95%CI 0.02 to 0.11) (Table 2).

Table 2 Association between self-rated health and C-reactive protein

		Model1[a]		Model2[b]	
	N	β (95%CI)	P	β (95%CI)	P
**NP**					
Good	188	Ref.		Ref.	
Average	270	-0.03 (-0.22 to 0.17)	0.792	-0.05 (-0.24 to 0.14)	0.589
Poor	165	0.12 (-0.10 to 0.34)	0.292	0.10 (-0.11 to 0.32)	0.349
Very Poor	23	0.39 (-0.07 to 0.85)	0.093	0.29 (-0.15 to 0.73)	0.202
Good/Poor	458/188	0.17 (-0.01 to 0.35)	0.067	0.16 (-0.02 to 0.34)	0.077
**CHARLS**					
Good	1794	Ref.		Ref.	
Average	4157	0.01 (-0.04 to 0.06)	0.613	0.00 (-0.05 to 0.06)	0.911
Poor	2157	0.10 (0.04 to 0.15)	0.001	0.06 (0.00 to 0.12)	0.055
Very Poor	447	0.16 (0.06 to 0.25)	0.001	0.11 (0.01 to 0.22)	0.036
Good/Poor	5951/2604	0.10 (0.05 to 0.14)	<0.001	0.07 (0.02 to 0.11)	0.004
**NP+CHARLS**					
Good	1982	Ref.		Ref.	
Average	4427	0.02 (-0.03 to 0.07)	0.379	0.01 (-0.04 to 0.06)	0.643

For peer review only - http://bmjopen.bmj.com/site/about/guidelines.xhtml

Poor	2322	0.11 (0.05 to 0.16)	<0.001	0.08 (0.02 to 0.14)	0.013
Very Poor	470	0.18 (0.09 to 0.28)	<0.001	0.14 (0.04 to 0.24)	0.007
Good/Poor	6409/2792	0.11 (0.06 to 0.15)	<0.001	0.08 (0.03 to 0.12)	0.001

[a] Adjusted for age, sex, education

[b] Adjusted for age, sex, education, marital status, smoking, alcohol consumption, BMI, health status

As the same direction of effect of estimate and a very low level of heterogeneity (I-squared< 0.001%) were observed in the two datasets (data not shown), we pooled the data and re-ran the linear regression analyses in the combined populations. The association between poorer SRH and higher CRP was observed in the pooled population (β=0.08, 95%CI 0.03 to 0.12) (Table2).

The roles of age, sex, and education in the association between SRH and CRP

The association between SRH and CRP stratified by age, sex, education is showed in Figure 2. In middle-aged people, worse SRH was associated with higher CRP both in NP (β=0.42, 95%CI 0.14 to 0.71) and CHARLS (β=0.06, 95%CI -0.01 to 0.12). Among older people, a similar trend was observed in CHARLS (β=0.08, 95%CI 0.02 to 0.15), but not in the NP. When stratified by sex, we found a statistically significant SRH-CRP association among men both in NP (β=0.27, 95%CI -0.03 to 0.57) and CHARLS (β=0.12, 95%CI 0.05 to 0.19), but not in women. In a stratified analysis by education, the association between SRH and CRP was seen in literate people both in NP (β=0.26, 95%CI 0.02 to 0.51) and CHARLS (β=0.11, 95%CI 0.05 to 0.16), but not in illiterate people.

In the pooled population, the SRH-CRP association was repeated in the middle-aged (β=0.08, 95%CI 0.02 to 0.14), older people (β=0.08, 95%CI 0.02 to 0.15), men (β=0.13, 95%CI 0.06 to 0.20), and literate people (β=0.12, 95%CI 0.06 to 0.18) (Figure 2).

Furthermore, we applied a logistic regression based on the pooled data. The OR for having

For peer review only - http://bmjopen.bmj.com/site/about/guidelines.xhtml

elevated levels of CRP in those with poor SRH was 1.18 in the total population (95%CI 1.03 to 1.37), 1.26 in men (95%CI 1.02 to 1.56), and 1.23 in the literate people (95%CI 1.03 to 1.48). Similar ORs were observed in the middle-aged and older people (Supplementary File: Table S3).

Additional analysis

Similar results were observed using data from multiple imputations for missing data (Supplementary File: Table S4 and Table S5). and from sensitivity analyses further adjusting for psychological distress in the NP and depression in CHARLS. Identical trends of age and sex differences as main results were observed in literate people in both datasets, but not in the illiterate. Additional adjustment for socio-economic status did not result in any further changes.

DISCUSSION

In this study, based on 9201 residents in rural area, we found that poor SRH is associated with an elevated level of CRP in middle-aged and older people, especially among the literate and men.

Our finding of the association between poorer SRH and higher CRP level is in line with results from previous studies that included participants in similar age as our study participants.[11 14] Yet, those studies mainly looked at people living in industrialized countries with higher education while our participants resided in less developed country with features of low literacy.

Possible pathways linking poor SRH and elevated level of CRP could be due to psychological stress and health behavior. Poor SRH may reflect a poor physical (e.g., inaccessibility to health service) and social environment (e.g., limited social network), which can limit one's coping

For peer review only - http://bmjopen.bmj.com/site/about/guidelines.xhtml

ability and induce psychological stress. It is known that stress can activate the sympathetic nervous system and the hypothalamic-pituitary-adrenal axis, contributing to the production of stress hormones, which in turn increase the secretion of CRP.[27 28] In addition, people with poor SRH were less likely to have an active lifestyle.[29] Having an inactive lifestyle has been suggested to potentially weaken the immune system and facilitate the inflammation processes through the release of pro-inflammatory adipokines.[30] Furthermore, poor SRH may also reflects poor medication adherence,[31] such as low aspirin adherence, which has been associated with elevated levels of CRP in the first 3 months after acute coronary syndrome.[32] By contrary, elevated CRP has been linked to depressive symptom or psychological distress symptom,[33 34] which may also result in poorly rated health status in individuals.

It is notable that poor SRH was associated with an elevated CRP level in literate participants, but not in the illiterate participants, which was consistent with one previous study.[17] Indeed, similar results were also shown in studies focusing on SRH and mortality.[35 36] One of the possible explanations may be that illiterate people are often lack of health-related knowledge and access to health care,[16] and thus may misinterpret the feeling that they have in their bodies.[37] It has been shown that poor SRH in the less educated people mainly represents less serious diseases.[38] In our study, we also found that illiterate people were more likely to rate their health as poor and to report illness or pain both in NP and CHARLS (Supplementary File: Table S6). Moreover, illiterate people may have to withstand more pressure as they have less social and financial resources. Thus, other factors may contribute to the reported poor SRH, rather than actual health condition.

We found that SRH-CRP associations were only observed in men, but not in women, which may be due to the potential sex differences in reporting SRH. Previous studies have shown that

For peer review only - http://bmjopen.bmj.com/site/about/guidelines.xhtml

the poor SRH in women can reflect both serious and non-serious diseases, whereas it tends to reflect serious diseases in men.[39] Broad dimensions of health perceptions may lead to less accurate SRH in women. In addition, the proportion of illiterate people among women is much higher than that among men in both datasets, this may explain the different findings between our study and the Iwate-KENCO study from Japan.[14]

The discrepant findings between two datasets are worthy of discussion. First, the association between poor SRH and elevated CRP values among older people (aged≥60 years) was observed in CHARLS, but not in NP. And in both populations, poor SRH was only associated with higher CRP in men, not in women. These findings may also be explained by educational level in each subgroup. That is, the proportion of illiterate people was relatively higher in older adults in NP (76.2%) than in CHARLS (58.3%) as shown in Table S6 (Supplementary File), and there was a higher proportion of illiterate people in women in both populations. Second, after excluding the illiterate people, we observed similar age and sex differences in the associations between SRH and CRP among the literate, i.e. poor SRH is associated with elevated CRP values among literate people, especially in men, which was the same as the main results. This suggests that education might play a role in the SRH-CRP association. Third, similar results were observed in urban areas of CHARLS, and further adjusting for socioeconomic status (i.e. self-rated household income in NP, self-rated household living standards in CHARLS) did not change the SRH-CRP association (data not shown), suggesting socioeconomic status might not influence the SRH-CRP association.

This study provides evidence that SRH, a simple measure, may be used as an indicator of ill-physical health among middle-aged and older literate people, but not among the illiterate people, in rural area. In China, the implementation of health surveillance is more challenging

For peer review only - http://bmjopen.bmj.com/site/about/guidelines.xhtml

in rural than in urban areas because of the discrepancy in the aging processes,[40] knowledge gaps[18] and income inequality between these two areas. Elevated CRP has been associated with various physical[1-4] and psychological health outcomes[33 34]. Thus, our results support the consideration of using an efficient and cost-effective way, such as SRH, to monitor the health status in rural population where medical resources are limited. Future studies are needed to confirm our results and extend these findings to larger and more diverse populations, or with other health outcomes. Identification of simple health indictors for illiterate people are warranted.

Acknowledgments We would like to express our sincere gratitude to the participants and local staff in NP project. We are grateful for those supporters: Fukui University (Aki Yazawa); National Center for Global Health and Medicine, Japan (Yosuke Inoue); Nagasaki Prefectural Institute of Environment and Public Health (Guoxi Cai); Fujian Medical University (Fei He, Jie Chen); Fujian Provincial Center for Disease Control and Prevention (Meng Huang) during the data collection in NP Project. Data from China Health and Retirement Longitudinal Study (CHARLS) were collected by the National School of Development at Peking University, China. We appreciated to University of Copenhagen (Tianwei Xu); Fujian Provincial Center for Disease Control and Prevention (Xiuquan Lin); Nagasaki University (Sabin Nundu) for providing valuable comments in analysis and interpretation of data.

Contributors RT, HW and KP conceptualized the study. RT analyzed the data and drafted the manuscript. HW, KP, GC, TY contributed to critical revisions of the manuscript. RT and HW are responsible for ensuring the integrity and accuracy of the study. All authors have read and approved the final manuscript.

For peer review only - http://bmjopen.bmj.com/site/about/guidelines.xhtml

Funding This study is financed by the Program for Nurturing Global Leaders in Tropical and Emerging Communicable Diseases, Graduate School of Biomedical Sciences, Nagasaki University, Japan (Raoping Tu); the Swedish Research Council (grant number: 2018-02998, Hui-Xin Wang); the Ministry of Education of Taiwan, the Swedish National Graduate School on Ageing and Health (SWEA), and Gamla Tjänarinnor Foundation (Kuan-Yu Pan). NP Project was financially supported by the JSPS KAKENHI from the Japan Society for the Promotion of Science (13J06172).

Competing Interests None declared.

Patient consent Not required.

Ethics approval The Ethics Committee for Medical Research at the University of Tokyo (No. 10515-(1)) and the Ethics Committee of the Institute of Tropical Medicine at Nagasaki University (No. 120910100-5) approved the study protocol of NP. The Medical Ethics Committee of Peking University approved the research protocol of CHARLS.

Provenance and peer review Not commissioned; externally peer reviewed

Data sharing statement All of the CHARLS data will be accessible to researchers around the world at the CHARLS project website (http://charls.pku.edu.cn/en). No additional data available.

For peer review only - http://bmjopen.bmj.com/site/about/guidelines.xhtml

Figure legends

Figure 1 Flowchart of the study populations in NP and CHARLS

Figure 2 β-coefficient and 95% confidence interval (CI) of CRP in relation to poor self-rated health from linear regression models stratified by age, sex and education in NP, CHARLS, and the pooled populations of the two datasets. SRH is dichotomized as poor to very poor versus good to average. Models are simultaneously adjusted for age, sex, education, marital status, smoking, alcohol consumption, BMI, health status.

SUPPLEMENTARY FILE

Table S1 Characteristics of study sample in NP without and with missing values in CRP

Table S2 Characteristics of study sample in CHARLS without and with missing values in CRP

Table S3 Odds ratio and 95% confidence interval (95% CI) between poor self-rated health and levels of C-reactive protein: stratified by age, sex and education (pooled population, logistic)

Table S4 Association between self-rated health and C-reactive protein (After multiple imputation)

Table S5 Association between self-rated health and C-reactive protein: stratified by age, sex and education (After multiple imputation)

Table S6 Characteristics of the study samples: stratified by datasets and education

For peer review only - http://bmjopen.bmj.com/site/about/guidelines.xhtml

References

1 Black PH, Garbutt LD. Stress, inflammation and cardiovascular disease. *Journal of psychosomatic research* 2002;52:1-23.

2 Ridker PM. From C-Reactive Protein to Interleukin-6 to Interleukin-1. *Circulation Research* 2016;118:145-56.

3 Barzilaym JI, Freedland ES. Inflammation and its relationship to insulin resistance, type 2 diabetes mellitus, and endothelial dysfunction. *Metabolic syndrome and related disorders* 2003;1:55-67.

4 Wang J, Lee IM, Tworoger SS, *et al*. Plasma C-reactive protein and risk of breast cancer in two prospective studies and a meta-analysis. *Cancer Epidemiol Biomarkers Prev* 2015;24:1199-206.

5 Panza F, Solfrizzi V, Logroscino G, *et al*. Current epidemiological approaches to the metabolic-cognitive syndrome. *J Alzheimers Dis* 2012;30 Suppl 2:S31-75.

6 Salomon JA, Nordhagen S, Oza S, *et al.* Are Americans feeling less healthy? The puzzle of trends in self-rated health. *Am J Epidemiol* 2009;170:343-51.

7 Idler EL, Kasl SV. Self-ratings of health: do they also predict change in functional ability? *J Gerontol B Psychol Sci Soc Sci* 1995;50:S344-53.

8 Latham K, Peek CW. Self-Rated Health and Morbidity Onset Among Late Midlife U.S. Adults. *The Journals of Gerontology Series B: Psychological Sciences and Social Sciences* 2012;68:107-16.

9 DeSalvo KB, Bloser N, Reynolds K, *et al*. Mortality prediction with a single general self-rated health question. A meta-analysis. *J Gen Intern Med* 2006;21:267-75.

10 Singh-Manoux A, Dugravot A, Shipley MJ, *et al*. The association between self-rated health and mortality in different socioeconomic groups in the GAZEL cohort study. *Int J Epidemiol* 2007;36:1222-8.

For peer review only - http://bmjopen.bmj.com/site/about/guidelines.xhtml

11 Christian LM, Glaser R, Porter K, *et al*. Poorer self-rated health is associated with elevated inflammatory markers among older adults. *Psychoneuroendocrinology* 2011;36:1495-504.

12 Janszky I, Lekander M, Blom M, *et al*. Self-rated health and vital exhaustion, but not depression, is related to inflammation in women with coronary heart disease. *Brain Behav Immun* 2005;19:555-63.

13 Shanahan L, Bauldry S, Freeman J, *et al*. Self-rated health and C-reactive protein in young adults. *Brain, Behavior, and Immunity* 2014;36:139-46.

14 Tanno K, Ohsawa M, Onoda T, *et al*. Poor self-rated health is significantly associated with elevated C-reactive protein levels in women, but not in men, in the Japanese general population. *Journal of Psychosomatic Research* 2012;73:225-31.

15 Orre IJ, Reinertsen KV, Aukrust P, *et al*. Higher levels of fatigue are associated with higher CRP levels in disease-free breast cancer survivors. *J Psychosom Res* 2011;71:136-41.

16 Spuling SM, Huxhold O, Wurm S. Predictors of Self-Rated Health: Does Education Play a Role Above and Beyond Age? *J Gerontol B Psychol Sci Soc Sci* 2017;72:415-24.

17 Dowd JB, Zajacova A. Does self-rated health mean the same thing across socioeconomic groups? Evidence from biomarker data. *Ann Epidemiol* 2010;20:743-9.

18 Guo S, Guo Y. *Spotlight on China: Changes in education under China's market economy*: Springer, 2016.

19 Brindle E, Fujita M, Shofer J, *et al*. Serum, plasma, and dried blood spot high-sensitivity C-reactive protein enzyme immunoassay for population research. *J Immunol Methods* 2010;362:112-20.

20 Zhao Y, Strauss J, Yang G, *et al. China health and retirement longitudinal study–2011–2012 national baseline users' guide*. National School of Development, Peking University, 2013.

21 Yazawa A, Inoue Y, Cai G, *et al*. Epstein-Barr virus antibody titer as a stress biomarker

For peer review only - http://bmjopen.bmj.com/site/about/guidelines.xhtml

and its association with social capital in rural Fujian communities, China. *Am J Hum Biol* 2018:e23135.

22 Zhao Y, Crimmins E, Hu P, *et al*. *CHARLS Blood Sample Users' Guide*. National School of Development, Peking University, 2014.

23 Pearson TA. Markers of Inflammation and Cardiovascular Disease: Application to Clinical and Public Health Practice: A Statement for Healthcare Professionals From the Centers for Disease Control and Prevention and the American Heart Association. *Circulation* 2003;107:499-511.

24 Kiecolt-Glaser JK, Gouin JP, Hantsoo L. Close relationships, inflammation, and health. *Neurosci Biobehav Rev* 2010;35:33-8.

25 Zheng H, Thomas PA. Marital status, self-rated health, and mortality: overestimation of health or diminishing protection of marriage? *Journal of health and social behavior* 2013;54:128-43.

26 Howell RT, Howell CJ. The relation of economic status to subjective well-being in developing countries: a meta-analysis. *Psychol Bull* 2008;134:536-60.

27 Coussons-Read ME, Okun ML, Nettles CD. Psychosocial stress increases inflammatory markers and alters cytokine production across pregnancy. *Brain Behav Immun* 2007;21:343-50.

28 Shivpuri S, Gallo LC, Crouse JR, *et al.* The association between chronic stress type and C-reactive protein in the multi-ethnic study of atherosclerosis: does gender make a difference? *J Behav Med* 2012;35:74-85.

29 Molarius A, Berglund K, Eriksson C, *et al*. Socioeconomic conditions, lifestyle factors, and self-rated health among men and women in Sweden. *European journal of public health* 2007;17:125-33.

30 Gleeson M, Bishop NC, Stensel DJ, *et al*. The anti-inflammatory effects of exercise:

For peer review only

For peer review only - http://bmjopen.bmj.com/site/about/guidelines.xhtml

mechanisms and implications for the prevention and treatment of disease. *Nat Rev Immunol* 2011;11:607-15.

31 DiMatteo MR, Haskard KB, Williams SL. Health beliefs, disease severity, and patient adherence: a meta-analysis. *Medical care* 2007:521-8.

32 Kronish IM, Rieckmann N, Shimbo D, *et al*. Aspirin adherence, aspirin dosage, and C-reactive protein in the first 3 months after acute coronary syndrome. *Am J Cardiol* 2010;106:1090-4.

33 Valkanova V, Ebmeier KP, Allan CL. CRP, IL-6 and depression: a systematic review and meta-analysis of longitudinal studies. *J Affect Disord* 2013;150(3):736-44.

34 Wium-Andersen MK, Orsted DD, Nielsen SF, *et al.* Elevated C-reactive protein levels, psychological distress, and depression in 73, 131 individuals. *JAMA Psychiatry* 2013;70(2):176-84.

35 Dowd JB, Zajacova A. Does the predictive power of self-rated health for subsequent mortality risk vary by socioeconomic status in the US? *Int J Epidemiol* 2007;36:1214-21.

36 Huisman M, van Lenthe F, Mackenbach J. The predictive ability of self-assessed health for mortality in different educational groups. *Int J Epidemiol* 2007;36:1207-13.

37 Franks P, Gold MR, Fiscella K. Sociodemographics, self-rated health, and mortality in the US. *Social Science & Medicine* 2003;56:2505-14.

38 Regidor E, Guallar-Castillon P, Gutierrez-Fisac JL, *et al.* Socioeconomic variation in the magnitude of the association between self-rated health and mortality. *Ann Epidemiol* 2010;20:395-400.

39 Benyamini Y, Leventhal EA, Leventhal H. Gender differences in processing information for making self-assessments of health. *Psychosomatic Medicine* 2000;62:354-64.

40 Gong P, Liang S, Carlton EJ, *et al.* Urbanisation and health in China. *The Lancet* 2012;379(9818):843-52.

For peer review only - http://bmjopen.bmj.com/site/about/guidelines.xhtml

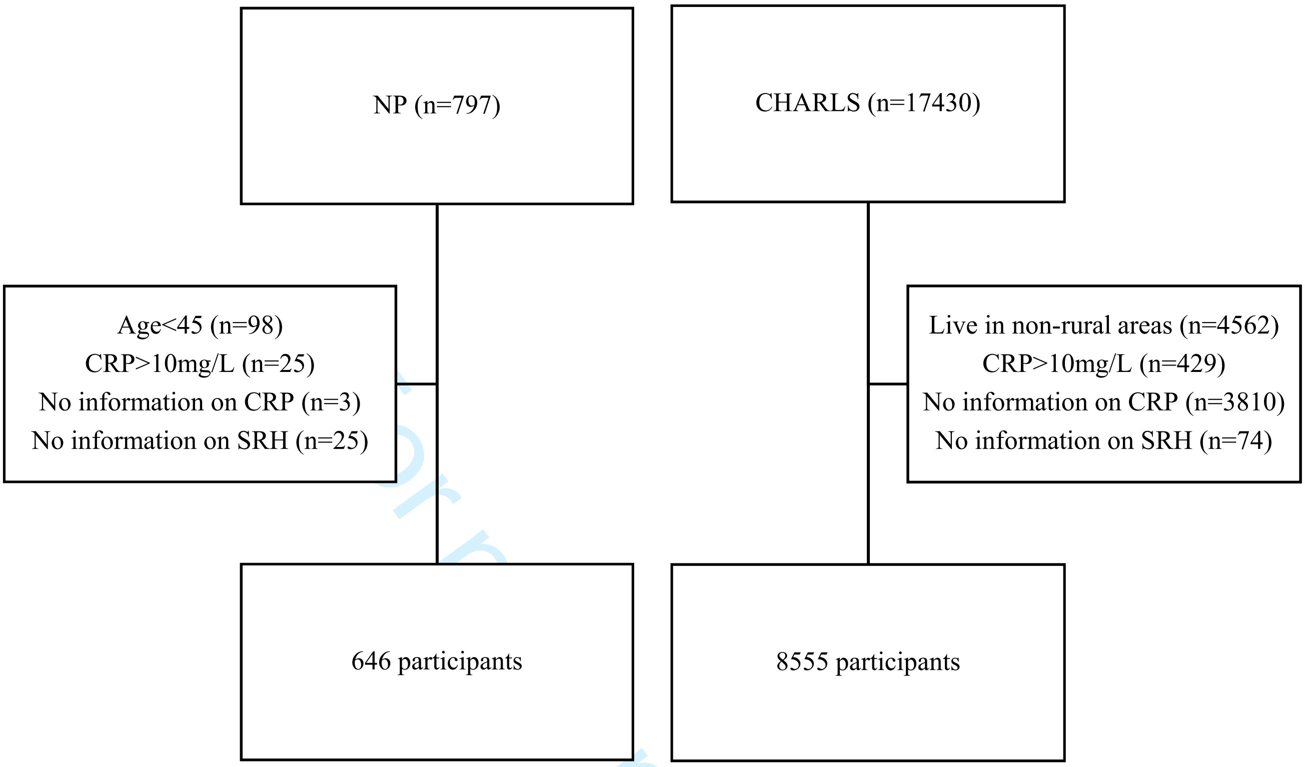

Figure 1 Flowchart of the study populations in NP and CHARLS

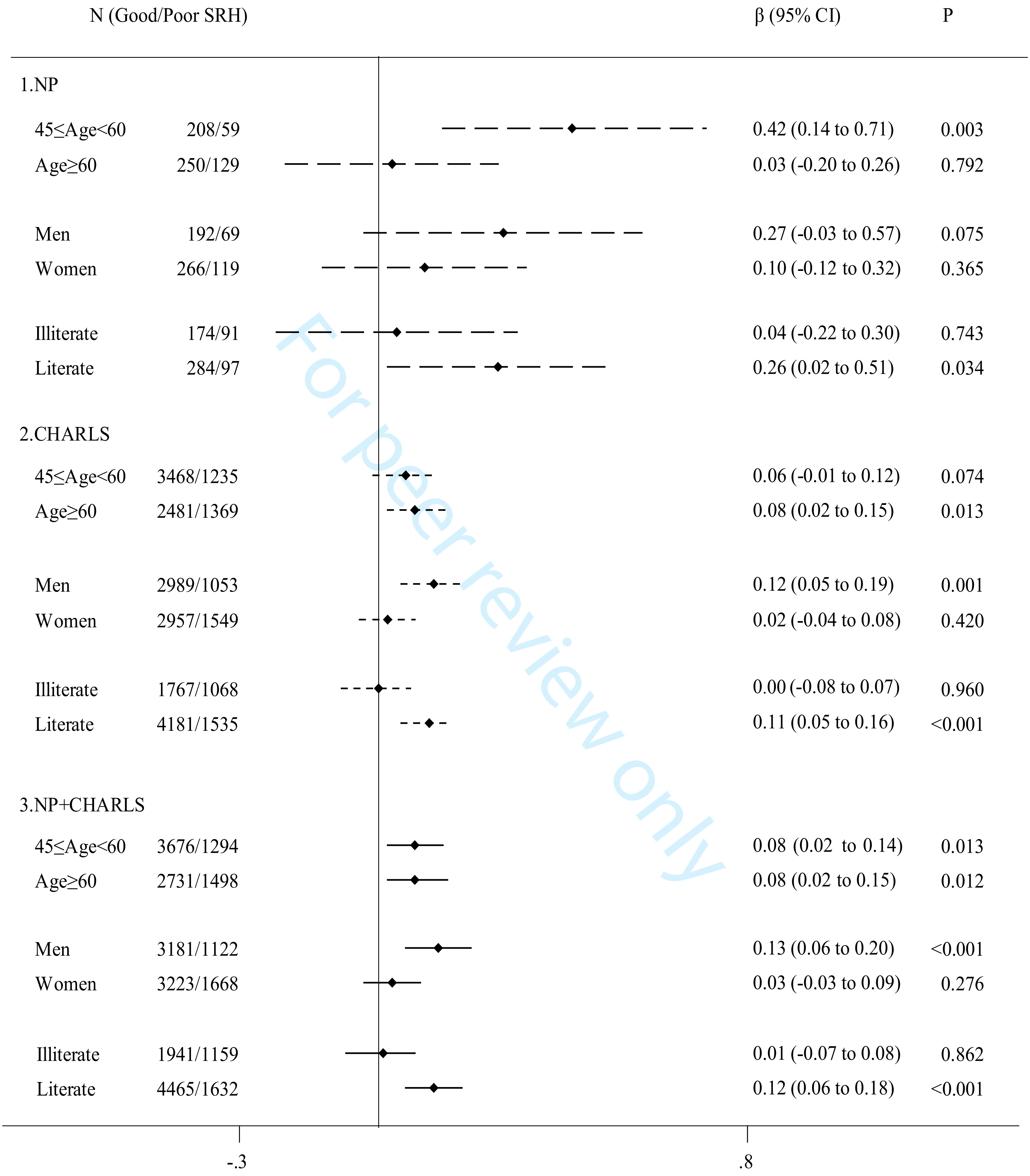

Figure 2 β-coefficient and 95% confidence interval (CI) of CRP in relation to poor self-rated health from linear regression models stratified by age, sex and education in NP, CHARLS, and the pooled populations of the two datasets. SRH is dichotomized as poor to very poor versus good to average. Models are simultaneously adjusted for age, sex, education, marital status, smoking, alcohol consumption, BMI, health status.

For peer review only

SUPPLEMENTARY FILE

Table S1 Characteristics of study sample in NP without and with missing values in CRP

	Non-missing[a] (n=646)	Missing[a] (n=2)	P
Age			0.093
<60	267(41.3)	2(100)	
≥60	379(58.7)	0(0)	
Sex			0.245
Men	261(40.4)	0(0)	
Women	385(59.6)	2(100)	
Education			0.239
Illiterate	265(41.0)	0(0)	
Literate	381(59.0)	2(100)	
Marital status			0.491
Married	522(80.8)	2(100)	
Non-married	124(19.2)	0(0)	
Smoking			0.582
Current smokers	85(13.2)	0(0)	
Non-current smokers	561(86.8)	2(100)	
Drinking			0.489
Regular drinkers	125(19.4)	0(0)	
Non-regular drinkers	521(80.7)	2(100)	
BMI			0.810
Underweight (<18.5)	30(4.60)	0(0)	
Normal weight (18.5-25)	436(67.5)	2(100)	
Overweight (25-30)	158(24.5)	0(0)	
Obese (≥30)	22(3.4)	0(0)	
Self-rated health			0.184
Good	188(29.1)	2(100)	
Average	270(41.8)	0(0)	
Poor	165(25.5)	0(0)	
Very poor	23(3.6)	0(0)	
Health status[b]			0.018
Healthy	127(19.7)	2(100)	
Unhealthy	518(80.2)	0(0)	
Missing	1(0.2)	0(0)	

[a] Data are presented as n (%)

[b] Healthy status:

Unhealthy: Self-reported moderate to severe symptoms in the last month or used antihypertensive or antidiabetic medications (NP).

Healthy: No such report.

For peer review only

Table S2 Characteristics of study sample in CHARLS without and with missing values in CRP

	Non-missing[a] (n=8555)	Missing[a] (n=3810)	P
Age			0.002
<60	4703(55.0)	2226(58.4)	
≥60	3850(45.0)	1583(41.6)	
Missing	2(0)	1(0)	
Sex			<0.001
Men	4042(47.3)	2014(52.9)	
Women	4506(52.7)	1794(47.1)	
Missing	7(0.1)	2(0.1)	
Education			0.001
Illiterate	2835(33.1)	1160(30.5)	
Literate	5716(66.8)	2643(69.4)	
Missing	4(0.1)	7(0.2)	
Marital status			0.001
Married	7517(87.9)	3263(85.6)	
Non-married	1038(12.1)	547(14.4)	
Smoking			0.113
Current smokers	2561(29.9)	1086(28.5)	
Non-current smokers	5993(70.1)	2722(71.4)	
Missing	1(0)	2(0.1)	
Drinking			0.024
Regular drinkers	998(11.7)	399(10.5)	
Non-regular drinkers	7554(88.3)	3406(89.4)	
Missing	3(0)	5(0.1)	
BMI			<0.001
Underweight (<18.5)	535(6.3)	206(5.4)	
Normal weight (18.5-25)	4719(55.2)	1790(47.0)	
Overweight (25-30)	1819(21.3)	592(15.5)	
Obese (≥30)	291(3.4)	101(2.7)	
Missing	1191(13.9)	1121(29.4)	
Self-rated health			0.002
Good	1794(21.0)	910(23.9)	
Average	4157(48.6)	1798(47.2)	
Poor	2157(25.2)	894(23.5)	
Very poor	447(5.2)	208(5.5)	
Health status[b]			<0.001
Healthy	2089(24.4)	1160(30.5)	
Unhealthy	6401(74.8)	2607(68.4)	
Missing	65(0.8)	43(1.1)	

[a] Data are presented as n (%)
[b] Healthy status:
Unhealthy: Had been diagnosed by a doctor with any disease or often suffered from any pain currently (CHARLS).
Healthy: No such report.

For peer review only

Table S3 Odds ratio and 95% confidence interval (95% CI) between poor self-rated health and levels of C-reactive protein: stratified by age, sex and education (pooled population, logistic)

	N (Good/Poor)	OR (95%CI)[a]	P
**Total**	6409/2792	1.18 (1.03 to 1.37)	0.020
Age			
45-60	3676/1294	1.22 (0.98 to 1.52)	0.076
≥60	2731/1498	1.17 (0.97 to 1.41)	0.098
Sex			
Men	3181/1122	1.26 (1.02 to 1.56)	0.031
Women	3223/1668	1.12 (0.92 to 1.35)	0.270
Education			
Illiterate	1941/1159	1.12 (0.89 to 1.41)	0.339
Literate	4465/1632	1.23 (1.03 to 1.48)	0.025

[a] Adjusted for age, sex, education, marital status, smoking, alcohol consumption, BMI, health status.

Table S4 Association between self-rated health and C-reactive protein (After multiple imputation)

		Model1[a]		Model2[b]	
	N	β (95%CI)	P	β (95%CI)	P
**NP**					
Good	190	Ref.		Ref.	
Average	270	-0.02 (-0.22 to 0.18)	0.825	-0.05 (-0.24 to 0.14)	0.602
Poor	165	0.12 (-0.10 to 0.35)	0.275	0.11 (-0.11 to 0.32)	0.341
Very Poor	23	0.40 (-0.06 to 0.86)	0.089	0.29 (-0.15 to 0.73)	0.200
Good/Poor	460/188	0.17 (-0.01 to 0.35)	0.064	0.16 (-0.02 to 0.34)	0.076
**CHARLS**					
Good	2704	Ref.		Ref.	
Average	5955	0.01 (-0.04 to 0.06)	0.717	0.00 (-0.05 to 0.05)	0.962
Poor	3051	0.07 (0.01 to 0.14)	0.016	0.06 (0.00 to 0.12)	0.052
Very Poor	655	0.11 (0.02 to 0.21)	0.023	0.10 (0.00 to 0.20)	0.041
Good/Poor	8659/3706	0.08 (0.03 to 0.12)	0.002	0.07 (0.02 to 0.12)	0.009
**NP+CHARLS**					
Good	2894	Ref.		Ref.	
Average	6225	0.02 (-0.02 to 0.06)	0.349	0.01 (-0.03 to 0.05)	0.639
Poor	3216	0.08 (0.03 to 0.13)	0.002	0.07 (0.02 to 0.11)	0.009
Very Poor	678	0.13 (0.04 to 0.22)	0.005	0.12 (0.03 to 0.21)	0.012
Good/Poor	9119/3894	0.07 (0.03 to 0.11)	<0.001	0.07 (0.03 to 0.11)	0.001

[a] Adjusted for age, sex, education

[b] Adjusted for age, sex, education, marital status, smoking, alcohol consumption, BMI, health status

For peer review only

Table S5 Association between self-rated health and C-reactive protein: stratified by age, sex and education (After multiple imputation)

	N (Good/Poor)	β (95%CI)[a]	P
**NP**			
Age			
45-60	210/59	0.42 (0.14 to 0.71)	0.003
≥60	250/129	0.03 (-0.20 to 0.26)	0.792
Sex			
Men	192/69	0.27 (-0.03 to 0.57)	0.075
Women	268/119	0.10 (-0.12 to 0.32)	0.370
Education			
Illiterate	174/91	0.04 (-0.22 to 0.30)	0.743
Literate	286/97	0.26 (0.02 to 0.51)	0.034
**CHARLS**			
Age			
45-60	5183/1746	0.08 (0.02 to 0.13)	0.007
≥60	3473/1960	0.06 (0.00 to 0.12)	0.064
Sex			
Men	4505/1551	0.09 (0.02 to 0.17)	0.019
Women	4148/2152	0.03 (-0.03 to 0.08)	0.310
Education			
Illiterate	2485/1510	0.02 (-0.04 to 0.08)	0.504
Literate	6167/2192	0.08 (0.02 to 0.13)	0.006
**NP+CHARLS**			
Age			
45-60	5393/1805	0.09 (0.03 to 0.14)	0.002
≥60	3723/2089	0.06 (0.00 to 0.13)	0.040
Sex			
Men	4697/1620	0.11 (0.03 to 0.18)	0.007
Women	4416/2271	0.03 (-0.02 to 0.08)	0.213
Education			
Illiterate	2659/1601	0.03 (-0.03 to 0.09)	0.377
Literate	6453/2289	0.09 (0.03 to 0.14)	0.002

[a] Adjusted for age, sex, education, marital status, smoking, alcohol consumption, BMI, health status

For peer review only

Table S6 Characteristics of the study samples: stratified by datasets and education

	NP			CHARLS		
	**Illiterate**	**literate**	**P**	**Illiterate**	**literate**	**P**
Age			<0.001			<0.001
45-60	63 (23.8)	204 (53.5)		1182 (41.7)	3519 (61.6)	
≥60	202 (76.2)	177 (46.5)		1652 (58.3)	2196 (38.4)	
Sex			<0.001			<0.001
Men	77 (29.1)	184 (48.3)		638 (22.5)	3402 (59.6)	
Women	188 (70.9)	197 (51.7)		2194 (77.5)	2310 (40.4)	
Self-rated health			0.067			<0.001
Good	74 (27.9)	114 (29.9)		517 (18.2)	1277 (22.3)	
Average	100 (37.7)	170 (44.6)		1250 (44.1)	2904 (50.8)	
Poor	82 (30.9)	83 (21.8)		878 (31)	1278 (22.4)	
Very poor	9 (3.4)	14 (3.7)		190 (6.7)	257 (4.5)	
Health status			0.002			<0.001
Healthy	37 (14)	90 (23.7)		605 (21.5)	1483 (26.2)	
Unhealthy	228 (86)	290 (76.3)		2213 (78.5)	4186 (73.8)	

For peer review only

STROBE 2007 (v4) Statement—Checklist of items that should be included in reports of *cross-sectional studies*

Section/Topic	Item #	Recommendation	Reported on page #
**Title and abstract**	1	(*a*) Indicate the study's design with a commonly used term in the title or the abstract	P1
		(*b*) Provide in the abstract an informative and balanced summary of what was done and what was found	P3
**Introduction**			
Background/rationale	2	Explain the scientific background and rationale for the investigation being reported	P4-5
Objectives	3	State specific objectives, including any prespecified hypotheses	P5-6
**Methods**			
Study design	4	Present key elements of study design early in the paper	P6
Setting	5	Describe the setting, locations, and relevant dates, including periods of recruitment, exposure, follow-up, and data collection	P6-8
Participants	6	(*a*) Give the eligibility criteria, and the sources and methods of selection of participants	P6-7
Variables	7	Clearly define all outcomes, exposures, predictors, potential confounders, and effect modifiers. Give diagnostic criteria, if applicable	P7-8
Data sources/ measurement	8*	For each variable of interest, give sources of data and details of methods of assessment (measurement). Describe comparability of assessment methods if there is more than one group	P7-8
Bias	9	Describe any efforts to address potential sources of bias	P9
Study size	10	Explain how the study size was arrived at	P6-7
Quantitative variables	11	Explain how quantitative variables were handled in the analyses. If applicable, describe which groupings were chosen and why	P8-9
Statistical methods	12	(*a*) Describe all statistical methods, including those used to control for confounding	P8-9
		(*b*) Describe any methods used to examine subgroups and interactions	P8-9
		(*c*) Explain how missing data were addressed	P9
		(*d*) If applicable, describe analytical methods taking account of sampling strategy	P9
		(*e*) Describe any sensitivity analyses	P9
**Results**			

For peer review only

Participants	13*	(a) Report numbers of individuals at each stage of study—eg numbers potentially eligible, examined for eligibility, confirmed eligible, included in the study, completing follow-up, and analysed	Figure 1
		(b) Give reasons for non-participation at each stage	Figure 1
		(c) Consider use of a flow diagram	Figure 1
Descriptive data	14*	(a) Give characteristics of study participants (eg demographic, clinical, social) and information on exposures and potential confounders	P10-12
		(b) Indicate number of participants with missing data for each variable of interest	P12
Outcome data	15*	Report numbers of outcome events or summary measures	P11-12
Main results	16	(*a*) Give unadjusted estimates and, if applicable, confounder-adjusted estimates and their precision (eg, 95% confidence interval). Make clear which confounders were adjusted for and why they were included	P13-14, why: P9
		(*b*) Report category boundaries when continuous variables were categorized	P8-9
		(*c*) If relevant, consider translating estimates of relative risk into absolute risk for a meaningful time period	N/A
Other analyses	17	Report other analyses done—eg analyses of subgroups and interactions, and sensitivity analyses	P15
**Discussion**			
Key results	18	Summarise key results with reference to study objectives	P15
Limitations	19	Discuss limitations of the study, taking into account sources of potential bias or imprecision. Discuss both direction and magnitude of any potential bias	P4
Interpretation	20	Give a cautious overall interpretation of results considering objectives, limitations, multiplicity of analyses, results from similar studies, and other relevant evidence	P15-17
Generalisability	21	Discuss the generalisability (external validity) of the study results	P17-18
**Other information**			
Funding	22	Give the source of funding and the role of the funders for the present study and, if applicable, for the original study on which the present article is based	P19

*Give information separately for cases and controls in case-control studies and, if applicable, for exposed and unexposed groups in cohort and cross-sectional studies.

Note: An Explanation and Elaboration article discusses each checklist item and gives methodological background and published examples of transparent reporting. The STROBE checklist is best used in conjunction with this article (freely available on the Web sites of PLoS Medicine at http://www.plosmedicine.org/, Annals of Internal Medicine at http://www.annals.org/, and Epidemiology at http://www.epidem.com/). Information on the STROBE Initiative is available at www.strobe-statement.org.

For peer review only

BMJ Open

The role of education in the association between self-rated health and levels of C-reactive protein: a cross-sectional study in rural areas of China

Journal:	*BMJ Open*
Manuscript ID	bmjopen-2018-027659.R2
Article Type:	Original research
Date Submitted by the Author:	31-Aug-2019

Complete List of Authors:	Tu, Raoping; School of Nursing, Yangzhou University; Nagasaki University Institute of Tropical Medicine, Department of International Health<br>Pan, Kuan-Yu; Karolinska Institutet, Stockholm University, Department of Neurobiology, Care Sciences and Society (NVS), Aging Research Center<br>Cai, Guoxi; Nagasaki Prefectural Institute of Environment and Public Health; Nagasaki University Institute of Tropical Medicine, Department of International Health<br>Yamamoto, Taro; Nagasaki University Institute of Tropical Medicine, Department of International Health<br>Wang, Hui-Xin; Stockholm University, Stress Research Institute; Karolinska Institutet, Department of Neurobiology, Care Sciences and Society (NVS), Aging Research Center

**Primary Subject Heading**:	Public health
Secondary Subject Heading:	Epidemiology
Keywords:	EPIDEMIOLOGY, PUBLIC HEALTH, SOCIAL MEDICINE

SCHOLARONE™ Manuscripts

Title page

The role of education in the association between self-rated health and levels of C-reactive protein: a cross-sectional study in rural areas of China

Author's names and affiliation:

Raoping Tu[1,2,3], Kuan-Yu Pan[4], Guoxi Cai [2,5], Taro Yamamoto [2,*], Hui-Xin Wang [4,6,*]

[1]School of Nursing, Yangzhou University, Yangzhou, China

[2]Department of International Health, Institute of Tropical Medicine (NEKKEN), Nagasaki University, Nagasaki, Japan.

[3] Leading Program, Graduate School of Biomedical Sciences, Nagasaki University, Nagasaki, Japan.

[4] Aging Research Center, Department of Neurobiology, Care Sciences and Society (NVS), Karolinska Institutet, Stockholm University, Stockholm, Sweden.

[5]Nagasaki Prefectural Institute of Environment and Public Health, Nagasaki, Japan.

[6]Stress Research Institute, Stockholm University, Stockholm, Sweden.

*Correspondence to:

1. Department of International Health, Institute of Tropical Medicine (NEKKEN), Nagasaki University, 1-12-4 Sakamoto, Nagasaki 852-8523, Japan

Tel: +81-095-819-7853, Fax: +81-095-819-7869

Taro Yamamoto

For peer review only - http://bmjopen.bmj.com/site/about/guidelines.xhtml

E-mail: y-taro@nagasaki-u.ac.jp

2. Stress Research Institute, Stockholm University, SE-106 91 Stockholm, Sweden

Tel: +46-08-5537-8941

Hui-Xin Wang

E-mail: huixin.wang@su.se

Word count: 2808

For peer review only - http://bmjopen.bmj.com/site/about/guidelines.xhtml

ABSTRACT

Objectives This study aims to examine the association between self-rated health (SRH) and levels of C-reactive protein (CRP) among adults aged 45 to 101 years old in rural areas of China, and to explore the role of education in the association.

Design Cross-sectional study

Setting The study population was derived from two databases in China: Nanping project (NP) and the China Health and Retirement Longitudinal Study (CHARLS).

Participants 646 participants from a rural area of Nanping (NP) and 8555 rural participants from a national representative sample of China (CHARLS).

Methods CRP was measured using a high sensitivity sandwich enzyme immunoassay in the NP and immunoturbidimetric assay in the CHARLS. SRH was assessed by self-rated health questionnaires and categorized into good and poor. Education was measured by the maximum years of schooling and dichotomized into illiterate and literate. Multivariate linear regression models were used to study the associations.

Results Compared to people with good SRH, those with poor SRH had higher levels of CRP in NP (β=0.16, 95% CI -0.02 to 0.34) and in CHARLS (β=0.07, 95% CI 0.02 to 0.11). This was especially in men and literate people after adjusting for potential confounders. Similar findings were observed in the pooled population (β=0.08, 95% CI 0.03 to 0.12), in men (β=0.13, 95% CI 0.06 to 0.20), and in literate people (β=0.12, 95% CI 0.06 to 0.18).

Conclusion Poor SRH may be a predicator of elevated levels of CRP among middle-aged and older people in rural areas, especially in men and literate people.

For peer review only - http://bmjopen.bmj.com/site/about/guidelines.xhtml

Keywords Self-rated health; C-reactive protein; Education level

Strengths and limitations of this study

- Our study population came from two databases, including one national representative sample derived from the China Health and Retirement Longitudinal Study (CHARLS), making our results highly generalizable to the national rural population of China.
- C-reactive protein (CRP) was an objective measure performed by health professionals using validated methods, making it more reliable than subjective measures.
- Cross-sectional study design prevented us from making causal inferences.
- Convenience sampling in the Nanping project and the relatively large proportion of CHARLS participants with missing values in CRP may have introduced bias.
- Residual confounding or hidden bias cannot be ruled out due to lack of information on some potential confounders, such as clinical cardiovascular risk factors (e.g, HDL-C, HbA1c), acute inflammatory conditions, medication use, etc.

Text

INTRODUCTION

C-reactive protein (CRP), a marker of systemic inflammation, has been shown to be involved in crucial pathogenesis in a variety of negative health outcomes, including cardiovascular

For peer review only - http://bmjopen.bmj.com/site/about/guidelines.xhtml

diseases,[1 2] diabetes,[3] cancer,[4] and cognitive decline.[5] Since the value of CRP in the prediction of prognoses in health outcomes has been recognized, it is important, from a public health perspective, to identify people at risk of elevated CRP in an efficient and simple way.

Self-rated health (SRH) refers to an individual's subjective perception of his/her own health and can be easily measured. Despite this, SRH has been featured as a strong predictor for functional ability,[6] chronic diseases,[7] and mortality.[8 9] Therefore, many health authorities have introduced SRH for surveillance.[10] The association between SRH and CRP has been examined in previous studies, but the results were inconsistent.[11-14] These discrepancies may be due to differences in characteristics of the study populations (e.g., age and sex) and study design. For example, a Japanese study demonstrated an association between poor SRH and an elevated CRP value in women, but not in men (age range 40-69).[14] In contrast, in an US sample of younger adults (mean age 28.42±1.78), current SRH was not associated with CRP in women, whereas the association was shown in men.[13] Among hospital-based studies, poor SRH was associated with higher CRP in female patients with coronary heart disease,[12] but not in patients with breast cancer.[15] In community-based studies, there has been a cross-sectional association between SRH and CRP, [13 14] but no evidence indicating longitudinal association.[16]

As SRH measures personal perception of health, it can be influenced by other factors beyond the real health status. For example, people with different educational levels may have different perceptions of health.[17] This education-related difference in perception of health may further play a role in the association between SRH and health outcomes. Indeed, a stronger association between SRH and mortality among higher educated than lower educated individuals has been shown in two studies.[18 19] Since CRP has been recognized as an important predicator of mortality,[20] education seems to modify its relationship with SRH.[21] It is noteworthy that studies

For peer review only - http://bmjopen.bmj.com/site/about/guidelines.xhtml

concerning the association between SRH and CRP were mostly conducted in developed countries where the study populations were relatively well educated.[11-14] To our knowledge, no study has focused on the difference in the association between SRH and CRP between illiterate and literate people. In China, despite the decrease in illiteracy from 1990 to 2010, there continues to be large difference between urban and rural areas: the rate of illiteracy in rural areas is two times more than that of urban areas.[22] Considering the lack of resources in rural areas, identifying people at risk of negative health outcomes using a simple measure such as SRH is warranted.

In the current study, we use two databases from China to examine the association between SRH and CRP among middle-aged and older people in rural areas, and to explore whether the SRH-CRP association varies across age (45-60/≥60), sex (men/women), and educational levels (illiterate/literate).

METHODS

Study population

Nanping project (NP)

NP is a 2015, voluntary participation, cross-sectional study consisting of residents aged 18 years or older from one county of Nanping City in Fujian Province, China. Seven villages were selected based on recommendations from local health workers, since the residents in these areas are known to be highly cooperative.

As showed in Figure 1, a total of 797 people were enrolled in the NP. To match with the age range of study population from the CHARLS, we excluded 98 participants under 45 years old. Those with CRP concentrations higher than 6.25 mg/L in dried blood spots (DBS), which is

For peer review only - http://bmjopen.bmj.com/site/about/guidelines.xhtml

comparable to 10 mg/L at serum level[23] (n=25), were excluded due to potential acute inflammatory conditions. After further excluding people with missing information on CRP (n=2), SRH (n=25), and on both CRP and SRH (n=1), 646 people remained in current study.

China Health and Retirement Longitudinal Study (CHARLS)

The CHARLS is a nationally representative longitudinal study. Eligible people were selected through a multistage probability sampling, and detailed descriptions of sampling method are provided in the users' guide.[24] In this current study, we used data from the baseline survey in 2011 because the CRP data was only available in that year. This is a secondary analysis of the CHARLS public database.

Overall, 17430 people were examined at baseline (Figure 1). People who lived in communities, or in both villages and communities (n=4562), and had CRP>10mg/L (n=429) were excluded. We further excluded people with missing data on CRP (n=3810), SRH (n=28), and on both CRP and SRH (n=46). Finally, 8555 (69%) people were included in the analytical sample.

Self-rated health (SRH)

SRH was assessed by one question: 'In general how would you rate your health?' Response options were 'good', 'average', 'poor', and 'very poor'.

C-reactive protein (CRP)

NP

Finger prick blood samples were collected by health workers using a filter paper, known as DBS. We kept the DBS at room temperature for a few days after being desiccated during the investigation period, then stored them in the Fujian Medical University at –20°. We used high

For peer review only

sensitivity sandwich enzyme immunoassay method to measure CRP concentrations by applying monoclonal antibodies.[23] Further details of the protocols have been presented elsewhere.[25]

CHARLS

The venous blood samples were collected by trained staff from local Chinese Center for Disease Control and Prevention (China CDC). Plasma samples were collected and preserved in 0.5 mL cryovial at -20°C, delivered to Beijing CDC within 2 weeks. Plasma CRP was determined by the immunoturbidimetric assay method at Capital Medical University.[26]

Covariates

In both cohorts, all participants were interviewed face-to-face by trained interviewers using a questionnaire that covers information on age, sex, education, marital status, smoking, alcohol consumption, and health status. Height and weight were measured by interviewers using standard anthropometers.

Education level was determined by maximum years of schooling: 0 year (illiterate), 1-6 years (elementary school), 7-9 years (junior high school), 10-12 years (senior high school), >12 years (college or above). Due to the fact that more than 30% of both the NP and CHARLS samples were illiterate, we dichotomized education into 0 year (illiterate) and >0 year (literate). Age was dichotomized as 45-60 years versus ≥60 years old, and marital status as married versus non-married. BMI was calculated by dividing weight (kg) by height squared (m^2) and categorized as underweight (<18.5), normal weight (18.5-24.99), overweight (25-29.99), and obese (≥30). Smoking was dichotomized into current smokers and non-current smokers (including former smokers). Alcohol consumption was categorized as regular drinkers (more

For peer review only - http://bmjopen.bmj.com/site/about/guidelines.xhtml

than 3 times per week) and non-regular drinkers.

Health status was measured by asking the participants whether they had any moderate/severe disease symptoms (e.g., fever) in the last month, or used antihypertensive or antidiabetic medications in the NP, and whether they had ever been diagnosed by a doctor with any diseases (e.g., hypertension), or often suffered from any pain currently in CHARLS. People answering positively were categorized as unhealthy, otherwise healthy.

Statistical analysis

First, data from the NP and CHARLS were analyzed separately. We applied one-way ANOVA to examine the differences of CRP in characteristics in each dataset by using F-distribution. The CRP variable was log-transformed because it was not normally distributed. The association between SRH and CRP was estimated by β-coefficient and a 95% confidence interval (CI) using linear regression in two datasets. The first estimate was respective; in the second, datasets were pooled. Fixed-effect meta-analysis was used to examine the heterogeneity. Then we re-ran the linear regression using the pooled dataset.

Age, sex and education were introduced into the basic-adjusted model. Further, we additionally adjusted for marital status, smoking, alcohol consumption, BMI, and health status.[27 28] All analyses were repeated in the stratified analyses by age, sex and levels of education.

In order to compare our results with previous studies that including participant with formal education only, we performed additional linear regression analysis stratified by age and sex among illiterate and literate participants separately.

For peer review only - http://bmjopen.bmj.com/site/about/guidelines.xhtml

All statistical analyses were performed with Stata 13.0 (Stata Corp, College Station, TX, USA).

Patient and public involvement

There were no participants involved in the development of this study.

RESULTS

Characteristics of the participants

The CRP levels across different characteristics of participants were compared in each dataset separately. Table 1 shows that in both datasets that older age people, higher BMI's, poorer SRH, or an unhealthy status were more likely to have elevated levels of CRP. The findings were inconsistent with sex, education, marital status, smoking and alcohol consumption in the two datasets. People with missing CRP values in NP and CHARLS were better educated and reported better health status compared to those who remained in the analyses (Supplementary File: Table S1 and Table S2).

For peer review only

For peer review only - http://bmjopen.bmj.com/site/about/guidelines.xhtml

Table 1 CRP values across characteristics of the study population

	NP (n=646)		CHARLS (n=8555)	
	Median (IQR) [a]	P[b]	Median (IQR) [a]	P[b]
Age		<0.001		<0.001
45-60	0.6 (0.3 to 1.2)		0.9 (0.5 to 1.7)	
≥60	0.8 (0.4 to 1.8)		1.1 (0.6 to 2.1)	
Sex		0.011		0.003
Men	0.6 (0.3 to 1.3)		1.0 (0.5 to 2.0)	
Women	0.8 (0.4 to 1.7)		0.9 (0.5 to 1.8)	
Education		0.004		0.316
Illiterate	0.9 (0.4 to 1.8)		1.0 (0.5 to 2.0)	
Literate	0.6 (0.3 to 1.3)		0.9 (0.5 to 1.9)	
Marital status		0.495		<0.001
Married	0.7 (0.3 to 1.5)		0.9 (0.5 to 1.9)	
Non-married	0.7 (0.4 to 1.7)		1.1 (0.6 to 2.4)	
Smoking		0.467		0.041
Current smokers	0.6 (0.3 to 1.4)		1.0 (0.5 to 2.0)	
Non-current smokers	0.7 (0.4 to 1.6)		0.9 (0.5 to 1.9)	
Alcohol consumption		0.001		0.635
Regular drinkers	0.5 (0.3 to 1.1)		0.9 (0.5 to 1.9)	
Non-regular drinkers	0.8 (0.4 to 1.6)		1.0 (0.5 to 1.9)	
BMI		<0.001		<0.001
Underweight (<18.5)	0.5 (0.2 to 1.4)		0.8 (0.5 to 1.9)	
Normal weight (18.5-24.99)	0.6 (0.3 to 1.1)		0.8 (0.5 to 1.7)	

For peer review only

Overweight (25-29.99)	1.2 (0.6 to 2.3)		1.2 (0.7 to 2.3)	
Obese (≥30)	1.6 (1.0 to 4.4)		1.9 (0.9 to 3.3)	
Self-rated health		0.071		<0.001
Good	0.6 (0.3 to 1.7)		0.9 (0.5 to 1.8)	
Average	0.7 (0.3 to 1.5)		0.9 (0.5 to 1.8)	
Poor	0.8 (0.4 to 1.5)		1.0 (0.6 to 2.1)	
Very poor	1.0 (0.5 to 2.3)		1.1 (0.6 to 2.3)	
Health status[c]		0.002		<0.001
Healthy	0.5 (0.3 to 1.3)		0.8 (0.5 to 1.7)	
Unhealthy	0.8 (0.4 to 1.6)		1.0 (0.5 to 2.0)	

[a] Median (interquartile range)

[b] ANOVA was applied to compare the mean of log-transformed values of CRP.

[c] Health status:

Unhealthy: Self-reported moderate to severe symptoms in the last month or used antihypertensive or antidiabetic medications (NP); Had been diagnosed by a doctor with any disease or often suffered from any pain currently (CHARLS). Healthy: no such report.

Missing values:
NP: 1 missing in health status.
CHARLS: 2 missing in age, 7 missing in sex, 4 missing in education,1 missing in smoking, 3 missing in alcohol consumption, 1191 missing in BMI, 65 missing in health status.

For peer review only

For peer review only - http://bmjopen.bmj.com/site/about/guidelines.xhtml

SRH and CRP

Table 2 presents the association between SRH and CRP in the two individual populations. In the NP, a borderline statistically significant association was observed between very poor SRH and elevated levels of CRP (β=0.39, 95%CI -0.07 to 0.85) in basic-adjusted model, while the association was attenuated after adjusting for confounders (β=0.29, 95%CI -0.15 to 0.73). Despite insignificance, the estimated effect of SRH started to change direction from average SRH (β = -0.05) to poor SRH (β = 0.10). In CHARLS, poor and very poor SRH were both associated with higher CRP (β=0.06, 95%CI 0.00 to 0.12; β=0.11, 95%CI 0.01 to 0.22). Considering the same pattern in both two datasets that poor and very poor SRH have similar effect on CRP and so as good and average SRH, and that there are limited number of participants with very poor SRH in NP, we combined 'good' and 'average' as good SRH, 'poor' and 'very poor' as poor SRH. Further, we found that poor SRH was associated with higher levels of CRP both in NP (β=0.16, 95%CI -0.02 to 0.34) and CHARLS (β=0.07, 95%CI 0.02 to 0.11) (Table 2).

Table 2 Association between self-rated health and C-reactive protein

		Model1[a]		Model2[b]	
	**N**	**β (95%CI)**	**P**	**β (95%CI)**	**P**
**NP**					
Good health	188	Ref.		Ref.	
Average	270	-0.03 (-0.22 to 0.17)	0.792	-0.05 (-0.24 to 0.14)	0.589
Poor	165	0.12 (-0.10 to 0.34)	0.292	0.10 (-0.11 to 0.32)	0.349
Very Poor	23	0.39 (-0.07 to 0.85)	0.093	0.29 (-0.15 to 0.73)	0.202
Good/Poor[c]	458/188	0.17 (-0.01 to 0.35)	0.067	0.16 (-0.02 to 0.34)	0.077
**CHARLS**					
Good health	1794	Ref.		Ref.	
Average	4157	0.01 (-0.04 to 0.06)	0.613	0.00 (-0.05 to 0.06)	0.911
Poor	2157	0.10 (0.04 to 0.15)	0.001	0.06 (0.00 to 0.12)	0.055
Very Poor	447	0.16 (0.06 to 0.25)	0.001	0.11 (0.01 to 0.22)	0.036
Good/Poor	5951/2604	0.10 (0.05 to 0.14)	<0.001	0.07 (0.02 to 0.11)	0.004
**NP+CHARLS**					
Good health	1982	Ref.		Ref.	
Average	4427	0.02 (-0.03 to 0.07)	0.379	0.01 (-0.04 to 0.06)	0.643

For peer review only - http://bmjopen.bmj.com/site/about/guidelines.xhtml

Poor	2322	0.11 (0.05 to 0.16)	<0.001	0.08 (0.02 to 0.14)	0.013
Very Poor	470	0.18 (0.09 to 0.28)	<0.001	0.14 (0.04 to 0.24)	0.007
Good/Poor	6409/2792	0.11 (0.06 to 0.15)	<0.001	0.08 (0.03 to 0.12)	0.001

[a] Adjusted for age, sex, education

[b] Adjusted for age, sex, education, marital status, smoking, alcohol consumption, BMI, health status

[c] Good= Good+Average, Poor=Poor+Very Poor

As the same direction of effect of estimate and a very low level of heterogeneity (I-squared< 0.001%) were observed in the two datasets (data not shown), we pooled the data and re-ran the linear regression analyses in the combined populations. The association between poorer SRH and higher CRP was observed in the pooled population (β=0.08, 95%CI 0.03 to 0.12) (Table2).

The roles of age, sex, and education in the association between SRH and CRP

The association between SRH and CRP stratified by age, sex, education is showed in Figure 2. In middle-aged people, worse SRH was associated with higher CRP both in NP (β=0.42, 95%CI 0.14 to 0.71) and CHARLS (β=0.06, 95%CI -0.01 to 0.12). Among older people, a similar trend was observed in CHARLS (β=0.08, 95%CI 0.02 to 0.15), but not in the NP. When stratified by sex, we found a statistically significant SRH-CRP association among men both in NP (β=0.27, 95%CI -0.03 to 0.57) and CHARLS (β=0.12, 95%CI 0.05 to 0.19), but not in women. In a stratified analysis by education, the association between SRH and CRP was seen in literate people both in NP (β=0.26, 95%CI 0.02 to 0.51) and CHARLS (β=0.11, 95%CI 0.05 to 0.16), but not in illiterate people.

In the pooled population, the SRH-CRP association was repeated in the middle-aged (β=0.08, 95%CI 0.02 to 0.14), older people (β=0.08, 95%CI 0.02 to 0.15), men (β=0.13, 95%CI 0.06 to 0.20), and literate people (β=0.12, 95%CI 0.06 to 0.18) (Figure 2).

Additional analyses

For peer review only - http://bmjopen.bmj.com/site/about/guidelines.xhtml

Identical trends with respect to the modifying effect of age and sex on the association between SRH and CRP were observed among literate people, but not among illiterate people (Supplementary File: Table S3).

DISCUSSION

In this study, based on 9201 residents in rural area of China, we found that poor SRH was associated with an elevated level of CRP in middle-aged and older people, especially among the men and literate.

Our finding of the association between poorer SRH and higher CRP level was in line with results from previous studies that included participants at similar age as our study participants.[11 14] Yet, those studies mainly included people living in industrialized countries with higher education, while our participants resided in less developed country with features of low literacy.

Possible pathways linking poor SRH and an elevated level of CRP could be related to psychological stress and health behaviors. Poor SRH may reflect a poor physical (e.g., inaccessibility to health service) and social (e.g., limited social network) environment, which can limit one's coping ability and induce psychological stress. It is known that stress can activate the sympathetic nervous system and the hypothalamic-pituitary-adrenal axis, contributing to the production of stress hormones, which in turn increase the secretion of CRP.[29 30] In addition, people with poor SRH were less likely to have an active lifestyle.[31] Having an inactive lifestyle has been suggested to potentially weaken the immune system and facilitate the inflammation processes through the release of pro-inflammatory adipokines.[32]

For peer review only - http://bmjopen.bmj.com/site/about/guidelines.xhtml

It is notable that poor SRH was associated with an elevated CRP level in literate participants, but not in the illiterate participants, which was consistent with one previous study.[21] Similar findings were also shown in studies focusing on SRH and mortality.[18 19] One of the possible explanations may be that illiterate people are often lack of health-related knowledge and access to health care,[17] and thus may misinterpret the feeling that they have in their bodies.[33] It has been shown that poor SRH in the less educated people mainly represents less serious diseases.[34] In our study, we also found that illiterate people were more likely to rate their health as poor and to report illness or pain both in NP and CHARLS. Moreover, illiterate people may have to withstand more pressure as they have less social and financial resources. Thus, other factors may contribute to the reported poor SRH, rather than actual health condition.

We found that SRH-CRP associations were only observed in men, but not in women, which may be due to the potential sex differences in reporting SRH. Previous studies have shown that the poor SRH in women can reflect both serious and non-serious diseases, whereas it tends to reflect serious diseases in men.[35] Broad dimensions of health perceptions may lead to less accurate SRH in women. In addition, the proportion of illiterate people among women is much higher than that among men in both datasets. This may explain the inconsistent findings between our study (6% participants with more than 9 years of schooling) and the Iwate-KENCO study from Japan, in which the corresponding figure was 46%.[14]

Findings from two datasets were not completely consistent. The association between poor SRH and elevated CRP values among older people (aged ≥60 years) was observed in CHARLS, but not in NP. In both populations, poor SRH was only associated with higher CRP in men, not in women. One of the explanations for these findings may be related to educational levels in the two study populations. Indeed, the proportion of illiterate people was relatively higher in older

For peer review only - http://bmjopen.bmj.com/site/about/guidelines.xhtml

adults in NP (76.2%) than in CHARLS (58.3%), and there was a higher proportion of illiterate people in women in both populations. Second, we observed similar age and sex differences in the associations between SRH and CRP among the literate: poor SRH was associated with elevated CRP values, especially in men, which was the same as the main results. This suggests that education might play a role in the SRH-CRP association.

This study provides evidence that SRH, a simple measurement, may be used as an indicator of bad physical health among middle-aged and older literate people, but not among the illiterate people, in rural area. In China, the implementation of health surveillance is more challenging in rural than in urban areas because of the discrepant aging processes,[36] knowledge gaps[22] and income inequality between these two areas. Elevated CRP has been associated with various physical[1-4] and psychological health outcomes. [37 38] Thus, our results support the consideration of using an efficient and cost-effective way, such as SRH, to monitor the health status in rural population where medical resources are limited. Future studies are needed to confirm our results and extend these findings to larger and more diverse populations. Moreover, identification of simple health indictors for illiterate people are warranted.

Acknowledgments We would like to express our sincere gratitude to the participants and local staff in NP project. We are grateful for those supporters: Harvard University (Aki Yazawa); National Center for Global Health and Medicine, Japan (Yosuke Inoue); Nagasaki Prefectural Institute of Environment and Public Health (Guoxi Cai); Fujian Medical University (Fei He, Jie Chen); Fujian Provincial Center for Disease Control and Prevention (Meng Huang) during the data collection in NP. Data from China Health and Retirement Longitudinal Study (CHARLS) were collected by the National School of Development at Peking University, China. We appreciated to University of Copenhagen (Tianwei Xu); Fujian Provincial Center for

For peer review only - http://bmjopen.bmj.com/site/about/guidelines.xhtml

Disease Control and Prevention (Xiuquan Lin); Nagasaki University (Sabin Nundu) for providing valuable comments in analysis and interpretation of data.

Contributors HW, RT, and KP conceptualized the study. RT analyzed the data and drafted the manuscript. HW, KP, GC, TY contributed to critical revisions of the manuscript. RT and HW are responsible for ensuring the integrity and accuracy of the study. All authors have read and approved the final manuscript.

Funding This study is financed by the Program for Nurturing Global Leaders in Tropical and Emerging Communicable Diseases, Graduate School of Biomedical Sciences, Nagasaki University, Japan (Raoping Tu); the Swedish Research Council (Grant no: 2018-02998) and the Swedish Research Council for Health, Working Life and Welfare (Forte) (2019-01120) (Hui-Xin Wang); the Ministry of Education of Taiwan, the Swedish National Graduate School on Ageing and Health (SWEA), and Gamla Tjänarinnor Foundation (Kuan-Yu Pan). NP was financially supported by the JSPS KAKENHI from the Japan Society for the Promotion of Science (13J06172).

Competing Interests None declared.

Patient consent Not required.

Ethics approval The Ethics Committee for Medical Research at the University of Tokyo (No. 10515-(1)) and the Ethics Committee of the Institute of Tropical Medicine at Nagasaki University (No.120910100-5) approved the study protocol of NP. The Medical Ethics Committee of Peking University approved the research protocol of CHARLS.

For peer review only

Provenance and peer review Not commissioned; externally peer reviewed

Data sharing statement All of the CHARLS data will be accessible to researchers around the world at the CHARLS project website (http://charls.pku.edu.cn/en). No additional data available.

Figure legends

Figure 1 Flowchart of the study populations in NP and CHARLS

Figure 2 β-coefficient and 95% confidence interval (CI) of CRP in relation to poor self-rated health from linear regression models stratified by age, sex and education in NP, CHARLS, and the pooled populations of the two datasets. SRH is dichotomized as poor to very poor versus good to average. When stratified by age, models are adjusted for sex, education, marital status, smoking, alcohol consumption, BMI, health status; when stratified by sex, models are adjusted for age, education, marital status, smoking, alcohol consumption, BMI, health status; when stratified by education, models are adjusted for age, sex, marital status, smoking, alcohol consumption, BMI, health status.

SUPPLEMENTARY FILE

Table S1 Characteristics of study sample in NP without and with missing values in CRP

Table S2 Characteristics of study sample in CHARLS without and with missing values in CRP

Table S3 Association between self-rated health and C-reactive protein among illiterate and literate people: stratified by age and sex

For peer review only

References

1 Black PH, Garbutt LD. Stress, inflammation and cardiovascular disease. *Journal of psychosomatic research* 2002;52:1-23.

2 Ridker PM. From C-Reactive Protein to Interleukin-6 to Interleukin-1. *Circulation Research* 2016;118:145-56.

3 Barzilaym JI, Freedland ES. Inflammation and its relationship to insulin resistance, type 2 diabetes mellitus, and endothelial dysfunction. *Metabolic syndrome and related disorders* 2003;1:55-67.

4 Wang J, Lee IM, Tworoger SS, *et al*. Plasma C-reactive protein and risk of breast cancer in two prospective studies and a meta-analysis. *Cancer Epidemiol Biomarkers Prev* 2015;24:1199-206.

5 Panza F, Solfrizzi V, Logroscino G, *et al*. Current epidemiological approaches to the metabolic-cognitive syndrome. *J Alzheimers Dis* 2012;30 Suppl 2:S31-75.

6 Idler EL, Kasl SV. Self-ratings of health: do they also predict change in functional ability? *J Gerontol B Psychol Sci Soc Sci* 1995;50:S344-53.

7 Latham K, Peek CW. Self-Rated Health and Morbidity Onset Among Late Midlife U.S. Adults. *The Journals of Gerontology Series B: Psychological Sciences and Social Sciences* 2012;68:107-16.

8 DeSalvo KB, Bloser N, Reynolds K, *et al*. Mortality prediction with a single general self-rated health question. A meta-analysis. *J Gen Intern Med* 2006;21:267-75.

9 Singh-Manoux A, Dugravot A, Shipley MJ, *et al*. The association between self-rated health and mortality in different socioeconomic groups in the GAZEL cohort study. *Int J Epidemiol* 2007;36:1222-8.

10 Salomon JA, Nordhagen S, Oza S, *et al.* Are Americans feeling less healthy? The puzzle

For peer review only - http://bmjopen.bmj.com/site/about/guidelines.xhtml

of trends in self-rated health. *Am J Epidemiol* 2009;170:343-51.

11 Christian LM, Glaser R, Porter K, *et al*. Poorer self-rated health is associated with elevated inflammatory markers among older adults. *Psychoneuroendocrinology* 2011;36:1495-504.

12 Janszky I, Lekander M, Blom M, *et al*. Self-rated health and vital exhaustion, but not depression, is related to inflammation in women with coronary heart disease. *Brain Behav Immun* 2005;19:555-63.

13 Shanahan L, Bauldry S, Freeman J, *et al*. Self-rated health and C-reactive protein in young adults. *Brain, Behavior, and Immunity* 2014;36:139-46.

14 Tanno K, Ohsawa M, Onoda T, *et al*. Poor self-rated health is significantly associated with elevated C-reactive protein levels in women, but not in men, in the Japanese general population. *Journal of Psychosomatic Research* 2012;73:225-31.

15 Orre IJ, Reinertsen KV, Aukrust P, *et al*. Higher levels of fatigue are associated with higher CRP levels in disease-free breast cancer survivors. *J Psychosom Res* 2011;71:136-41.

16 Tamura T, Naito M, Maruyama K, et al. The association between self-rated health and high-sensitivity C-reactive protein level: a cross-sectional and 5-year longitudinal study. BMC Public Health 2018;18(1):1380. doi: 10.1186/s12889-018-6251-6

17 Spuling SM, Huxhold O, Wurm S. Predictors of Self-Rated Health: Does Education Play a Role Above and Beyond Age? *J Gerontol B Psychol Sci Soc Sci* 2017;72:415-24.

18 Dowd JB, Zajacova A. Does the predictive power of self-rated health for subsequent mortality risk vary by socioeconomic status in the US? *Int J Epidemiol* 2007;36:1214-21.

19 Huisman M, van Lenthe F, Mackenbach J. The predictive ability of self-assessed health for mortality in different educational groups. *Int J Epidemiol* 2007;36:1207-13.

20 Li Y, Zhong X, Cheng G, et al. Hs-CRP and all-cause, cardiovascular, and cancer mortality risk: A meta-analysis. Atherosclerosis 2017;259:75-82.

21 Dowd JB, Zajacova A. Does self-rated health mean the same thing across socioeconomic

For peer review only - http://bmjopen.bmj.com/site/about/guidelines.xhtml

groups? Evidence from biomarker data. *Ann Epidemiol* 2010;20:743-9.

22 Guo S, Guo Y. *Spotlight on China: Changes in education under China's market economy*: Springer, 2016.

23 Brindle E, Fujita M, Shofer J, *et al*. Serum, plasma, and dried blood spot high-sensitivity C-reactive protein enzyme immunoassay for population research. *J Immunol Methods* 2010;362:112-20.

24 Zhao Y, Strauss J, Yang G, *et al. China health and retirement longitudinal study–2011–2012 national baseline users' guide*. National School of Development, Peking University, 2013.

25 Yazawa A, Inoue Y, Cai G, *et al*. Epstein-Barr virus antibody titer as a stress biomarker and its association with social capital in rural Fujian communities, China. *Am J Hum Biol* 2018:e23135.

26 Zhao Y, Crimmins E, Hu P, *et al. CHARLS Blood Sample Users' Guide*. National School of Development, Peking University, 2014.

27 Kiecolt-Glaser JK, Gouin JP, Hantsoo L. Close relationships, inflammation, and health. *Neurosci Biobehav Rev* 2010;35:33-8.

28 Zheng H, Thomas PA. Marital status, self-rated health, and mortality: overestimation of health or diminishing protection of marriage? *Journal of health and social behavior* 2013;54:128-43.

29 Coussons-Read ME, Okun ML, Nettles CD. Psychosocial stress increases inflammatory markers and alters cytokine production across pregnancy. *Brain Behav Immun* 2007;21:343-50.

30 Shivpuri S, Gallo LC, Crouse JR, *et al.* The association between chronic stress type and C-reactive protein in the multi-ethnic study of atherosclerosis: does gender make a difference? *J Behav Med* 2012;35:74-85.

For peer review only - http://bmjopen.bmj.com/site/about/guidelines.xhtml

31 Molarius A, Berglund K, Eriksson C, *et al*. Socioeconomic conditions, lifestyle factors, and self-rated health among men and women in Sweden. *European journal of public health* 2007;17:125-33.

32 Gleeson M, Bishop NC, Stensel DJ, *et al*. The anti-inflammatory effects of exercise: mechanisms and implications for the prevention and treatment of disease. *Nat Rev Immunol* 2011;11:607-15.

33 Franks P, Gold MR, Fiscella K. Sociodemographics, self-rated health, and mortality in the US. *Social Science & Medicine* 2003;56:2505-14.

34 Regidor E, Guallar-Castillon P, Gutierrez-Fisac JL, *et al.* Socioeconomic variation in the magnitude of the association between self-rated health and mortality. *Ann Epidemiol* 2010;20:395-400.

35 Benyamini Y, Leventhal EA, Leventhal H. Gender differences in processing information for making self-assessments of health. *Psychosomatic Medicine* 2000;62:354-64.

36 Gong P, Liang S, Carlton EJ, *et al.* Urbanisation and health in China. *The Lancet* 2012;379(9818):843-52.

37 Valkanova V, Ebmeier KP, Allan CL. CRP, IL-6 and depression: a systematic review and meta-analysis of longitudinal studies. *J Affect Disord* 2013;150(3):736-44.

38 Wium-Andersen MK, Orsted DD, Nielsen SF, *et al.* Elevated C-reactive protein levels, psychological distress, and depression in 73, 131 individuals. *JAMA Psychiatry* 2013;70(2):176-84.

For peer review only

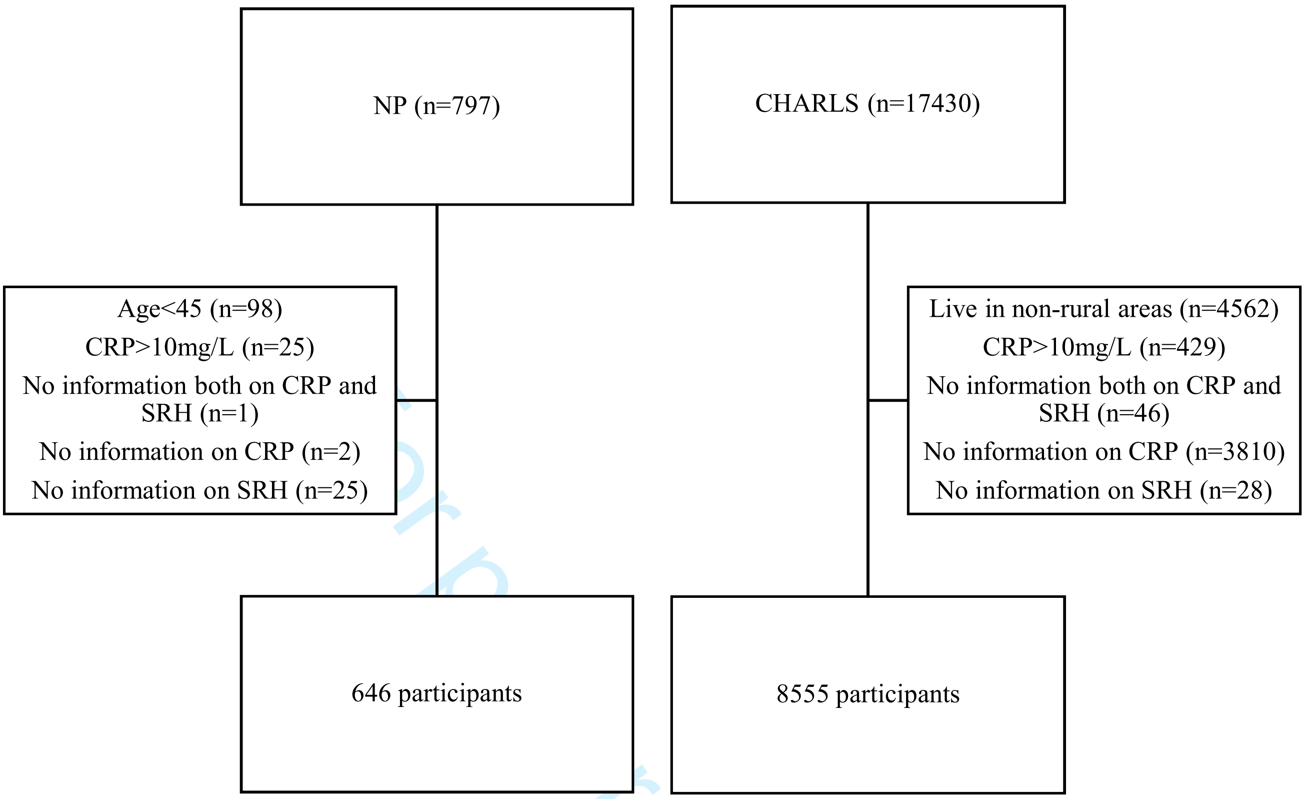

Fig 1 Flowchart of the study populations in NP and CHARLS

For peer review only

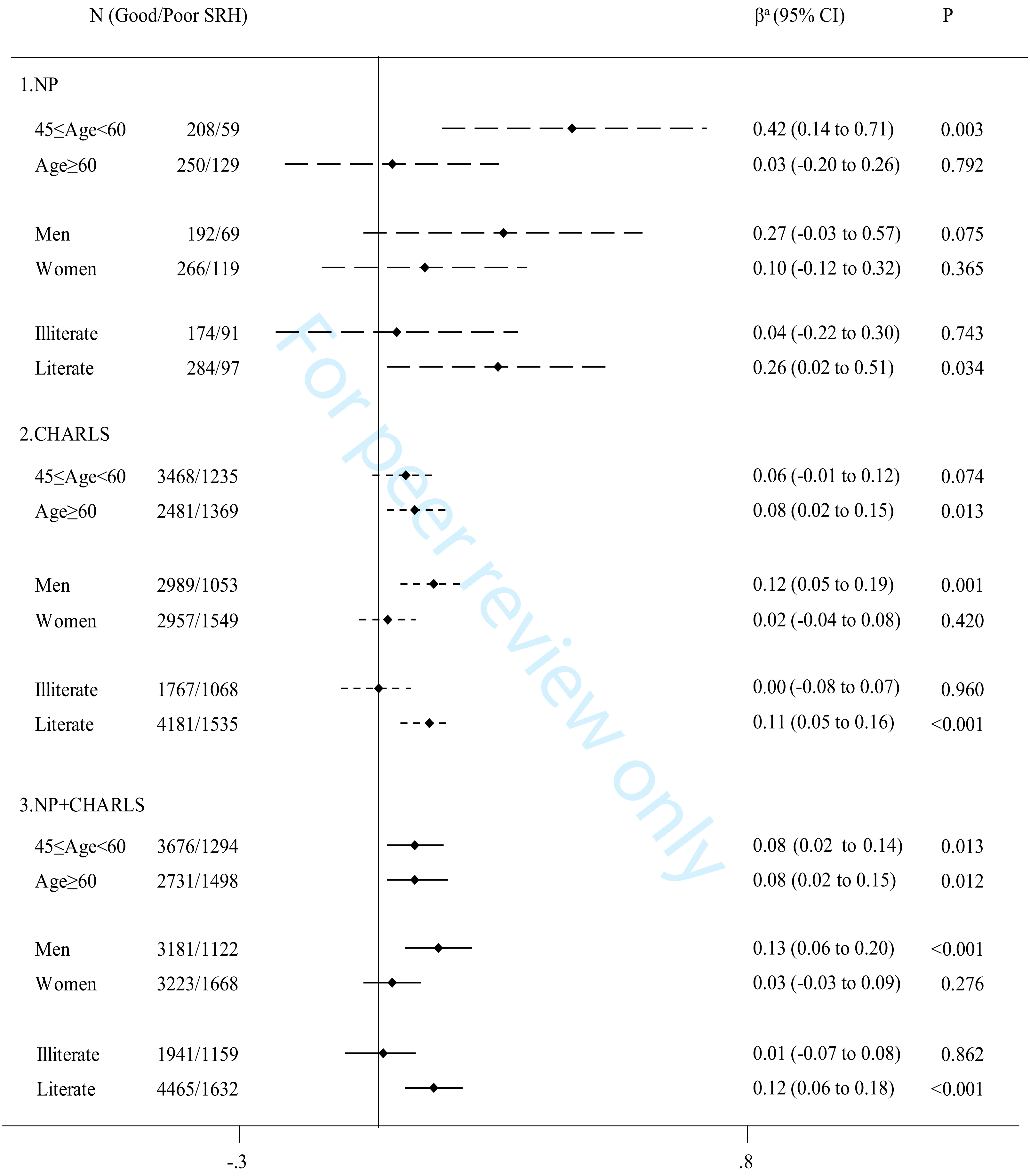

For peer review only

Figure 2 β-coefficient and 95% confidence interval (CI) of CRP in relation to poor self-rated health from linear regression models stratified by age, sex and education in NP, CHARLS, and the pooled populations of the two datasets. SRH is dichotomized as poor to very poor versus good to average. When stratified by age, models are adjusted for sex, education, marital status, smoking, alcohol consumption, BMI, health status; when stratified by sex, models are adjusted for age, education, marital status, smoking, alcohol consumption, BMI, health status; when stratified by education, models are adjusted for age, sex, marital status, smoking, alcohol consumption, BMI, health status.

[a] The average CRP changes in response to one-unit shift in SRH.

For peer review only - http://bmjopen.bmj.com/site/about/guidelines.xhtml

SUPPLEMENTARY FILE

Table S1 Characteristics of study sample in NP without and with missing values in CRP

	Non-missing[a] (n=646)	Missing[a] (n=2)	P
Age			0.093
<60	267(41.3)	2(100)	
≥60	379(58.7)	0(0)	
Sex			0.245
Men	261(40.4)	0(0)	
Women	385(59.6)	2(100)	
Education			0.239
Illiterate	265(41.0)	0(0)	
Literate	381(59.0)	2(100)	
Marital status			0.491
Married	522(80.8)	2(100)	
Non-married	124(19.2)	0(0)	
Smoking			0.582
Current smokers	85(13.2)	0(0)	
Non-current smokers	561(86.8)	2(100)	
Drinking			0.489
Regular drinkers	125(19.4)	0(0)	
Non-regular drinkers	521(80.7)	2(100)	
BMI			0.810
Underweight (<18.5)	30(4.60)	0(0)	
Normal weight (18.5-25)	436(67.5)	2(100)	
Overweight (25-30)	158(24.5)	0(0)	
Obese (≥30)	22(3.4)	0(0)	
Self-rated health			0.184
Good	188(29.1)	2(100)	
Average	270(41.8)	0(0)	
Poor	165(25.5)	0(0)	
Very poor	23(3.6)	0(0)	
Health status[b]			0.018
Healthy	127(19.7)	2(100)	
Unhealthy	518(80.2)	0(0)	
Missing	1(0.2)	0(0)	

[a] Data are presented as n (%).

[b] Healthy status:

Unhealthy: Self-reported moderate to severe symptoms in the last month or used antihypertensive or antidiabetic medications (NP).

Healthy: No such report.

For peer review only

Table S2 Characteristics of study sample in CHARLS without and with missing values in CRP

	**Non-missing[a] (n=8555)**	**Missing[a] (n=3810)**	**P**
Age			0.002
<60	4703(55.0)	2226(58.4)	
≥60	3850(45.0)	1583(41.6)	
Missing	2(0)	1(0)	
Sex			<0.001
Men	4042(47.3)	2014(52.9)	
Women	4506(52.7)	1794(47.1)	
Missing	7(0.1)	2(0.1)	
Education			0.001
Illiterate	2835(33.1)	1160(30.5)	
Literate	5716(66.8)	2643(69.4)	
Missing	4(0.1)	7(0.2)	
Marital status			0.001
Married	7517(87.9)	3263(85.6)	
Non-married	1038(12.1)	547(14.4)	
Smoking			0.113
Current smokers	2561(29.9)	1086(28.5)	
Non-current smokers	5993(70.1)	2722(71.4)	
Missing	1(0)	2(0.1)	
Drinking			0.024
Regular drinkers	998(11.7)	399(10.5)	
Non-regular drinkers	7554(88.3)	3406(89.4)	
Missing	3(0)	5(0.1)	
BMI			<0.001
Underweight (<18.5)	535(6.3)	206(5.4)	
Normal weight (18.5-25)	4719(55.2)	1790(47.0)	
Overweight (25-30)	1819(21.3)	592(15.5)	
Obese (≥30)	291(3.4)	101(2.7)	
Missing	1191(13.9)	1121(29.4)	
Self-rated health			0.002
Good	1794(21.0)	910(23.9)	
Average	4157(48.6)	1798(47.2)	
Poor	2157(25.2)	894(23.5)	
Very poor	447(5.2)	208(5.5)	
Health status[b]			<0.001
Healthy	2089(24.4)	1160(30.5)	
Unhealthy	6401(74.8)	2607(68.4)	
Missing	65(0.8)	43(1.1)	

[a] Data are presented as n (%)
[b] Healthy status:
Unhealthy: Had been diagnosed by a doctor with any disease or often suffered from any pain currently (CHARLS).
Healthy: No such report.

For peer review only

Table S3 Association between self-rated health and C-reactive protein among illiterate and literate people: stratified by age and sex

	N	Age<60	N	Age≥60	N	Men	N	Women
**Illiterate**								
**NP**								
Good	47	Ref.	127	Ref.	56	Ref.	118	Ref.
Poor	16	0.35 (-0.27 to 0.97)	75	-0.01 (-0.32 to 0.29)	21	0.03(-0.53 to 0.59)	70	0.04 (-0.26 to 0.34)
**CHARLS**								
Good	777	Ref.	989	Ref.	406	Ref.	1359	Ref.
Poor	405	-0.07 (-0.19 to 0.05)	663	0.05 (-0.05 to 0.15)	232	-0.06(-0.24 to 0.11)	835	0.02 (-0.07 to 0.1)
**NP+CHARLS**								
Good	824	Ref.	1116	Ref.	462	Ref.	1477	Ref.
Poor	421	-0.05 (-0.17 to 0.07)	738	0.05 (-0.05 to 0.14)	253	-0.04(-0.2 to 0.13)	905	0.02 (-0.06 to 0.1)
**Literate**								
**NP**								
Good	161	Ref.	123	Ref.	136	Ref.	148	Ref.
Poor	43	0.47 (0.14 to 0.8)**	54	0.08 (-0.3 to 0.45)	48	0.4 (0.03 to 0.77)*	49	0.17 (-0.16 to 0.5)
**CHARLS**								
Good	2690	Ref.	1490	Ref.	2581	Ref.	1597	Ref.
Poor	829	0.11 (0.03 to 0.19)**	706	0.11 (0.02 to 0.19)*	821	0.16 (0.08 to 0.24)**	713	0.03 (-0.05 to 0.12)
**NP+CHARLS**								
Good	2851	Ref.	1613	Ref.	2717	Ref.	1745	Ref.
Poor	872	0.13 (0.06 to 0.21)**	760	0.11 (0.02 to 0.19)*	869	0.17 (0.1 to 0.25)**	762	0.04 (-0.04 to 0.13)

CHARLS: 1) Iliterate:1 missing in age, 3 missing in sex; 2) Literate: 1 missing in age, 4 missing in sex.
*P<0.05
**P<0.01

For peer review only

STROBE 2007 (v4) Statement—Checklist of items that should be included in reports of *cross-sectional studies*

Section/Topic	Item #	Recommendation	Reported on page #
**Title and abstract**	1	(*a*) Indicate the study's design with a commonly used term in the title or the abstract	P1
		(*b*) Provide in the abstract an informative and balanced summary of what was done and what was found	P3
**Introduction**			
Background/rationale	2	Explain the scientific background and rationale for the investigation being reported	P4-5
Objectives	3	State specific objectives, including any prespecified hypotheses	P5-6
**Methods**			
Study design	4	Present key elements of study design early in the paper	P6-7
Setting	5	Describe the setting, locations, and relevant dates, including periods of recruitment, exposure, follow-up, and data collection	P6-9
Participants	6	(*a*) Give the eligibility criteria, and the sources and methods of selection of participants	P6-7
Variables	7	Clearly define all outcomes, exposures, predictors, potential confounders, and effect modifiers. Give diagnostic criteria, if applicable	P7-9
Data sources/ measurement	8*	For each variable of interest, give sources of data and details of methods of assessment (measurement). Describe comparability of assessment methods if there is more than one group	P7-9
Bias	9	Describe any efforts to address potential sources of bias	P9
Study size	10	Explain how the study size was arrived at	P6-7
Quantitative variables	11	Explain how quantitative variables were handled in the analyses. If applicable, describe which groupings were chosen and why	P8-9
Statistical methods	12	(*a*) Describe all statistical methods, including those used to control for confounding	P9
		(*b*) Describe any methods used to examine subgroups and interactions	P9
		(*c*) Explain how missing data were addressed	P10,12
		(*d*) If applicable, describe analytical methods taking account of sampling strategy	P6-7
		(*e*) Describe any sensitivity analyses	P9
**Results**			

For peer review only

Participants	13*	(a) Report numbers of individuals at each stage of study—eg numbers potentially eligible, examined for eligibility, confirmed eligible, included in the study, completing follow-up, and analysed	Figure 1
		(b) Give reasons for non-participation at each stage	Figure 1
		(c) Consider use of a flow diagram	Figure 1
Descriptive data	14*	(a) Give characteristics of study participants (eg demographic, clinical, social) and information on exposures and potential confounders	P10-12
		(b) Indicate number of participants with missing data for each variable of interest	P12
Outcome data	15*	Report numbers of outcome events or summary measures	P11-12
Main results	16	(*a*) Give unadjusted estimates and, if applicable, confounder-adjusted estimates and their precision (eg, 95% confidence interval). Make clear which confounders were adjusted for and why they were included	P13-14, why: P9
		(*b*) Report category boundaries when continuous variables were categorized	P8-9
		(*c*) If relevant, consider translating estimates of relative risk into absolute risk for a meaningful time period	N/A
Other analyses	17	Report other analyses done—eg analyses of subgroups and interactions, and sensitivity analyses	P15
**Discussion**			
Key results	18	Summarise key results with reference to study objectives	P15
Limitations	19	Discuss limitations of the study, taking into account sources of potential bias or imprecision. Discuss both direction and magnitude of any potential bias	P4
Interpretation	20	Give a cautious overall interpretation of results considering objectives, limitations, multiplicity of analyses, results from similar studies, and other relevant evidence	P15-17
Generalisability	21	Discuss the generalisability (external validity) of the study results	P17
**Other information**			
Funding	22	Give the source of funding and the role of the funders for the present study and, if applicable, for the original study on which the present article is based	P18

*Give information separately for cases and controls in case-control studies and, if applicable, for exposed and unexposed groups in cohort and cross-sectional studies.

Note: An Explanation and Elaboration article discusses each checklist item and gives methodological background and published examples of transparent reporting. The STROBE checklist is best used in conjunction with this article (freely available on the Web sites of PLoS Medicine at http://www.plosmedicine.org/, Annals of Internal Medicine at http://www.annals.org/, and Epidemiology at http://www.epidem.com/). Information on the STROBE Initiative is available at www.strobe-statement.org.

For peer review only

BMJ Open

The role of education in the association between self-rated health and levels of C-reactive protein: a cross-sectional study in rural areas of China

Journal:	*BMJ Open*
Manuscript ID	bmjopen-2018-027659.R3
Article Type:	Original research
Date Submitted by the Author:	14-Oct-2019
Complete List of Authors:	Tu, Raoping; School of Nursing, Yangzhou University; Nagasaki University Institute of Tropical Medicine, Department of International Health and Medical Anthropology<br>Pan, Kuan-Yu; Karolinska Institutet, Stockholm University, Department of Neurobiology, Care Sciences and Society (NVS), Aging Research Center<br>Cai, Guoxi; Nagasaki Prefectural Institute of Environment and Public Health; Nagasaki University Institute of Tropical Medicine, Department of International Health and Medical Anthropology<br>Yamamoto, Taro; Nagasaki University Institute of Tropical Medicine, Department of International Health and Medical Anthropology<br>Wang, Hui-Xin; Stockholm University, Stress Research Institute; Karolinska Institutet, Department of Neurobiology, Care Sciences and Society (NVS), Aging Research Center
**Primary Subject Heading**:	Public health
Secondary Subject Heading:	Epidemiology
Keywords:	EPIDEMIOLOGY, PUBLIC HEALTH, SOCIAL MEDICINE

SCHOLARONE™
Manuscripts

Title page

The role of education in the association between self-rated health and levels of C-reactive protein: a cross-sectional study in rural areas of China

Author's names and affiliation:

Raoping Tu[1,2,3], Kuan-Yu Pan[4], Guoxi Cai [2,5], Taro Yamamoto [2,*], Hui-Xin Wang [4,6,*]

[1]School of Nursing, Yangzhou University, Yangzhou, China

[2]Department of International Health and Medical Anthropology, Institute of Tropical Medicine (NEKKEN), Nagasaki University, Nagasaki, Japan.

[3] Leading Program, Graduate School of Biomedical Sciences, Nagasaki University, Nagasaki, Japan.

[4] Aging Research Center, Department of Neurobiology, Care Sciences and Society (NVS), Karolinska Institutet, Stockholm University, Stockholm, Sweden.

[5]Nagasaki Prefectural Institute of Environment and Public Health, Nagasaki, Japan.

[6]Stress Research Institute, Stockholm University, Stockholm, Sweden.

*Correspondence to:

1. Department of International Health and Medical Anthropology, Institute of Tropical Medicine (NEKKEN), Nagasaki University, 1-12-4 Sakamoto, Nagasaki 852-8523, Japan

Tel: +81-095-819-7853, Fax: +81-095-819-7869

For peer review only - http://bmjopen.bmj.com/site/about/guidelines.xhtml

Taro Yamamoto
E-mail: y-taro@nagasaki-u.ac.jp
2. Stress Research Institute, Stockholm University, SE-106 91 Stockholm, Sweden
Tel: +46-08-5537-8941
Hui-Xin Wang
E-mail: huixin.wang@su.se

Word count:2982

For peer review only

For peer review only - http://bmjopen.bmj.com/site/about/guidelines.xhtml

ABSTRACT

Objectives This study aims to examine the association between self-rated health (SRH) and levels of C-reactive protein (CRP) among adults aged 45 to 101 years old in rural areas of China, and to explore the role of education in the association.

Design Cross-sectional study

Setting The study population was derived from two databases in China: Nanping project (NP) and the China Health and Retirement Longitudinal Study (CHARLS).

Participants 646 participants from a rural area of Nanping (NP) and 8555 rural participants from a national representative sample of China (CHARLS).

Methods CRP was measured using a high sensitivity sandwich enzyme immunoassay in the NP and immunoturbidimetric assay in the CHARLS. SRH was assessed by self-rated health questionnaires and categorized into good and poor. Education was measured by the maximum years of schooling and dichotomized into illiterate and literate. Multivariate linear regression models were used to study the associations.

Results Compared to people with good SRH, those with poor SRH had higher levels of CRP in NP (β=0.16, 95% CI -0.02 to 0.34) and in CHARLS (β=0.07, 95% CI 0.02 to 0.11). This was especially in men and literate people after adjusting for potential confounders. Similar findings were observed in the pooled population (β=0.08, 95% CI 0.03 to 0.12), in men (β=0.13, 95% CI 0.06 to 0.20), and in literate people (β=0.12, 95% CI 0.06 to 0.18).

Conclusion Poor SRH may be a predicator of elevated levels of CRP among middle-aged and older people in rural areas, especially in men and literate people.

For peer review only - http://bmjopen.bmj.com/site/about/guidelines.xhtml

Keywords Self-rated health; C-reactive protein; Education level

Strengths and limitations of this study

- Our study population came from two databases, including one national representative sample derived from the China Health and Retirement Longitudinal Study (CHARLS), making our results highly generalizable to the national rural population of China.
- C-reactive protein (CRP) was an objective measure performed by health professionals using validated methods, making it more reliable than subjective measures.
- Cross-sectional study design prevented us from making causal inferences.
- Convenience sampling in the Nanping project and the relatively large proportion of CHARLS participants with missing values in CRP may have introduced bias.
- Residual confounding or hidden bias cannot be ruled out due to lack of information on some potential confounders, such as clinical cardiovascular risk factors (e.g, HDL-C, HbA1c), acute inflammatory conditions, medication use, etc.

Text

INTRODUCTION

C-reactive protein (CRP), a marker of systemic inflammation, has been shown to be involved in crucial pathogenesis in a variety of negative health outcomes, including cardiovascular

For peer review only - http://bmjopen.bmj.com/site/about/guidelines.xhtml

diseases,[1 2] diabetes,[3] cancer,[4] and cognitive decline.[5] Since the value of CRP in the prediction of prognoses in health outcomes has been recognized, it is important, from a public health perspective, to identify people at risk of elevated CRP in an efficient and simple way.

Self-rated health (SRH) refers to an individual's subjective perception of his/her own health and can be easily measured. Despite this, SRH has been featured as a strong predictor for functional ability,[6] chronic diseases,[7] and mortality.[8 9] Therefore, many health authorities have introduced SRH for surveillance.[10] The association between SRH and CRP has been examined in previous studies, but the results were inconsistent.[11-14] These discrepancies may be due to differences in characteristics of the study populations (e.g., age and sex) and study design. For example, a Japanese study demonstrated an association between poor SRH and an elevated CRP value in women, but not in men (age range 40-69).[14] In contrast, in an US sample of younger adults (mean age 28.42±1.78), current SRH was not associated with CRP in women, whereas the association was shown in men.[13] Among hospital-based studies, poor SRH was associated with higher CRP in female patients with coronary heart disease,[12] but not in patients with breast cancer.[15] In community-based studies, there has been a cross-sectional association between SRH and CRP, [13 14] but no evidence indicating longitudinal association.[16]

As SRH measures personal perception of health, it can be influenced by other factors beyond the real health status. For example, people with different educational levels may have different perceptions of health.[17] This education-related difference in perception of health may further play a role in the association between SRH and health outcomes. Indeed, a stronger association between SRH and mortality among higher educated than lower educated individuals has been shown in two studies.[18 19] Since CRP has been recognized as an important predicator of mortality,[20] education seems to modify its relationship with SRH.[21] It is noteworthy that studies

For peer review only - http://bmjopen.bmj.com/site/about/guidelines.xhtml

concerning the association between SRH and CRP were mostly conducted in developed countries where the study populations were relatively well educated.[11-14] To our knowledge, no study has focused on the difference in the association between SRH and CRP between illiterate and literate people. In China, despite the decrease in illiteracy from 1990 to 2010, there continues to be large difference between urban and rural areas: the rate of illiteracy in rural areas is two times more than that of urban areas.[22] Considering the lack of resources in rural areas, identifying people at risk of negative health outcomes using a simple measure such as SRH is warranted.

In the current study, we use two databases from China to examine the association between SRH and CRP among middle-aged and older people in rural areas, and to explore whether the SRH-CRP association varies across age (45-60/≥60), sex (men/women), and educational levels (illiterate/literate).

METHODS

Study population

Nanping project (NP)

NP is a 2015, voluntary participation, cross-sectional study consisting of residents aged 18 years or older from one county of Nanping City in Fujian Province, China. Seven villages were selected based on recommendations from local health workers, since the residents in these areas are known to be highly cooperative.

As showed in Figure 1, a total of 797 people were enrolled in the NP. To match with the age range of study population from the CHARLS, we excluded 98 participants under 45 years old. Those with CRP concentrations higher than 6.25 mg/L in dried blood spots (DBS), which is

For peer review only - http://bmjopen.bmj.com/site/about/guidelines.xhtml

comparable to 10 mg/L at serum level[23] (n=25), were excluded due to potential acute inflammatory conditions. After further excluding people with missing information on CRP (n=2), SRH (n=25), and on both CRP and SRH (n=1), 646 people remained in current study.

China Health and Retirement Longitudinal Study (CHARLS)

The CHARLS is a nationally representative longitudinal study. Eligible people were selected through a multistage probability sampling, and detailed descriptions of sampling method are provided in the users' guide.[24] In this current study, we used data from the baseline survey in 2011 because the CRP data was only available in that year. This is a secondary analysis of the CHARLS public database.

Overall, 17430 people were examined at baseline (Figure 1). People who lived in communities, or in both villages and communities (n=4562), and had CRP>10mg/L (n=429) were excluded. We further excluded people with missing data on CRP (n=3810), SRH (n=28), and on both CRP and SRH (n=46). Finally, 8555 (69%) people were included in the analytical sample.

Self-rated health (SRH)

SRH was assessed by one question: 'In general how would you rate your health?' Response options were 'good', 'average', 'poor', and 'very poor'.

C-reactive protein (CRP)

NP

Finger prick blood samples were collected by health workers using a filter paper, known as DBS. We kept the DBS at room temperature for a few days after being desiccated during the investigation period, then stored them in the Fujian Medical University at –20°. We used high

For peer review only

sensitivity sandwich enzyme immunoassay method to measure CRP concentrations by applying monoclonal antibodies.[23] Further details of the protocols have been presented elsewhere.[25]

CHARLS

The venous blood samples were collected by trained staff from local Chinese Center for Disease Control and Prevention (China CDC). Plasma samples were collected and preserved in 0.5 mL cryovial at -20°C, delivered to Beijing CDC within 2 weeks. Plasma CRP was determined by the immunoturbidimetric assay method at Capital Medical University.[26]

For peer review only

Covariates

In both cohorts, all participants were interviewed face-to-face by trained interviewers using a questionnaire that covers information on age, sex, education, marital status, smoking, alcohol consumption, and health status. Height and weight were measured by interviewers using standard anthropometers.

Education level was determined by maximum years of schooling: 0 year (illiterate), 1-6 years (elementary school), 7-9 years (junior high school), 10-12 years (senior high school), >12 years (college or above). Due to the fact that more than 30% of both the NP and CHARLS samples were illiterate, we dichotomized education into 0 year (illiterate) and >0 year (literate). Age was dichotomized as 45-60 years versus ≥60 years old, and marital status as married versus non-married. BMI was calculated by dividing weight (kg) by height squared (m^2) and categorized as underweight (<18.5), normal weight (18.5-24.99), overweight (25-29.99), and obese (≥30). Smoking was dichotomized into current smokers and non-current smokers (including former smokers). Alcohol consumption was categorized as regular drinkers (more

For peer review only - http://bmjopen.bmj.com/site/about/guidelines.xhtml

than 3 times per week) and non-regular drinkers.

Health status was measured by asking the participants whether they had any moderate/severe disease symptoms (e.g., fever) in the last month, or used antihypertensive or antidiabetic medications in the NP, and whether they had ever been diagnosed by a doctor with any diseases (e.g., hypertension), or often suffered from any pain currently in CHARLS. People answering positively were categorized as unhealthy, otherwise healthy.

Statistical analysis

First, data from the NP and CHARLS were analyzed separately. We applied one-way ANOVA to examine the differences of CRP in characteristics in each dataset by using F-distribution. The CRP variable was log-transformed because it was not normally distributed. The association between SRH and CRP was estimated by β-coefficient and a 95% confidence interval (CI) using linear regression in two datasets. The first estimate was respective; in the second, datasets were pooled. Fixed-effect meta-analysis was used to examine the heterogeneity. Then we re-ran the linear regression using the pooled dataset.

Age, sex and education were introduced into the basic-adjusted model. Further, we additionally adjusted for marital status, smoking, alcohol consumption, BMI, and health status.[27 28] All analyses were repeated in the stratified analyses by age, sex and levels of education.

In order to compare our results with previous studies that including participant with formal education only, we performed additional linear regression analysis stratified by age and sex among illiterate and literate participants separately.

For peer review only - http://bmjopen.bmj.com/site/about/guidelines.xhtml

All statistical analyses were performed with Stata 13.0 (Stata Corp, College Station, TX, USA).

Patient and public involvement

There were no participants involved in the development of this study.

RESULTS

Characteristics of the participants

The CRP levels across different characteristics of participants were compared in each dataset separately. Table 1 shows that in both datasets that older age people, higher BMI's, poorer SRH, or an unhealthy status were more likely to have elevated levels of CRP. The findings were inconsistent with sex, education, marital status, smoking and alcohol consumption in the two datasets. People with missing CRP values in NP and CHARLS were better educated and reported better health status compared to those who remained in the analyses (Supplementary File: Table S1 and Table S2).

For peer review only - http://bmjopen.bmj.com/site/about/guidelines.xhtml

Table 1 CRP values across characteristics of the study population

	**NP (n=646)**		**CHARLS (n=8555)**	
	Median (IQR) [a]	**P[b]**	Median (IQR) [a]	**P[b]**
Age		<0.001		<0.001
45-60	0.6 (0.3 to 1.2)		0.9 (0.5 to 1.7)	
≥60	0.8 (0.4 to 1.8)		1.1 (0.6 to 2.1)	
Sex		0.011		0.003
Men	0.6 (0.3 to 1.3)		1.0 (0.5 to 2.0)	
Women	0.8 (0.4 to 1.7)		0.9 (0.5 to 1.8)	
Education		0.004		0.316
Illiterate	0.9 (0.4 to 1.8)		1.0 (0.5 to 2.0)	
Literate	0.6 (0.3 to 1.3)		0.9 (0.5 to 1.9)	
Marital status		0.495		<0.001
Married	0.7 (0.3 to 1.5)		0.9 (0.5 to 1.9)	
Non-married	0.7 (0.4 to 1.7)		1.1 (0.6 to 2.4)	
Smoking		0.467		0.041
Current smokers	0.6 (0.3 to 1.4)		1.0 (0.5 to 2.0)	
Non-current smokers	0.7 (0.4 to 1.6)		0.9 (0.5 to 1.9)	
Alcohol consumption		0.001		0.635
Regular drinkers	0.5 (0.3 to 1.1)		0.9 (0.5 to 1.9)	
Non-regular drinkers	0.8 (0.4 to 1.6)		1.0 (0.5 to 1.9)	
BMI		<0.001		<0.001
Underweight (<18.5)	0.5 (0.2 to 1.4)		0.8 (0.5 to 1.9)	
Normal weight (18.5-24.99)	0.6 (0.3 to 1.1)		0.8 (0.5 to 1.7)	

For peer review only

Overweight (25-29.99)	1.2 (0.6 to 2.3)		1.2 (0.7 to 2.3)	
Obese (≥30)	1.6 (1.0 to 4.4)		1.9 (0.9 to 3.3)	
Self-rated health		0.071		<0.001
Good	0.6 (0.3 to 1.7)		0.9 (0.5 to 1.8)	
Average	0.7 (0.3 to 1.5)		0.9 (0.5 to 1.8)	
Poor	0.8 (0.4 to 1.5)		1.0 (0.6 to 2.1)	
Very poor	1.0 (0.5 to 2.3)		1.1 (0.6 to 2.3)	
Health status[c]		0.002		<0.001
Healthy	0.5 (0.3 to 1.3)		0.8 (0.5 to 1.7)	
Unhealthy	0.8 (0.4 to 1.6)		1.0 (0.5 to 2.0)	

[a] Median (interquartile range)

[b] ANOVA was applied to compare the mean of log-transformed values of CRP.

[c] Health status:

Unhealthy: Self-reported moderate to severe symptoms in the last month or used antihypertensive or antidiabetic medications (NP); Had been diagnosed by a doctor with any disease or often suffered from any pain currently (CHARLS). Healthy: no such report.

Missing values:
NP: 1 missing in health status.
CHARLS: 2 missing in age, 7 missing in sex, 4 missing in education,1 missing in smoking, 3 missing in alcohol consumption, 1191 missing in BMI, 65 missing in health status.

For peer review only

SRH and CRP

Table 2 presents the association between SRH and CRP in the two individual populations. In the NP, a borderline statistically significant association was observed between very poor SRH and elevated levels of CRP (β=0.39, 95%CI -0.07 to 0.85) in basic-adjusted model, while the association was attenuated after adjusting for confounders (β=0.29, 95%CI -0.15 to 0.73). Despite insignificance, the estimated effect of SRH started to change direction from average SRH (β = -0.05) to poor SRH (β = 0.10). In CHARLS, poor and very poor SRH were both associated with higher CRP (β=0.06, 95%CI 0.00 to 0.12; β=0.11, 95%CI 0.01 to 0.22). Considering the same pattern in both two datasets that poor and very poor SRH have similar effect on CRP and so as good and average SRH, and that there are limited number of participants with very poor SRH in NP, we combined 'good' and 'average' as good SRH, 'poor' and 'very poor' as poor SRH. Further, we found that poor SRH was associated with higher levels of CRP both in NP (β=0.16, 95%CI -0.02 to 0.34) and CHARLS (β=0.07, 95%CI 0.02 to 0.11) (Table 2).

Table 2 Association between self-rated health and C-reactive protein

		Model1[a]		Model2[b]	
	N	β (95%CI)	P	β (95%CI)	P
**NP**					
Good health	188	Ref.		Ref.	
Average	270	-0.03 (-0.22 to 0.17)	0.792	-0.05 (-0.24 to 0.14)	0.589
Poor	165	0.12 (-0.10 to 0.34)	0.292	0.10 (-0.11 to 0.32)	0.349
Very Poor	23	0.39 (-0.07 to 0.85)	0.093	0.29 (-0.15 to 0.73)	0.202
Good/Poor[c]	458/188	0.17 (-0.01 to 0.35)	0.067	0.16 (-0.02 to 0.34)	0.077
**CHARLS**					
Good health	1794	Ref.		Ref.	
Average	4157	0.01 (-0.04 to 0.06)	0.613	0.00 (-0.05 to 0.06)	0.911
Poor	2157	0.10 (0.04 to 0.15)	0.001	0.06 (0.00 to 0.12)	0.055
Very Poor	447	0.16 (0.06 to 0.25)	0.001	0.11 (0.01 to 0.22)	0.036
Good/Poor	5951/2604	0.10 (0.05 to 0.14)	<0.001	0.07 (0.02 to 0.11)	0.004
**NP+CHARLS**					
Good health	1982	Ref.		Ref.	
Average	4427	0.02 (-0.03 to 0.07)	0.379	0.01 (-0.04 to 0.06)	0.643

For peer review only - http://bmjopen.bmj.com/site/about/guidelines.xhtml

Poor	2322	0.11 (0.05 to 0.16)	<0.001	0.08 (0.02 to 0.14)	0.013
Very Poor	470	0.18 (0.09 to 0.28)	<0.001	0.14 (0.04 to 0.24)	0.007
Good/Poor	6409/2792	0.11 (0.06 to 0.15)	<0.001	0.08 (0.03 to 0.12)	0.001

[a] Adjusted for age, sex, education

[b] Adjusted for age, sex, education, marital status, smoking, alcohol consumption, BMI, health status

[c] Good= Good+Average, Poor=Poor+Very Poor

As the same direction of effect of estimate and a very low level of heterogeneity (I-squared< 0.001%) were observed in the two datasets (data not shown), we pooled the data and re-ran the linear regression analyses in the combined populations. The association between poorer SRH and higher CRP was observed in the pooled population (β=0.08, 95%CI 0.03 to 0.12) (Table2).

The roles of age, sex, and education in the association between SRH and CRP

The association between SRH and CRP stratified by age, sex, education is showed in Figure 2. In middle-aged people, worse SRH was associated with higher CRP both in NP (β=0.42, 95%CI 0.14 to 0.71) and CHARLS (β=0.06, 95%CI -0.01 to 0.12). Among older people, a similar trend was observed in CHARLS (β=0.08, 95%CI 0.02 to 0.15), but not in the NP. When stratified by sex, we found a statistically significant SRH-CRP association among men both in NP (β=0.27, 95%CI -0.03 to 0.57) and CHARLS (β=0.12, 95%CI 0.05 to 0.19), but not in women. In a stratified analysis by education, the association between SRH and CRP was seen in literate people both in NP (β=0.26, 95%CI 0.02 to 0.51) and CHARLS (β=0.11, 95%CI 0.05 to 0.16), but not in illiterate people.

In the pooled population, the SRH-CRP association was repeated in the middle-aged (β=0.08, 95%CI 0.02 to 0.14), older people (β=0.08, 95%CI 0.02 to 0.15), men (β=0.13, 95%CI 0.06 to 0.20), and literate people (β=0.12, 95%CI 0.06 to 0.18) (Figure 2).

Additional analyses

For peer review only

Identical trends with respect to the modifying effect of age and sex on the association between SRH and CRP were observed among literate people, but not among illiterate people (Supplementary File: Table S3).

DISCUSSION

In this study, based on 9201 residents in rural area of China, we found that poor SRH was associated with an elevated level of CRP in middle-aged and older people, especially among the men and literate.

Our finding of the association between poorer SRH and higher CRP level was in line with results from previous studies that included participants at similar age as our study participants.[11 14] Yet, those studies mainly included people living in industrialized countries with higher education, while our participants resided in less developed country with features of low literacy.

Possible pathways linking poor SRH and an elevated level of CRP could be related to psychological stress and health behaviors. Poor SRH may reflect a poor physical (e.g., inaccessibility to health service) and social (e.g., limited social network) environment, which can limit one's coping ability and induce psychological stress. It is known that stress can activate the sympathetic nervous system and the hypothalamic-pituitary-adrenal axis, contributing to the production of stress hormones, which in turn increase the secretion of CRP.[29 30] In addition, people with poor SRH were less likely to have an active lifestyle.[31] Having an inactive lifestyle has been suggested to potentially weaken the immune system and facilitate the inflammation processes through the release of pro-inflammatory adipokines.[32]

For peer review only - http://bmjopen.bmj.com/site/about/guidelines.xhtml

It is notable that poor SRH was associated with an elevated CRP level in literate participants, but not in the illiterate participants, which was consistent with one previous study.[21] Similar findings were also shown in studies focusing on SRH and mortality.[18 19] One of the possible explanations may be that illiterate people are often lack of health-related knowledge and access to health care,[17] and thus may misinterpret the feeling that they have in their bodies.[33] It has been shown that poor SRH in the less educated people mainly represents less serious diseases.[34] In our study, we also found that illiterate people were more likely to rate their health as poor and to report illness or pain both in NP and CHARLS. Moreover, illiterate people may have to withstand more pressure as they have less social and financial resources. Thus, other factors may contribute to the reported poor SRH, rather than actual health condition.

We found that SRH-CRP associations were only observed in men, but not in women, which may be due to the potential sex differences in reporting SRH. Previous studies have shown that the poor SRH in women can reflect both serious and non-serious diseases, whereas it tends to reflect serious diseases in men.[35] Broad dimensions of health perceptions may lead to less accurate SRH in women. In addition, the proportion of illiterate people among women is much higher than that among men in both datasets. This may explain the inconsistent findings between our study (6% participants with more than 9 years of schooling) and the Iwate-KENCO study from Japan, in which the corresponding figure was 46%.[14]

Findings from two datasets were not completely consistent. The association between poor SRH and elevated CRP values among older people (aged ≥60 years) was observed in CHARLS, but not in NP. In both populations, poor SRH was only associated with higher CRP in men, not in women. One of the explanations for these findings may be related to educational levels in the two study populations. Indeed, the proportion of illiterate people was relatively higher in older

For peer review only - http://bmjopen.bmj.com/site/about/guidelines.xhtml

adults in NP (76.2%) than in CHARLS (58.3%), and there was a higher proportion of illiterate people in women in both populations. Second, we observed similar age and sex differences in the associations between SRH and CRP among the literate: poor SRH was associated with elevated CRP values, especially in men, which was the same as the main results. This suggests that education might play a role in the SRH-CRP association.

The strengths of the current study include the objective measure of CRP, the use of two different study populations to increase the confidence of our findings, and the high generalizability of our results to rural population of China given the use of national representative sample, CHARLS.

There are several limitations that should be considered. First, the cross-sectional study design prevented us from making causal inferences. Second, CRP was evaluated using different methods in NP and CHARLS. Nevertheless, the association between SRH and CRP did not differ between the two cohorts. Third, the self-reported SRH and some of the covariates may introduce reporting bias. Fourth, selection bias may arise due to the use of convenience sampling in NP. However, the results from NP were similar to those from CHARLS, which is a national representative sample. Finally, residual confounding or hidden bias cannot be ruled out due to lack of information on some potential confounders, such as clinical cardiovascular risk factors (e.g. HDL-C, HbA1c), acute inflammatory conditions, and medication use.

This study provides evidence that SRH, a simple measurement, may be used as an indicator of bad physical health among middle-aged and older literate people, but not among the illiterate people, in rural area. In China, the implementation of health surveillance is more challenging in rural than in urban areas because of the discrepant aging processes,[36] knowledge gaps[22] and

For peer review only - http://bmjopen.bmj.com/site/about/guidelines.xhtml

income inequality between these two areas. Elevated CRP has been associated with various physical[1-4] and psychological health outcomes.[37 38] Thus, our results support the consideration of using an efficient and cost-effective way, such as SRH, to monitor the health status in rural population where medical resources are limited. Future studies are needed to confirm our results and extend these findings to larger and more diverse populations. Moreover, identification of simple health indictors for illiterate people are warranted.

Acknowledgments We would like to express our sincere gratitude to the participants and local staff in NP project. We are grateful for those supporters: Harvard University (Aki Yazawa); National Center for Global Health and Medicine, Japan (Yosuke Inoue); Nagasaki Prefectural Institute of Environment and Public Health (Guoxi Cai); Fujian Medical University (Fei He, Jie Chen); Fujian Provincial Center for Disease Control and Prevention (Meng Huang) during the data collection in NP. Data from China Health and Retirement Longitudinal Study (CHARLS) were collected by the National School of Development at Peking University, China. We appreciated to University of Copenhagen (Tianwei Xu); Fujian Provincial Center for Disease Control and Prevention (Xiuquan Lin); Nagasaki University (Sabin Nundu) for providing valuable comments in analysis and interpretation of data.

Contributors HW, RT, and KP conceptualized the study. RT analyzed the data and drafted the manuscript. HW, KP, GC, TY contributed to critical revisions of the manuscript. RT and HW are responsible for ensuring the integrity and accuracy of the study. All authors have read and approved the final manuscript.

Funding This study is financed by the Program for Nurturing Global Leaders in Tropical and Emerging Communicable Diseases, Graduate School of Biomedical Sciences, Nagasaki

For peer review only - http://bmjopen.bmj.com/site/about/guidelines.xhtml

University, Japan (Raoping Tu); the Swedish Research Council (Grant no: 2018-02998) and the Swedish Research Council for Health, Working Life and Welfare (Forte) (2019-01120) (Hui-Xin Wang); the Ministry of Education of Taiwan, the Swedish National Graduate School on Ageing and Health (SWEA), and Gamla Tjänarinnor Foundation (Kuan-Yu Pan). NP was financially supported by the JSPS KAKENHI from the Japan Society for the Promotion of Science (13J06172).

For peer review only

Competing Interests None declared.

Patient consent Not required.

Ethics approval The Ethics Committee for Medical Research at the University of Tokyo (No. 10515-(1)) and the Ethics Committee of the Institute of Tropical Medicine at Nagasaki University (No.120910100-5) approved the study protocol of NP. The Medical Ethics Committee of Peking University approved the research protocol of CHARLS.

Provenance and peer review Not commissioned; externally peer reviewed

Data sharing statement All of the CHARLS data will be accessible to researchers around the world at the CHARLS project website (http://charls.pku.edu.cn/en). No additional data available.

Figure legends

Figure 1 Flowchart of the study populations in NP and CHARLS

For peer review only - http://bmjopen.bmj.com/site/about/guidelines.xhtml

Figure 2 β-coefficient and 95% confidence interval (CI) of CRP in relation to poor self-rated health from linear regression models stratified by age, sex and education in NP, CHARLS, and the pooled populations of the two datasets. SRH is dichotomized as poor to very poor versus good to average. When stratified by age, models are adjusted for sex, education, marital status, smoking, alcohol consumption, BMI, health status; when stratified by sex, models are adjusted for age, education, marital status, smoking, alcohol consumption, BMI, health status; when stratified by education, models are adjusted for age, sex, marital status, smoking, alcohol consumption, BMI, health status.

SUPPLEMENTARY FILE

Table S1 Characteristics of study sample in NP without and with missing values in CRP

Table S2 Characteristics of study sample in CHARLS without and with missing values in CRP

Table S3 Association between self-rated health and C-reactive protein among illiterate and literate people: stratified by age and sex

For peer review only - http://bmjopen.bmj.com/site/about/guidelines.xhtml

References

1 Black PH, Garbutt LD. Stress, inflammation and cardiovascular disease. *Journal of psychosomatic research* 2002;52:1-23.

2 Ridker PM. From C-Reactive Protein to Interleukin-6 to Interleukin-1. *Circulation Research* 2016;118:145-56.

3 Barzilaym JI, Freedland ES. Inflammation and its relationship to insulin resistance, type 2 diabetes mellitus, and endothelial dysfunction. *Metabolic syndrome and related disorders* 2003;1:55-67.

4 Wang J, Lee IM, Tworoger SS, *et al*. Plasma C-reactive protein and risk of breast cancer in two prospective studies and a meta-analysis. *Cancer Epidemiol Biomarkers Prev* 2015;24:1199-206.

5 Panza F, Solfrizzi V, Logroscino G, *et al*. Current epidemiological approaches to the metabolic-cognitive syndrome. *J Alzheimers Dis* 2012;30 Suppl 2:S31-75.

6 Idler EL, Kasl SV. Self-ratings of health: do they also predict change in functional ability? *J Gerontol B Psychol Sci Soc Sci* 1995;50:S344-53.

7 Latham K, Peek CW. Self-Rated Health and Morbidity Onset Among Late Midlife U.S. Adults. *The Journals of Gerontology Series B: Psychological Sciences and Social Sciences* 2012;68:107-16.

8 DeSalvo KB, Bloser N, Reynolds K, *et al*. Mortality prediction with a single general self-rated health question. A meta-analysis. *J Gen Intern Med* 2006;21:267-75.

9 Singh-Manoux A, Dugravot A, Shipley MJ, *et al*. The association between self-rated health and mortality in different socioeconomic groups in the GAZEL cohort study. *Int J Epidemiol* 2007;36:1222-8.

For peer review only - http://bmjopen.bmj.com/site/about/guidelines.xhtml

10 Salomon JA, Nordhagen S, Oza S, *et al.* Are Americans feeling less healthy? The puzzle of trends in self-rated health. *Am J Epidemiol* 2009;170:343-51.

11 Christian LM, Glaser R, Porter K, *et al*. Poorer self-rated health is associated with elevated inflammatory markers among older adults. *Psychoneuroendocrinology* 2011;36:1495-504.

12 Janszky I, Lekander M, Blom M, *et al*. Self-rated health and vital exhaustion, but not depression, is related to inflammation in women with coronary heart disease. *Brain Behav Immun* 2005;19:555-63.

13 Shanahan L, Bauldry S, Freeman J, *et al*. Self-rated health and C-reactive protein in young adults. *Brain, Behavior, and Immunity* 2014;36:139-46.

14 Tanno K, Ohsawa M, Onoda T, *et al*. Poor self-rated health is significantly associated with elevated C-reactive protein levels in women, but not in men, in the Japanese general population. *Journal of Psychosomatic Research* 2012;73:225-31.

15 Orre IJ, Reinertsen KV, Aukrust P, *et al*. Higher levels of fatigue are associated with higher CRP levels in disease-free breast cancer survivors. *J Psychosom Res* 2011;71:136-41.

16 Tamura T, Naito M, Maruyama K, et al. The association between self-rated health and high-sensitivity C-reactive protein level: a cross-sectional and 5-year longitudinal study. BMC Public Health 2018;18(1):1380. doi: 10.1186/s12889-018-6251-6

17 Spuling SM, Huxhold O, Wurm S. Predictors of Self-Rated Health: Does Education Play a Role Above and Beyond Age? *J Gerontol B Psychol Sci Soc Sci* 2017;72:415-24.

18 Dowd JB, Zajacova A. Does the predictive power of self-rated health for subsequent mortality risk vary by socioeconomic status in the US? *Int J Epidemiol* 2007;36:1214-21.

19 Huisman M, van Lenthe F, Mackenbach J. The predictive ability of self-assessed health for mortality in different educational groups. *Int J Epidemiol* 2007;36:1207-13.

20 Li Y, Zhong X, Cheng G, et al. Hs-CRP and all-cause, cardiovascular, and cancer mortality risk: A meta-analysis. Atherosclerosis 2017;259:75-82.

For peer review only

21 Dowd JB, Zajacova A. Does self-rated health mean the same thing across socioeconomic groups? Evidence from biomarker data. *Ann Epidemiol* 2010;20:743-9.

22 Guo S, Guo Y. *Spotlight on China: Changes in education under China's market economy*: Springer, 2016.

23 Brindle E, Fujita M, Shofer J, *et al*. Serum, plasma, and dried blood spot high-sensitivity C-reactive protein enzyme immunoassay for population research. *J Immunol Methods* 2010;362:112-20.

24 Zhao Y, Strauss J, Yang G, *et al. China health and retirement longitudinal study–2011–2012 national baseline users' guide*. National School of Development, Peking University, 2013.

25 Yazawa A, Inoue Y, Cai G, *et al*. Epstein-Barr virus antibody titer as a stress biomarker and its association with social capital in rural Fujian communities, China. *Am J Hum Biol* 2018:e23135.

26 Zhao Y, Crimmins E, Hu P, *et al*. *CHARLS Blood Sample Users' Guide*. National School of Development, Peking University, 2014.

27 Kiecolt-Glaser JK, Gouin JP, Hantsoo L. Close relationships, inflammation, and health. *Neurosci Biobehav Rev* 2010;35:33-8.

28 Zheng H, Thomas PA. Marital status, self-rated health, and mortality: overestimation of health or diminishing protection of marriage? *Journal of health and social behavior* 2013;54:128-43.

29 Coussons-Read ME, Okun ML, Nettles CD. Psychosocial stress increases inflammatory markers and alters cytokine production across pregnancy. *Brain Behav Immun* 2007;21:343-50.

30 Shivpuri S, Gallo LC, Crouse JR, *et al.* The association between chronic stress type and C-reactive protein in the multi-ethnic study of atherosclerosis: does gender make a

For peer review only - http://bmjopen.bmj.com/site/about/guidelines.xhtml

difference? *J Behav Med* 2012;35:74-85.

31 Molarius A, Berglund K, Eriksson C, *et al*. Socioeconomic conditions, lifestyle factors, and self-rated health among men and women in Sweden. *European journal of public health* 2007;17:125-33.

32 Gleeson M, Bishop NC, Stensel DJ, *et al*. The anti-inflammatory effects of exercise: mechanisms and implications for the prevention and treatment of disease. *Nat Rev Immunol* 2011;11:607-15.

33 Franks P, Gold MR, Fiscella K. Sociodemographics, self-rated health, and mortality in the US. *Social Science & Medicine* 2003;56:2505-14.

34 Regidor E, Guallar-Castillon P, Gutierrez-Fisac JL, *et al.* Socioeconomic variation in the magnitude of the association between self-rated health and mortality. *Ann Epidemiol* 2010;20:395-400.

35 Benyamini Y, Leventhal EA, Leventhal H. Gender differences in processing information for making self-assessments of health. *Psychosomatic Medicine* 2000;62:354-64.

36 Gong P, Liang S, Carlton EJ, *et al.* Urbanisation and health in China. *The Lancet* 2012;379(9818):843-52.

37 Valkanova V, Ebmeier KP, Allan CL. CRP, IL-6 and depression: a systematic review and meta-analysis of longitudinal studies. *J Affect Disord* 2013;150(3):736-44.

38 Wium-Andersen MK, Orsted DD, Nielsen SF, *et al.* Elevated C-reactive protein levels, psychological distress, and depression in 73, 131 individuals. *JAMA Psychiatry* 2013;70(2):176-84.

For peer review only - http://bmjopen.bmj.com/site/about/guidelines.xhtml

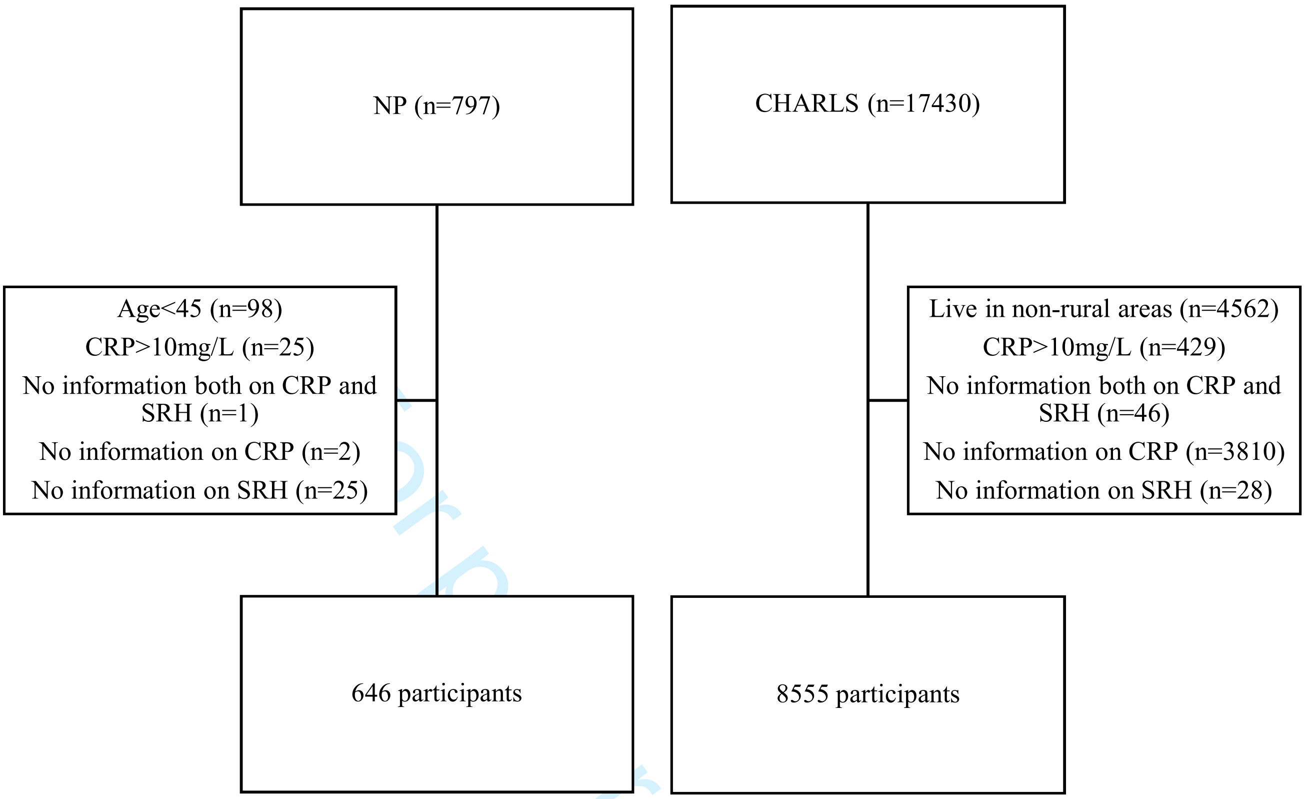

Fig 1 Flowchart of the study populations in NP and CHARLS

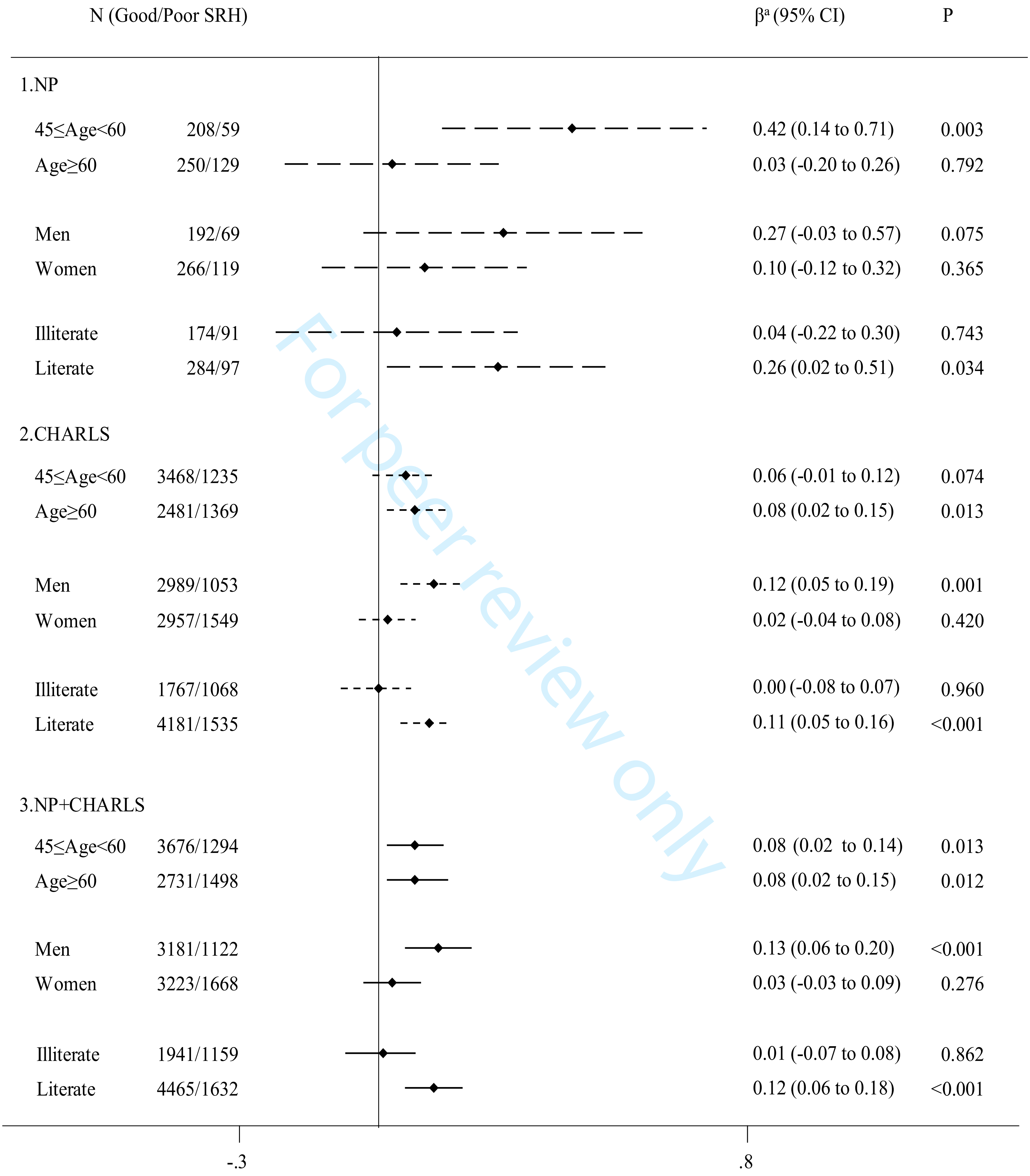

Figure 2 β-coefficient and 95% confidence interval (CI) of CRP in relation to poor self-rated health from linear regression models stratified by age, sex and education in NP, CHARLS, and the pooled populations of the two datasets. SRH is dichotomized as poor to very poor versus good to average. When stratified by age, models are adjusted for sex, education, marital status, smoking, alcohol consumption, BMI, health status; when stratified by sex, models are adjusted for age, education, marital status, smoking, alcohol consumption, BMI, health status; when stratified by education, models are adjusted for age, sex, marital status, smoking, alcohol consumption, BMI, health status.

[a] The average CRP changes in response to one-unit shift in SRH.

For peer review only

SUPPLEMENTARY FILE

Table S1 Characteristics of study sample in NP without and with missing values in CRP

	Non-missing[a] (n=646)	Missing[a] (n=2)	P
Age			0.093
<60	267(41.3)	2(100)	
≥60	379(58.7)	0(0)	
Sex			0.245
Men	261(40.4)	0(0)	
Women	385(59.6)	2(100)	
Education			0.239
Illiterate	265(41.0)	0(0)	
Literate	381(59.0)	2(100)	
Marital status			0.491
Married	522(80.8)	2(100)	
Non-married	124(19.2)	0(0)	
Smoking			0.582
Current smokers	85(13.2)	0(0)	
Non-current smokers	561(86.8)	2(100)	
Drinking			0.489
Regular drinkers	125(19.4)	0(0)	
Non-regular drinkers	521(80.7)	2(100)	
BMI			0.810
Underweight (<18.5)	30(4.60)	0(0)	
Normal weight (18.5-25)	436(67.5)	2(100)	
Overweight (25-30)	158(24.5)	0(0)	
Obese (≥30)	22(3.4)	0(0)	
Self-rated health			0.184
Good	188(29.1)	2(100)	
Average	270(41.8)	0(0)	
Poor	165(25.5)	0(0)	
Very poor	23(3.6)	0(0)	
Health status[b]			0.018
Healthy	127(19.7)	2(100)	
Unhealthy	518(80.2)	0(0)	
Missing	1(0.2)	0(0)	

[a] Data are presented as n (%).

[b] Healthy status:

Unhealthy: Self-reported moderate to severe symptoms in the last month or used antihypertensive or antidiabetic medications (NP).

Healthy: No such report.

For peer review only

Table S2 Characteristics of study sample in CHARLS without and with missing values in CRP

	Non-missing[a] (n=8555)	Missing[a] (n=3810)	P
Age			0.002
<60	4703(55.0)	2226(58.4)	
≥60	3850(45.0)	1583(41.6)	
Missing	2(0)	1(0)	
Sex			<0.001
Men	4042(47.3)	2014(52.9)	
Women	4506(52.7)	1794(47.1)	
Missing	7(0.1)	2(0.1)	
Education			0.001
Illiterate	2835(33.1)	1160(30.5)	
Literate	5716(66.8)	2643(69.4)	
Missing	4(0.1)	7(0.2)	
Marital status			0.001
Married	7517(87.9)	3263(85.6)	
Non-married	1038(12.1)	547(14.4)	
Smoking			0.113
Current smokers	2561(29.9)	1086(28.5)	
Non-current smokers	5993(70.1)	2722(71.4)	
Missing	1(0)	2(0.1)	
Drinking			0.024
Regular drinkers	998(11.7)	399(10.5)	
Non-regular drinkers	7554(88.3)	3406(89.4)	
Missing	3(0)	5(0.1)	
BMI			<0.001
Underweight (<18.5)	535(6.3)	206(5.4)	
Normal weight (18.5-25)	4719(55.2)	1790(47.0)	
Overweight (25-30)	1819(21.3)	592(15.5)	
Obese (≥30)	291(3.4)	101(2.7)	
Missing	1191(13.9)	1121(29.4)	
Self-rated health			0.002
Good	1794(21.0)	910(23.9)	
Average	4157(48.6)	1798(47.2)	
Poor	2157(25.2)	894(23.5)	
Very poor	447(5.2)	208(5.5)	
Health status[b]			<0.001
Healthy	2089(24.4)	1160(30.5)	
Unhealthy	6401(74.8)	2607(68.4)	
Missing	65(0.8)	43(1.1)	

[a] Data are presented as n (%)
[b] Healthy status:
Unhealthy: Had been diagnosed by a doctor with any disease or often suffered from any pain currently (CHARLS).
Healthy: No such report.

For peer review only

Table S3 Association between self-rated health and C-reactive protein among illiterate and literate people: stratified by age and sex

	N	Age<60	N	Age≥60	N	Men	N	Women
**Illiterate**								
**NP**								
Good	47	Ref.	127	Ref.	56	Ref.	118	Ref.
Poor	16	0.35 (-0.27 to 0.97)	75	-0.01 (-0.32 to 0.29)	21	0.03(-0.53 to 0.59)	70	0.04 (-0.26 to 0.34)
**CHARLS**								
Good	777	Ref.	989	Ref.	406	Ref.	1359	Ref.
Poor	405	-0.07 (-0.19 to 0.05)	663	0.05 (-0.05 to 0.15)	232	-0.06(-0.24 to 0.11)	835	0.02 (-0.07 to 0.1)
**NP+CHARLS**								
Good	824	Ref.	1116	Ref.	462	Ref.	1477	Ref.
Poor	421	-0.05 (-0.17 to 0.07)	738	0.05 (-0.05 to 0.14)	253	-0.04(-0.2 to 0.13)	905	0.02 (-0.06 to 0.1)
**Literate**								
**NP**								
Good	161	Ref.	123	Ref.	136	Ref.	148	Ref.
Poor	43	0.47 (0.14 to 0.8)**	54	0.08 (-0.3 to 0.45)	48	0.4 (0.03 to 0.77)*	49	0.17 (-0.16 to 0.5)
**CHARLS**								
Good	2690	Ref.	1490	Ref.	2581	Ref.	1597	Ref.
Poor	829	0.11 (0.03 to 0.19)**	706	0.11 (0.02 to 0.19)*	821	0.16 (0.08 to 0.24)**	713	0.03 (-0.05 to 0.12)
**NP+CHARLS**								
Good	2851	Ref.	1613	Ref.	2717	Ref.	1745	Ref.
Poor	872	0.13 (0.06 to 0.21)**	760	0.11 (0.02 to 0.19)*	869	0.17 (0.1 to 0.25)**	762	0.04 (-0.04 to 0.13)

CHARLS: 1) Iliterate:1 missing in age, 3 missing in sex; 2) Literate: 1 missing in age, 4 missing in sex.
*P<0.05
**P<0.01

For peer review only

STROBE 2007 (v4) Statement—Checklist of items that should be included in reports of *cross-sectional studies*

Section/Topic	Item #	Recommendation	Reported on page #
**Title and abstract**	1	(*a*) Indicate the study's design with a commonly used term in the title or the abstract	P1
		(*b*) Provide in the abstract an informative and balanced summary of what was done and what was found	P3
**Introduction**			
Background/rationale	2	Explain the scientific background and rationale for the investigation being reported	P4-5
Objectives	3	State specific objectives, including any prespecified hypotheses	P5-6
**Methods**			
Study design	4	Present key elements of study design early in the paper	P6-7
Setting	5	Describe the setting, locations, and relevant dates, including periods of recruitment, exposure, follow-up, and data collection	P6-9
Participants	6	(*a*) Give the eligibility criteria, and the sources and methods of selection of participants	P6-7
Variables	7	Clearly define all outcomes, exposures, predictors, potential confounders, and effect modifiers. Give diagnostic criteria, if applicable	P7-9
Data sources/ measurement	8*	For each variable of interest, give sources of data and details of methods of assessment (measurement). Describe comparability of assessment methods if there is more than one group	P7-9
Bias	9	Describe any efforts to address potential sources of bias	P9
Study size	10	Explain how the study size was arrived at	P6-7
Quantitative variables	11	Explain how quantitative variables were handled in the analyses. If applicable, describe which groupings were chosen and why	P8-9
Statistical methods	12	(*a*) Describe all statistical methods, including those used to control for confounding	P9
		(*b*) Describe any methods used to examine subgroups and interactions	P9
		(*c*) Explain how missing data were addressed	P10,12
		(*d*) If applicable, describe analytical methods taking account of sampling strategy	P6-7
		(*e*) Describe any sensitivity analyses	P9
**Results**			

For peer review only

Participants	13*	(a) Report numbers of individuals at each stage of study—eg numbers potentially eligible, examined for eligibility, confirmed eligible, included in the study, completing follow-up, and analysed	Figure 1
		(b) Give reasons for non-participation at each stage	Figure 1
		(c) Consider use of a flow diagram	Figure 1
Descriptive data	14*	(a) Give characteristics of study participants (eg demographic, clinical, social) and information on exposures and potential confounders	P10-12
		(b) Indicate number of participants with missing data for each variable of interest	P12
Outcome data	15*	Report numbers of outcome events or summary measures	P11-12
Main results	16	(*a*) Give unadjusted estimates and, if applicable, confounder-adjusted estimates and their precision (eg, 95% confidence interval). Make clear which confounders were adjusted for and why they were included	P13-14, why: P9
		(*b*) Report category boundaries when continuous variables were categorized	P8-9
		(*c*) If relevant, consider translating estimates of relative risk into absolute risk for a meaningful time period	N/A
Other analyses	17	Report other analyses done—eg analyses of subgroups and interactions, and sensitivity analyses	P15
**Discussion**			
Key results	18	Summarise key results with reference to study objectives	P15
Limitations	19	Discuss limitations of the study, taking into account sources of potential bias or imprecision. Discuss both direction and magnitude of any potential bias	P4, P17
Interpretation	20	Give a cautious overall interpretation of results considering objectives, limitations, multiplicity of analyses, results from similar studies, and other relevant evidence	P15-17
Generalisability	21	Discuss the generalisability (external validity) of the study results	P17-18
**Other information**			
Funding	22	Give the source of funding and the role of the funders for the present study and, if applicable, for the original study on which the present article is based	P18-19

*Give information separately for cases and controls in case-control studies and, if applicable, for exposed and unexposed groups in cohort and cross-sectional studies.

Note: An Explanation and Elaboration article discusses each checklist item and gives methodological background and published examples of transparent reporting. The STROBE checklist is best used in conjunction with this article (freely available on the Web sites of PLoS Medicine at http://www.plosmedicine.org/, Annals of Internal Medicine at http://www.annals.org/, and Epidemiology at http://www.epidem.com/). Information on the STROBE Initiative is available at www.strobe-statement.org.

For peer review only
